# Single-nucleus epigenomic dysregulation unmasks genetic risk-associated neurodegenerative glia states

**DOI:** 10.1038/s41467-026-73007-1

**Published:** 2026-05-14

**Authors:** Xia Han, Gregory M. Rosenberg, Vivianne M. Kisling, Tao Zhang, Chia-Yi Lee, Ashvin Ravi, Mikhail Melnik, Tina Bilousova, Salvatore Spina, Alissa L. Nana, Lea T. Grinberg, William W. Seeley, Karen H. Gylys, Laura M. Huckins, Towfique Raj, Kristen J. Brennand, Jessica E. Rexach

**Affiliations:** 1 Program in Neurogenetics, Department of Neurology, David Geffen School of Medicine, University of California, Los Angeles, Los Angeles, CA USA; 2 Department of Genetics, Wu Tsai Institute, Yale University School of Medicine, New Haven, CT USA; 3 Department of Genetics and Genomics, Icahn School of Medicine at Mount Sinai, New York, NY USA; 4 Nash Family Department of Neuroscience, Friedman Brain Institute, Icahn School of Medicine at Mount Sinai, New York, NY USA; 5 Department of Physiological Nursing, School of Nursing, University of California, San Francisco, San Francisco, CA USA; 6 Department of Neurology, Fein Memory and Aging Center, University of California, San Francisco, San Francisco, CA USA; 7 Department of Pathology, University of California, San Francisco, San Francisco, CA USA; 8 Department of Psychiatry, Division of Molecular Psychiatry, Yale University School of Medicine, New Haven, CT USA; 9 Ronald M. Loeb Center for Alzheimer’s Disease, Icahn School of Medicine at Mount Sinai, New York, NY USA; 10 Friedman Brain Institute, Icahn School of Medicine at Mount Sinai, New York, NY USA; 11 Department of Human Genetics, David Geffen School of Medicine, University of California, Los Angeles, Los Angeles, CA USA

**Keywords:** Epigenomics, Gene regulation, Epigenetics, Dementia

## Abstract

The accumulation of abnormal tau protein selectively affects distinct brain regions and specific populations of neurons and glial cells in tau-related dementias, such as Alzheimer’s disease, Pick’s disease and progressive supranuclear palsy. Although the three disorders share the feature of tau protein pathology, the regulatory circuitry of non-coding genetic variants underlying risk-associated cell states remains to be elucidated. Using paired single-nucleus profiling of chromatin accessibility and gene expression across the three conditions, we define cell-type-specific cis-regulatory elements across six cell types and fifty subclasses. Comparing disease-dynamic cis-regulatory elements across three disorders, we find that glia overrepresent disorder-specific gene regulation related to dynamic cellular response to stress. We show that human genetic variants affecting microglial gene regulation converge into distinct and co-regulated modules affecting specific cellular functions. Moreover, polygenic risk modifiers are maximally co-accessible in disorder-specific glial states, modifying distinct pathways such as sphingomyelin regulation in Pick’s disease. Our study informs glial regulators linked to polygenic modifiers of primary tauopathy, establishing modifiable pathways governing resilience.

## Abstract (web summary)

Accumulation of abnormal tau protein selectively affects specific brain cell populations in tau-related dementias. Here, the authors use single nuclei data to show how genetic risk is linked to regional vulnerability across tau dementias.

## Introduction

Neurodegenerative tauopathies are characterized by abnormal tau aggregation in the brain and are associated with symptoms of dementia and parkinsonism. Pick’s disease (PiD) and progressive supranuclear palsy (PSP) are primary tauopathies, where tau is the major component of the pathology. Alzheimer’s disease (AD) is characterized by tau aggregation, which is widely thought to be accelerated by the pathological protein amyloid-beta. Tauopathies share pathological tau aggregation but differ in symptoms, pathological tau forms^1^, and genetic architecture^2^. Neuropathological characteristics distinguish each disorder, including the specific brain regions and cell types most affected by tau pathology and degeneration, and the structure of self-propagating tau fibrils^1,3^. Genetic risk factors further distinguish each disorder, including several examples of variants with opposing effects on disease risk^4–8^. Investigating several diseases in parallel will facilitate our understanding of shared vs. distinct mechanisms linked to disorder-specific genetic and pathological factors across tauopathies.

Although the selective vulnerability of neurons is a major pathological hallmark of disease, glial cells, which maintain and support neuronal function, are increasingly recognized as dysregulated in neurodegenerative diseases. Recent single-cell genomics studies of AD have revealed disrupted signaling pathways across multiple glial cell types, including those involved in inflammation and immune responses, lipid signaling, metabolic stress, and DNA damage^9–16^. Among glia, microglia have attracted the most attention due to their disproportionate expression of AD-associated risk^17–21^. Microglial expression of several of these AD risk genes has been associated with beta-amyloid^19,22–25^. In contrast, glial tau lesions in astrocytes and oligodendrocytes are hallmark features of other tauopathies^1,26,27^. To understand the underlying regulatory mechanisms, large-scale single-cell transcriptomic and epigenomic profiling studies have begun to characterize widespread remodeling of cis-regulatory elements and transcription factor (TF) programs in vulnerable brain cell types in AD and related tauopathies^10,17,18,28–33^. We recently demonstrated diverse shared and distinct cellular responses to three tau-associated disorders, including an Alzheimer’s-enriched microglia state with high expression of genes associated with AD polygenic risk together with genes known to protect against AD-specific pathology^19,34^. It remains unclear whether glial subpopulations have distinct mechanisms underlying their diverse roles in disease pathogenesis across different tau dementia disorders, and how disease-associated chromatin remodeling integrates genetic risk and regulatory variation across different tauopathies and brain regions. Specifically, tau pathology-associated glial states in primary tauopathies remain largely uncharacterized with respect to disease-specific genetics, associated gene regulatory pathways, and downstream functions.

To gain a comprehensive understanding of the epigenomic reactivity and dysregulation in glial subtypes in tauopathies, we conducted an in-depth investigation using single-nucleus profiling of chromatin accessibility paired with gene expression analysis across AD, PiD, and PSP in three brain regions with distinct vulnerability (precentral gyrus, insula, and calcarine cortex). By integrating genome-wide association study (GWAS) summary statistics with cell-type-specific chromatin accessibility data obtained across disorders, we enhanced mapping of GWAS variants to functional elements in the human genome and revealed that dynamic changes in chromatin accessibility contribute to explaining disease heritability. Our analysis revealed that the disease heritability of PiD and PSP involved distinct reactive microglia and astrocytes, converging on states exhibiting shared transcriptional regulators, non-coding circuits relevant to lysosomal activation, and alterations in sphingomyelin regulation. These findings introduce genetic-risk-related glial modifiers associated with resilience to tau pathology.

## Results

**Fig. 1:**
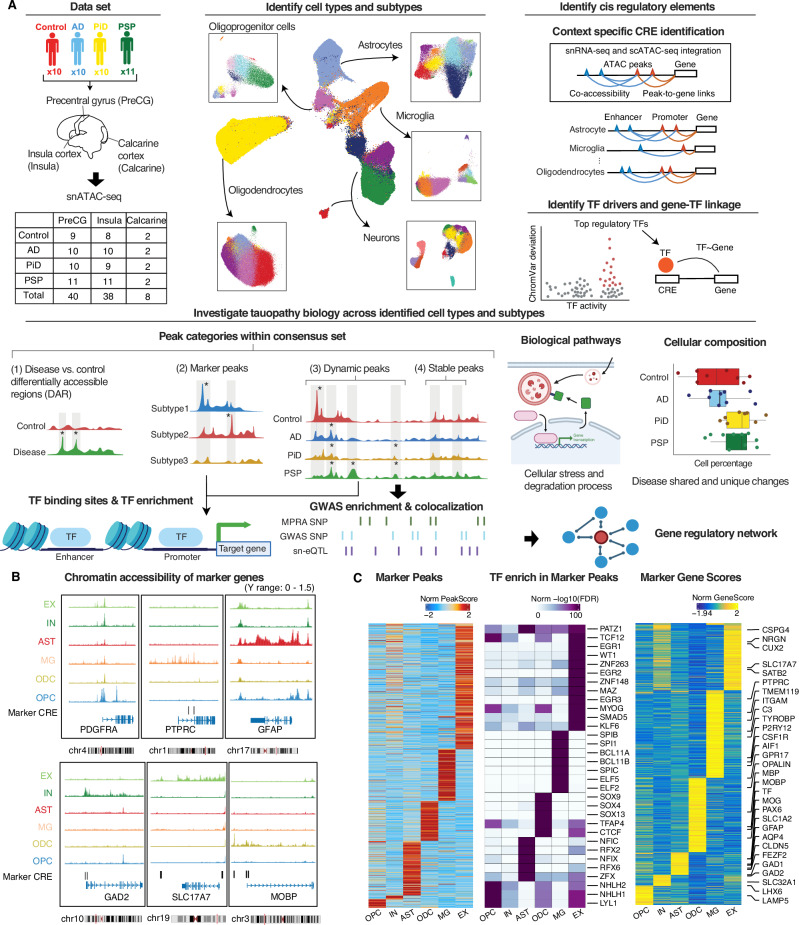
Single-nucleus epigenomic landscape of AD, PiD and PSP across brain regions. **A** Schematic overview of the snATAC-seq analysis workflow. Created in BioRender. Han, X. (https://BioRender.com/7adtg7s). **B** Tracks of chromatin accessibility profiles generated using pseudo-bulk data for each cell type at canonical marker genes. Marker cis-regulatory elements (CREs) of 500 bp are labeled. Visualization and modifications were performed using the UCSC Genome Browser. **C** Heatmaps displaying identified marker peaks (left), marker gene scores (right), and TFs enriched in marker peaks (middle) for each cell type.

To characterize cellular heterogeneity and epigenomic differences underlying the progression of AD, PiD, and PSP across brain regions, we performed single-nucleus ATAC sequencing (snATAC-seq) on 41 individuals (10 controls, 10 AD, 10 PiD, 11 PSP) across three brain regions: calcarine of the visual cortex (calcarine), insular cortex (insula), and precentral gyrus of the frontal cortex (PreCG) using the 10X Genomics Chromium platform (Fig. 1A). These three brain regions show differential vulnerability to disease which we previously characterized under semi-quantitative ratings of pathology burden^19^. The insular cortex showed the highest disease burden in PiD, while the PreCG exhibited the highest burden in PSP; in contrast, the calcarine showed low pathology across all three disorders^19^ (Supplementary Data 1). This generated a total of 86 samples, comprising 682,667 high-quality individual nuclei after quality control (Supplementary Figs. 1 and 2C and Methods). The overall analysis workflow is shown in Fig. 1A. We constructed 8 major clusters after removing 2 undefined groups. The six main cell types were initially annotated based on unambiguous canonical marker expression (Fig. 1B-C, and Supplementary Figs. 2A-2B, and Methods), with 2 excitatory and 2 inhibitory major groups. Marker peaks identified within each cell type showed cell-type-specific enrichment for transcription factors (TF). To map chromatin accessibility states to gene expression profiles, enabling cell type validation and deeper insights into cellular regulation, we integrated snATAC-seq with our in-house snRNA-seq using ArchR’s addGeneIntegrationMatrix function^35^. The snATAC-derived major clusters consistently matched snRNA-derived cell types (Supplementary Fig. 2D), and their cell distribution across brain regions was comparable (Supplementary Fig. 2E). As calcarine samples were limited and showed variability, we focused on the other two regions in the downstream analysis (Methods).

Transcription factors are key upstream regulators of gene expression, maintaining cellular viability and functional networks. Changes in TF activity within specific cell types can reflect their regulatory roles and highlight disease drivers. To investigate this, we identified TFs that regulated gene expression and were linked to motif variability in chromatin accessibility (Supplementary Fig. 3A, left panel, and Methods). TFs specific to distinct cell types, such as *SPI1* and *ELF4* in microglia, *NFIC* in astrocytes, *NEUROD2* and *NEUROD6* in neurons, *PBX3*, *SOX4*, and *SOX13* in oligodendrocytes, exhibit consistent patterns of activity, expression, and motif variability (Supplementary Fig. 3A, right panel). Notably, TF variability in PSP glia deviates from other diseases, with glial types clustering naturally within PSP rather than grouping by cell types across diseases. For example, reduced variability in *FOS* and *JUN* was observed across all glial types in PSP (Supplementary Fig. 3B and Methods), suggesting a general PSP-specific TF activation pattern in glia. This pattern appears to follow a gradient linked to both tau form and pathological severity. The more pronounced clustering in PSP and PiD relative to AD may reflect their predominant tau pathology and the greater severity of affected regions. This supports the interpretation that TF activity aligns with a regulatory gradient coupled to disease type and extent. Conversely, excitatory neurons showed similar TF variability across all diseases, indicating that this regulatory gradient is largely glial-specific.

### Cell type-specific CREs define cell type identity

**Fig. 2:**
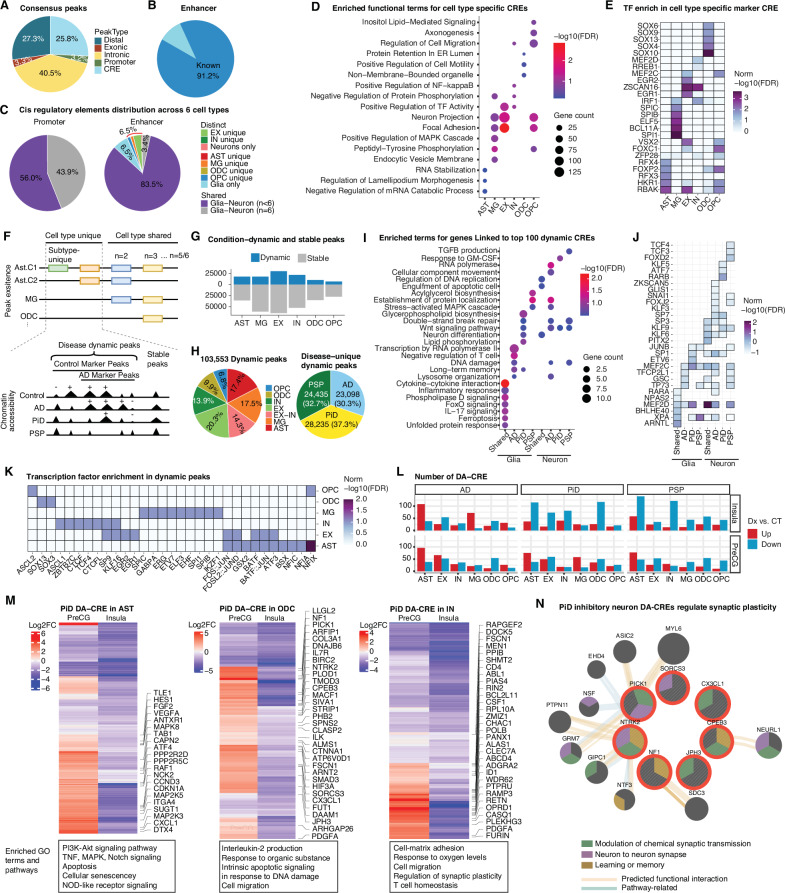
Condition-dynamic and case-control differentially accessible CREs across human brain cell types. **A** Distribution of consensus peaks across genomic contexts (CRE, promoter, intronic, exonic, or distal regions). **B** Pie chart depicting identified enhancers, categorized as known or unannotated. **C** Distribution of promoter peaks (left) and enhancer peaks (right) across cell types. **D** Enriched Gene Ontology (GO) terms for genes linked to cell type-specific CRE, analyzed using enrichR^82^. Significant terms were identified in specific cell types (FDR < 0.1). Astrocytes and oligodendrocytes did not meet this threshold; therefore, their top three enriched terms based on nominal *P* value (*p* < 0.005) are shown to illustrate functional trends. The FDR-corrected version of this anlaysis is provided in Supplementary Fig. 17D. **E** Enrichment of TF motifs in the top 100 marker CREs uniquely identified in specific cell types, analyzed using MEME^42^. **F** Schematic overview of the identification of condition-dynamic peaks for each cell type. **G** Number of dynamic and stable peaks across cell types. **H** Distribution of dynamic peaks across cell types (left) and across diseases (right). **I** Functional enrichment of genes associated with the top 100 dynamic CREs, either unique or shared among the three diseases, in glia or neurons. **J** TF enrichment for dynamic CREs in groups shown in (**I**). **K** TF enrichment in dynamic peaks across cell types. **L** Number of differentially accessible CREs per disease across cell types, divided into up-regulated and down-regulated peaks ( | Log2FC | ≥1.2, *P* < =1e − 3). **M** Heatmaps of PiD DA-CREs up-regulated in the PreCG and down-regulated in the Insula, shown for astrocytes (left), oligodendrocytes (middle), and inhibitory neurons (right). Enriched functional pathways of genes linked to these CREs are shown below. **N** Gene network of synaptic plasticity-regulating genes involved in the PiD DA-CREs transition across regions in inhibitory neurons. Genes linked to PiD DA-CREs are highlighted within the red circle. The network is constructed using GeneMANIA^104^. DA-CREs: differentially accessible cis-regulatory elements. Multiple testing correction was performed using the Benjamini–Hochberg false discovery rate method. Source data are provided as a Source Data file.

Measuring chromatin accessibility on a genome-wide scale enables the definition of potential regulatory elements and illustrates how epigenomic features shape gene expression programs. Using ArchR’s peak calling strategy, we identified a consensus peak set with a total of 924,225 peaks (each 500 bp) for all subclusters. To determine which peaks are candidate cis-regulatory elements (CREs) for genes, we combined the peak co-accessibility with gene-peak correlation from snRNA-seq data to infer CREs in cell subclusters split by disease (Methods). We detected 223,710 enhancers and 14,416 promoters. In this case, 25.8% (238,126) of peaks were defined as CREs, improving the interpretation of 7.3% of distal peaks (Fig. 2A and Supplementary Fig. 4A).

To validate our candidate enhancers, we collected 11 reference enhancers from public resources, including ENCODE (Encyclopedia of DNA Elements)^36^, activity-by-contact (ABC) model predictions^37^, FANTOM5 (Functional Annotation of Mammalian Genomes 5)^38,39^, and single-cell studies^17,32,40^. Based on loci overlapping, we computed the percentages of reference enhancers that were found in our study. As expected, we observed a higher overlapping percentage with known enhancers from single-cell studies^17,32,40^ than ENCODE bulk data (Supplementary Fig. 4B). Relying solely on H3K27ac captured 42.41% of CREs (Supplementary Fig. 4B), whereas the ENCODE cCRE reference, constructed from over 1000 cells and tissues, supported 81.6% of our identified CREs. In total, 91.2% of our enhancers were supported with the reference enhancers (Fig. 2B and Supplementary Fig. 4B).

To evaluate the functional relevance of our disease-specific CREs, we compared them with active chromatin states in the human brain from the ENCODE. These states include EnhA, EnhG, EnhWk, EnhBiv, TssA, TssFlnk, TssFlnkU, TssFlnkD, and TssBiv, which were identified by integrating multiple epigenomic datasets to categorize genomic regions with similar chromatin signatures (Methods). Across cell types, >30% of CREs overlapped these active states (Supplementary Fig. 5), consistent with their regulatory potential. Moderate overlap is expected because ENCODE annotations derive from bulk brain tissue rather than cell-type- or disease-specific profiles, limiting the detection of CREs that are activated only under pathological conditions.

Sets of persistent epigenetic features establish and maintain specific cell types that share a common genome, where gene promoters are broadly accessible across various cell types, and enhancers are often restricted to particular cell types^32,41^. We asked how chromatin accessibility in CRE is regulated across cell types. As expected, enhancers were more cell type-specific, while promoters were generally shared among cell types (Fig. 2C). Our findings show that 6.5% of enhancers were unique to only one cell type, and another 6.5% were specific to glia. Functional enrichment of cell type-specific CRE-linked genes revealed the general functions associated with the corresponding cell type identity (Fig. 2D). For example, astrocyte-specific peaks were enriched in regulators of lamellipodia; microglia-specific peaks related to endocytic vesicle membranes; oligodendrocyte-specific peaks were enriched in organelle assembly and cell motility; and OPC-specific peaks enriched in focal adhesion and axonogenesis. Furthermore, excitatory neuron-specific peaks were involved in the regulation of transcription factor activity and neuron projection, while inhibitory neuron-specific peaks unexpectedly related to cell apoptotic process and immune cell activation. Additionally, the cell type-specific CREs were driven by specific transcription factors (TFs) as expected, such as the highest activation of the *SOX10* family in oligodendrocytes and *SPI1* in microglia (Fig. 2E). Importantly, the proportion of annotated enhancers did not differ when comparing our dynamic to stable peaks, and the overall confidence in peak annotation was matched between peak sets.

### Dynamic changes in chromatin accessibility occur across conditions in specific cell types

The epigenomic landscape dynamically controls gene expression in a context-specific manner. Understanding how chromatin accessibility is altered across variable disease-associated contexts elucidates the specific pathways defined by the transcriptional drivers of diverse cellular states and functions. First, we identified cell type-specific peaks in the consensus peak set, and we found that 48.7% of peaks were unique in five major cell types (Supplementary Fig. 4C). Next, we identified the dynamic and stable peaks for each subcluster based on whether a peak was a marker for a specific condition (Fig. 2F, Methods, and Supplementary Table 1). We found that, on average, 16.1% of all cell type-specific peaks were dynamically changed across conditions in different subclusters (Supplementary Fig. 4D). Of the total 103,553 dynamic peaks, approximately half were found in neurons and half in glia, with disease-specific dynamic peaks distributed relatively evenly across all three disorders (Fig. 2H). When analyzed at the cell type level, the average proportion of cell type-specific peaks that were dynamic across conditions increased to 24%, with astrocytes exhibiting the highest percentage of dynamic peaks at 33% (Fig. 2G). This suggests that astrocytes in particular exhibit pronounced differences in epigenomic dysregulation or epigenomic state transitions when comparing PSP, PiD, and AD.

To elucidate common and distinct biological pathways affected by epigenomic dynamics across disorders and cell types, we performed gene ontology analysis on genes linked to dynamic CREs in glia and neurons (Fig. 2I). In glia, disease-shared dynamic CREs were predominantly related to immune regulation, including the inflammatory response and cytokine−cytokine receptor interaction, reflecting a common immune activation in glial cells across conditions. In neurons, disease-shared dynamic CREs were involved in apoptosis and neuron differentiation, and additional disease-unique dynamic peaks regulated stress-related processes, including Wnt signaling and double-strand DNA break repair, which were shared across disorders. Notably, disease-shared and -distinct dynamic CREs and associated biological pathways were more diverse among glia. For example, disease-shared dynamic CREs in glia regulated inflammation, ER stress, and ferroptosis. In contrast, differentially dynamic CREs in PiD and PSP glia regulated lipid metabolism, compared to AD glia which participated more in the regulation of genes involved in T cell activation (Fig. 2I).

To identify the upstream TF drivers of shared and distinct chromatin dynamics, we next performed a TF motif enrichment analysis using MEME^42^, with disease dynamic peaks as the foreground and stable peaks as the background. For the TF analysis on dynamic CREs in glia and neurons in each disease, we found TFs from the SP and KLF families were enriched in neurons shared across diseases, likely influencing neuronal differentiation and apoptosis (Fig. 2J). In contrast, one of the TFs uniquely enriched in glia is *BHLHE40*, which was known to drive disease-associated microglia response in AD^43^. To investigate TFs driving dynamic peaks in specific cell types across all diseases, we focused on the top 10 enriched motifs in each cell type and found that disease-associated TFs significantly regulate accessibility dynamics (Fig. 2K). For instance, *PU1* (*Spi1*) and *GABPA* were highly enriched in microglia. *SPI1* was known as a key regulator of microglia activation and AD risk^30,44,45^, and *GABPA* was upregulated in PSP and enriched at PSP GWAS-associated disrupted functional variants^46^. This suggested that while neurons engaged in relatively conserved stress responses across disorders, glial cells displayed more dynamic gene regulation to govern diverse and context-responsive biology.

### DARs indicate a transition in gene activation from middle to high pathology regions

The dynamic accessibility analysis captures broad chromatin remodeling trends across cell types and disease states. To complement this, we performed pairwise comparisons of disease and control samples to identify differentially accessible regions (DARs) at the cell type level, aiming to determine which cell type exhibits the most significant accessibility changes associated with disease (Methods). DARs were identified using the Wilcoxon test in ArchR on the discovery set. To account for the impact of nuclei size on disease outcomes and to ensure the robustness of DAR calling, we performed down sampling 10 times, randomly selecting 30 nuclei per sample for each cell type, and repeated the differential analysis using the Wilcoxon test. In the discovery set, the cell-type DARs have a similar peak type distribution to the consensus peaks (Supplementary Fig. 4E), and 91.6% of the differential enhancers were validated (Supplementary Fig. 4F). Since CREs are more likely to have regulatory potential linked to their associated genes, we focused on differentially accessible CREs (DA-CREs) and observed a higher number of DA-CREs in glia than those found in neurons (Supplementary Fig. 4G, top panel). In addition, astrocytes contain the most significant number of DA-CREs, consistent with our observations of dynamic CREs and further supporting that astrocytes are enriched in disease-associated epigenomic dysregulation (Supplementary Fig. 4G, bottom panel).

Neurodegenerative diseases exhibit a stepwise progression of pathology from one brain region to another. This progression is accompanied by distinct epigenetic and molecular alterations that vary across specific cell types. Investigating chromatin remodeling across different brain regions, particularly those exhibiting differential tau pathology and neurodegeneration, will provide critical insights into the underlying mechanisms driving the progression of these diseases. In our analysis, both in the original and downsampled datasets, we observed a transition trend of gene deactivation in step with higher pathology when comparing the less affected brain region (PreCG) to the more affected brain region (insula), with PiD showing the apparent pattern of down-regulated DARs increasing in astrocytes, inhibitory neurons, and oligodendrocytes. PSP also exhibited prominent gene deactivation in astrocytes and inhibitory neurons (Fig. 2L and Supplementary Fig. 4H). These patterns were robust to donor variation and were reproduced using a mixed-effects differential testing framework that included donor as a random effect (Supplementary Fig. 6). We next examined the functional effects of the genes linked to DA-CREs shared between two regions, which were up-regulated in the PreCG but down-regulated in the insula. In PiD, the deactivated genes in astrocytes were primarily associated with cellular survival and inflammation response, while in oligodendrocytes, they were linked to immune regulation, cell migration, and apoptosis (Fig. 2M). In PSP, astrocyte-deactivated genes were involved in blood-brain barrier functions (Supplementary Fig. 6). Notably, CRE deactivation in PiD inhibitory neurons was enriched for genes regulating synaptic plasticity (Fig. 2M, N). A similar trend was observed in PSP, where deactivated genes in inhibitory neurons were linked to long-term synaptic depression (Supplementary Fig. 6), suggesting a common disruption of synaptic regulation across primary tauopathies. Altogether, the progressive loss of chromatin accessibility in advanced disease stages underlies the functional impairments observed in glia and neurons and may contribute to the onset of a neuroinflammatory response and imbalances in excitatory/inhibitory signaling.

### Disease dynamic peaks improve the explanation of disease genetic variants

More than 90% of GWAS-identified trait-associated variants are non-coding and likely influence gene regulation through changes in chromatin or CREs^47–50^. While it is known that cell type-specific gene regulatory elements differentially capture heritability factors, it remains unknown whether gene regulatory elements dynamically regulated in disease tissues also differentially capture heritability and if this occurs in a disorder-specific fashion. Therefore, we interrogated whether dynamic peaks in response to disease conditions would capture and interpret heritability differentially from condition-stable cell type-specific peaks. Integrating with GWAS studies of AD^51^, Frontotemporal dementia (FTD)^52^, and PSP^53^, we applied stratified LD score regression (S-LDSC) to partition the disease heritability and used two metrics enrichment and standardized effect size (${\tau }_{c}^{*}$) to evaluate the results (Methods). Heritability enrichment is the per-SNP heritability in an annotation divided by the overall per-SNP heritability. ${\tau }_{c}^{*}$ is the per-SNP heritability correcting for the annotation size and overall heritability.

**Fig. 3:**
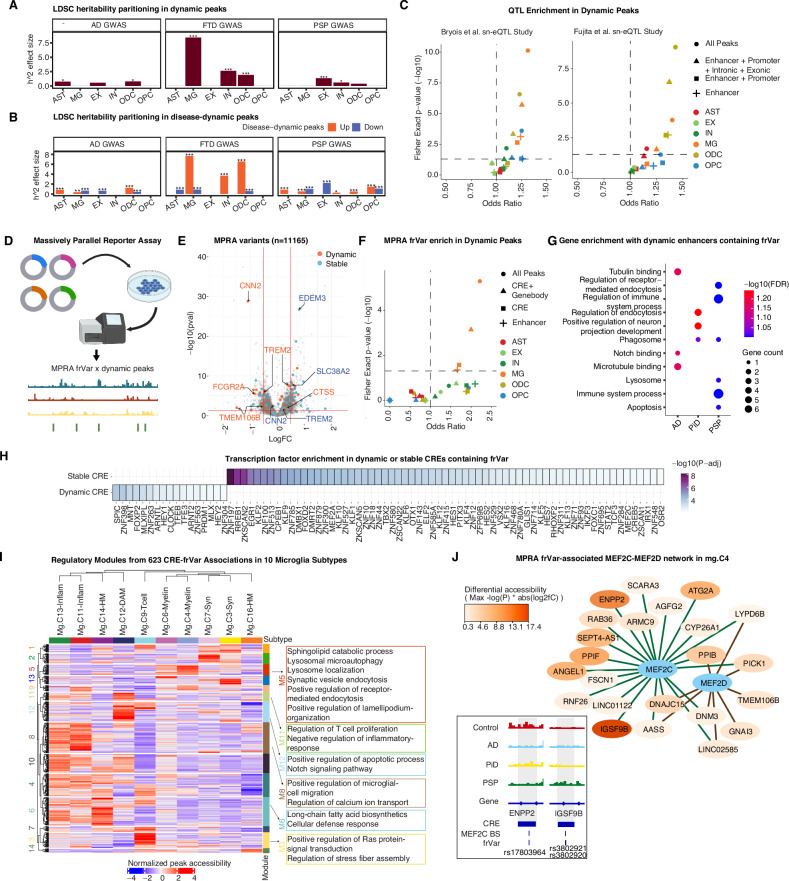
Dynamic accessible regions implicate disease heritability through GWAS, MPRA and sn-eQTL analysis. **A** Partitioned disease heritability in dynamic and stable peaks across cell types for AD, FTD, and PSP GWAS, represented by LDSC standardized effect size (${\tau }^{*}$). ‘FTD’ refers to the GWAS from^52^, representing a meta-analysis of five FTD subtypes. In this study, ‘PiD’ refers specifically to Pick’s disease samples analyzed in this dataset. **B** Partitioned disease heritability in dynamic peaks stratified by up- and down-regulated peaks for each disease within each cell type. For example, the PSP track depicts LDSC ${\tau }^{*}$ for PSP GWAS in PSP up- or down-regulated peaks. **C**, **F** Enrichment of sn-eQTLs (**C**) and MPRA-derived functional regulatory variants (frVars) (**F**) in dynamic versus stable peaks across cell types tested using two-sided Fisher’s exact tests. The y-axis shows −log10 nominal *P* values, and the x-axis shows the odds ratio. **D** Schematic overview of the MPRA experiment and integration with dynamic peaks. Created in BioRender. Han, X. (https://BioRender.com/7adtg7s). **E** Volcano plot of MPRA-tested variants showing log2 fold change versus −log10 nominal *P* values. Variants overlapping candidate CREs are annotated with representative genes. P values were obtained using the MPRAnalyze statistical framework. **G** Gene enrichment for dynamic enhancers containing MPRA frVars analyzed using ShinyGO 0.80. **H** TF enrichment of peaks containing MPRA frVars identified by MEME. **I** Heatmap showing regulatory modules of CREs with frVars across microglial subtypes. Peak accessibility in pseudobulked samples was log2-transformed after depth normalization, and the mean values of subclusters were quantile-normalized. **J** *MEF2C*-*MEF2D* regulatory network defining the mg.C4-specific frVar defining module (Module 5). Nodes represent frVar-overlapping CREs containing predicted MEF2C or MEF2D binding sites. CREs are colored by their maximal differential accessibility score, calculated as -log(*P* value) x |Log2FC| from marker peak calling. Green edges indicate *MEF2C* targets and brown edges indicate *MEF2D* targets. Multiple testing correction was performed using the Benjamini–Hochberg false discovery rate (FDR). LDSC ${\tau }^{*}$ with FDR thresholds: *<0.05; ** <0.005; *** <0.001. Source data are provided as a Source Data file.

While individual genetic variants from GWAS studies of AD, FTD, and PSP show similar distribution patterns across both consensus peaks and dynamic peaks (Supplementary Fig. 7A), combined heritability partitioning across different peak sets revealed notable differences between the GWASs. Different cell types captured the greatest amount of heritability of different diseases over disease-dynamic peaks. At the total cell type level, heritability enrichment was most significant across dynamic peaks observed in microglia, inhibitory neurons, and oligodendrocytes in PiD; oligodendrocytes and astrocytes in AD; and neurons for PSP (Fig. 3A). Notably, FTD heritability was most strongly captured by accessible regions whose chromatin accessibility changed in microglia in PiD. This included both gained chromatin accessibility and lost accessibility in a disease context-specific fashion. Peaks that gained chromatin accessibility in PiD microglia specifically showed the greatest overall FTD GWAS heritability score (Fig. 3B). In AD and PSP, significant changes in heritability-associated accessibility occurred in astrocytes. In PSP, peaks that lost accessibility in excitatory neurons showed the greatest overall heritability, which is consistent with the previous finding that GWAS enrichment is lost in subcortical projecting neurons in brain regions where they are selectively depleted^19^. These findings suggested that disease heritability is influenced by gene regulatory elements that are dynamic in disease-relevant tissue contexts, characterized by the loss of regulatory elements in both neurons and glia during degeneration, and a predominant gain in glia. These alterations differentially affect various cell types in a disease context-specific fashion that further relates to differential cellular vulnerability.

To test for the reproducibility in independent studies of enrichment for heritability among cell type-specific and disease-dynamic chromatin accessibility peaks, we analyzed snATAC-seq data from the Seattle Alzheimer’s Disease Brain Cell Atlas (SEA-AD) for the middle temporal gyrus (MTG) (Methods)^54^. In the SEA-AD replication analysis, we utilized published cell subclass annotations and the consensus peak set defined by SEA-AD^54^ to independently classify cells into six major types. For each cell type, we identified peaks that either changed across the four SEA-AD pathology stages (no AD, low, moderate, high) or remained stable. LDSC analysis was then conducted using only the SEA-AD peak sets. We found significant enrichment of AD heritability in microglial peaks showing disease-dynamic accessibility (Supplementary Fig. 7B), with marker peaks for each pathology stage consistently enriched for AD-associated variants. These findings replicate our original results with an entirely independent dataset and analytical pipeline, confirming that disease-dynamic chromatin accessibility plays a disproportionate role in GWAS heritability.

### MPRA constructs replicate in vivo microglia regulatory state

Massively parallel reporter assays (MPRAs) are a powerful functional genomics tool enabling high-throughput experimental assessment of the regulatory activity of non-coding DNA elements. To further evaluate the role of dynamic peaks in genetic regulation, we integrated CREs with MPRA generated on human microglial-relevant genetic variants in human microglial cell lines HMC3, with 11,167 verified variants in total (Fig. 3D, Methods)^55^. We first compared their distribution across dynamic peaks, stable peaks, and the full set of variants, finding that the distributions were comparable (Supplementary Fig. 8A). We then identified 3,325 functional variants (frVars) by *p*-value < 0.05, with the highest concentration found in microglia enhancers (Supplementary Fig. 8B). Among these, 310 were located in cell type-specific peaks (Fig. 3E). Notably, several neurodegeneration-associated genes, such as *TREM2* and immune-related *CNN2*, were linked to distinct CREs in different cell types in a disease-specific manner. This highlights the importance of context and disease-specific data in identifying candidate functional regulatory elements.

To assess TF relevance, we compared TF expression in HMC3 bulk RNA-seq (GSE219208) with snATAC-derived human microglia. We observed that 77.7% of HMC3 TFs overlapped with ~63.8% of TFs expressed in human microglia (Supplementary Fig. 9A), including canonical lineage and regulatory TFs such as *ERG1*, *SP1*, and *MYC* (Supplementary Fig. 9B). The strong positive correlation observed in scaled expression levels across shared TFs in mg.C4 and HMC3 indicates that HMC3 cells retain core microglial transcriptional features, including identity regulators (*NFIB*, *KLF6*, *FOXP1*), chromatin accessibility TFs (*CTCF*, *MAX*, *MYC*, *ZBTB18*), and immune signaling TFs (*ERG1*, *STAT2*, *RBPJ*). Although cell lines may lack disease-context-specific regulators, the conserved TF architecture supports the use of HMC3 as a relevant model for testing microglia-enriched regulatory mechanisms in MPRA.

To validate MPRA constructs using chromatin histone modification marks, we first re-annotated our identified enhancers by incorporating human prefrontal cortex (PFC) H3K27ac ChIP-seq data along with the consensus human cCRE reference from ENCODE (Supplementary Fig. 4B, Methods). Using this consensus CRE reference, we found that 42.8% of frVars overlapped with active CREs, predominantly distal enhancers (dELS; 28.8%), whereas less than 1% of non-frVars overlapped (Supplementary Fig. 9C), demonstrating strong specificity and indicating that the functional variants tested in MPRA reside in bona fide enhancer contexts.

To further evaluate cell-type-specific enhancer activity, we incorporated a single-nucleus CUT&Tag dataset capturing H3K27ac and H3K4me1 from AD and control brains (syn53191971). Approximately 68% of microglial CREs were supported by at least one histone modification mark (Supplementary Fig. 10A), confirming that the MPRA-tested elements overlap active enhancer states in vivo. In disease-associated states, such as mg.C4, we also identified disease-associated CREs based on both chromatin accessibility and histone modification changes (Supplementary Fig. 10B). The most upregulated genes in these microglia in disease were linked to ion homeostasis (*SLC24A3*, *LAT2*), lysosomal and protease activity (*CST7*, *CTSC*), and stress-related signaling (*DUSP5*, *BRF2*), consistent with the lysosome-enriched, stress-responsive profile of mg.C4.

Together, these analyses demonstrate that our MPRA constructs overlap validated enhancer states, exhibit disease-relevant activity, and are supported by TF expression profiles that recapitulate core microglial regulatory programs. These findings support the use of HMC3-based MPRA as an appropriate system for evaluating functional microglial regulatory variants, including those active in disease-associated states such as mg.C4.

### Enrichment of MPRA-validated functional variants within dynamic peaks

To understand the functional implications of these variants, including their upstream gene regulators and pathways, we conducted transcription factor analysis for dynamic vs. stable peaks containing frVars across all cell types (Fig. 3H). Our analysis revealed distinct patterns of TF enrichment. In dynamic peaks associated with frVars, the TFs identified were predominantly involved in metabolic regulation (*MLXIPL*, *MNT*) and cellular stress responses (*TFEB*, *TFE3*). Additionally, *SPIC*, known for its role in macrophage inflammation and regulation^56^, was also enriched. These TFs play crucial roles in managing cellular responses and maintaining homeostasis. In contrast, TFs enriched in stable peaks containing frVars spanned a broader range of biological processes, including developmental processes (*FOXD2*, *DMRT2*), cell differentiation (*MEF2A*, *KLF1*), immune responses (*STAT2*, *IKZF3*), and transcriptional regulation (*ZNF197*, *ZKSCAN2*).

We then investigated whether the peaks identified as dynamic in the context of human brain disease are enriched for experimentally validated functional variants, in comparison to peaks that remain stable in disease. We assessed the enrichment of frVars in dynamic versus stable peaks using Fisher’s exact test across peak types within each cell type. Notably, dynamic peaks in microglia showed significant enrichment of frVars, across all peak types (enhancers, CREs, CREs plus gene bodies, or all peaks) (Fig. 3F), suggesting that functional variants may modulate gene regulation by perturbing dynamic chromatin regions. To identify which genes were affected in each disease, we performed a gene ontology analysis of genes linked to dynamic CREs containing frVars. Both PiD and PSP were enriched for phagosome-related genes, and PSP-dysregulated genes (*FCGR2A*, *CTSS*) were also involved in lysosome and immune regulation. In contrast, dynamic CREs in AD with frVars were significantly enriched in genes related to microtubule binding (Fig. 3G).

To understand the functional implications of these variants in microglia states, including their upstream gene regulators and downstream pathways, we extracted CREs harboring frVar and partitioned them into co-accessible regulatory modules across microglia subtypes using hierarchical clustering based on normalized chromatin accessibility (Methods). These CRE modules exhibited subtype-specific activity and were enriched for distinct biological functions (Fig. 3I). Importantly, module 5, which was specifically activated in mg.C4, was involved in lysosome function, sphingolipid catabolic process, and synaptic vesicle endocytosis (Fig. 3I) and driven by transcription factor *MEF2C* (Fig. 3J). CREs within the mg.C4-specific module harbored *MEF2C* binding sites associated with frVar and were linked to genes enriched in endocytosis and pathways of neurodegeneration in multiple diseases (Fig. 3J). For example, a frVar rs17803964 overlapped a *MEF2C* binding site within a CRE of *ENPP2*, which is involved in the metabolic disturbances in AD^57,58^. This particular CRE displayed decreased chromatin accessibility in diseases compared to control, while others including in the gene *IGSF9B* showed increased accessibility. These results highlight coordinated context dependent gene regulation in microglia, identified high quality TF drivers based on high resolution mapping to frVars at nucleotide resolution, and support roles for human genetic variation in more broadly modulating microglial response to stressors in the human diseased brain.

### Enrichment of single-nucleus QTLs in dynamic chromatin

Single-cell resolution quantitative trait loci (QTL) have emerged as a valuable resource to explore the cell type-specific mechanisms that genetic variation influences gene expression in a context-dependent manner. Dynamic chromatin accessible peaks that harbor regulatory variants would support the peaks as functional regulatory elements and reveal various transcriptional activities under specific contexts. To further assess whether disease context-specific dynamic peaks in cross-disorder brain datasets are enriched for regulatory variants identified by large datasets sampling common genetic variation, we leveraged two single-nucleus expression quantitative trait loci (sn-eQTL) datasets from the human brain^59,60^ and performed QTL enrichment (Methods). We assessed the enrichment of eQTLs in dynamic versus stable peaks by Fisher’s exact test across different peak types in each cell type. Notably, across different peak categories and cell types, dynamic peaks in microglia and oligodendrocytes consistently exhibited significant eQTL enrichment, particularly within CREs plus gene bodies and across all peaks (Fig. 3C), suggesting that microglia and oligodendrocytes may serve as common targets in tauopathies, where common regulatory variants could modulate gene regulation by perturbing dynamic chromatin regions.

Using CREs harboring eQTLs, we reproducibly identified a mg.C4-specific regulatory module (Supplementary Fig. 8C). Importantly, eQTL module 10, which was specifically activated in mg.C4, was associated with lysosomal membrane function and protein kinase binding. TF analysis over linked enhancers identified nine key regulators, including *EGR1* and *PRDM9*, that drive the activation of module 10, targeting CREs linked to genes enriched in vesicle-mediated transport, lysosomal membrane, and cytoskeleton organization (Supplementary Figs. 8D and 11). For example, the eQTL rs12914843 of *FAN1* (P = 2.26 × 10⁻⁹) overlapped with a CRE of *FAN*1, potentially driving the observed increase in CRE accessibility in PSP and PiD. These findings highlight a potential lysosome-related dysregulation in disease-associated mg.C4. Through the combined eQTL and MPRA-based fVar annotation, we identified a series of candidate TF and target genes poised to regulate microglial lysosomal function and its variation in the human brain. These findings also support roles for human genetic variation in affecting key stress response pathways in glial that are significantly dysregulated in tauopathies.

We compared modules generated from frVar with those derived from microglia sn-eQTLs defined from AD and control tissues^59^ (Supplementary Fig. 12; Methods). Over 80% of the frVar modules aligned with sn-eQTL modules, including 12 of 14 frVar modules that corresponded directly to an eQTL module, and 9 specifying the same microglial states. Such correspondence demonstrates that these MPRA-defined variants capture representative, reproducible, and biologically meaningful regulatory programs rather than assay-specific effects. Together, this convergence strengthens the interpretation that frVar captures the functional axis along which genetic risk influences microglial behavior.

### Convergent gene regulatory modules regulated by MPRA frVar and eQTLs

To deepen our understanding of functional variants converging within gene regulatory modules, we extended our analysis by applying topic modeling to CREs containing either validated frVar or observed sn-eQTLs in microglia (Methods). This method helped us determine whether the regulatory structure observed in the overlap analysis reflects coordinated biological programs or simply results from shared genomic location or CRE abundance. Topic modeling revealed nine microglial regulatory programs, each enriched for specific TF motifs and involved in disease-related microglial states (Supplementary Fig. 13). For instance, the PiD-associated mg.C4 program was enriched in pathways linked to stress activation and lysosomal-myelin interactions (Supplementary Fig. 13C, D) driven by *JUNB* and *SPI1*, along with resilience-related pathways involving phagocytic recognition, metabolic homeostasis, and myelin maintenance driven by *MEF2C* and *RHOXF2* (Supplementary Figs. 14A, B). This suggests that PiD-related mg.C4 contains functional variants capable of influencing both stress-response pathways relevant to PiD pathology and supporting resilience. Importantly, the *MEF2C*-*MEF2D* centered regulatory network, which was previously identified through independent analyses of MPRA frVars, is recapitulated in this topic-modeling framework (Supplementary Figs. 14D), further supporting the robustness of this regulatory architecture and implicating *MEF2C* in mg.C4-associated regulatory program. Overall, this analysis reinforces the mechanistic understanding that functional and expression-modifying variants converge within dynamic CRE networks active in microglia in the context of human brain tissue, including across a disease spectrum, tuning TF-regulated pathways and directing microglial state changes specifically engaged in disease contexts.

To further evaluate whether these functional regulatory programs capture disease-relevant genetic signals in aggregate that may be underpowered or missed at the individual level in GWAS, we next examined *p* value distributions of variants across GWAS and functional datasets. Approximately 13% of the functional variants within these topics fall below MAF 0.05 reflective relative rarity compared to GWAS significant hits. Detection of low-frequency variants under a genome-wide significance threshold (α = 5 × 10⁻⁸) with >80% power, assuming a modest genotype relative risk (OR = 1.1), requires cohorts on the order of ≥100,000 total individuals (GAS power calculator^61^). In contrast, current FTD and PSP GWAS remain substantially smaller (N_FTD = 12,928, N_PSP = 8,363), limiting power to identify modest-effect regulatory associations. In the PiD mg.C4, we found 16 variants converging in topic 9 that exhibit functional regulatory activity yet fall below genome-wide significance thresholds (p < 5×10⁻⁸) in AD GWAS (Supplementary Fig. 14C). Notably, several variants lie near known AD risk genes such as *TREML4*, *RIN3*, *ALCAM*, and *CD300A*, as well as candidate genes such as *SLC25A37*, *STK10*, and *MARK3*. *MARK3* encodes a kinase that phosphorylates tau and may affect tangle formation; *SLC25A37* encodes a mitochondrial iron transporter associated with iron dysregulation; and *STK10* encodes a kinase involved in T-cell signaling and inflammation, although less studied in the brain. Furthermore, *RABEP1*, which encodes an endosomal trafficking protein crucial for lysosomal pathways, APP processing, and microglial phagocytosis, showed significance in both GWAS and functional assays, highlighting its role as a potential convergent disease node. These results indicate that functional regulatory variants, validated through MPRA and supported by sn-eQTL and chromatin accessibility, capture disease-relevant biology even when they fall below GWAS significance thresholds.

Together, these analyses demonstrate that microglia eQTLs, including experimentally validated frVars, converge on biologically coherent and disease-relevant microglial programs, and that functional variants within these programs highlight regulatory mechanisms not fully captured by GWAS alone. This supports a model in which human functional genetic variants modulate microglial responsiveness by tuning TF-governed pathways that are selectively engaged across tauopathies.

### Diversity of cellular subclusters across brain regions

**Fig. 4:**
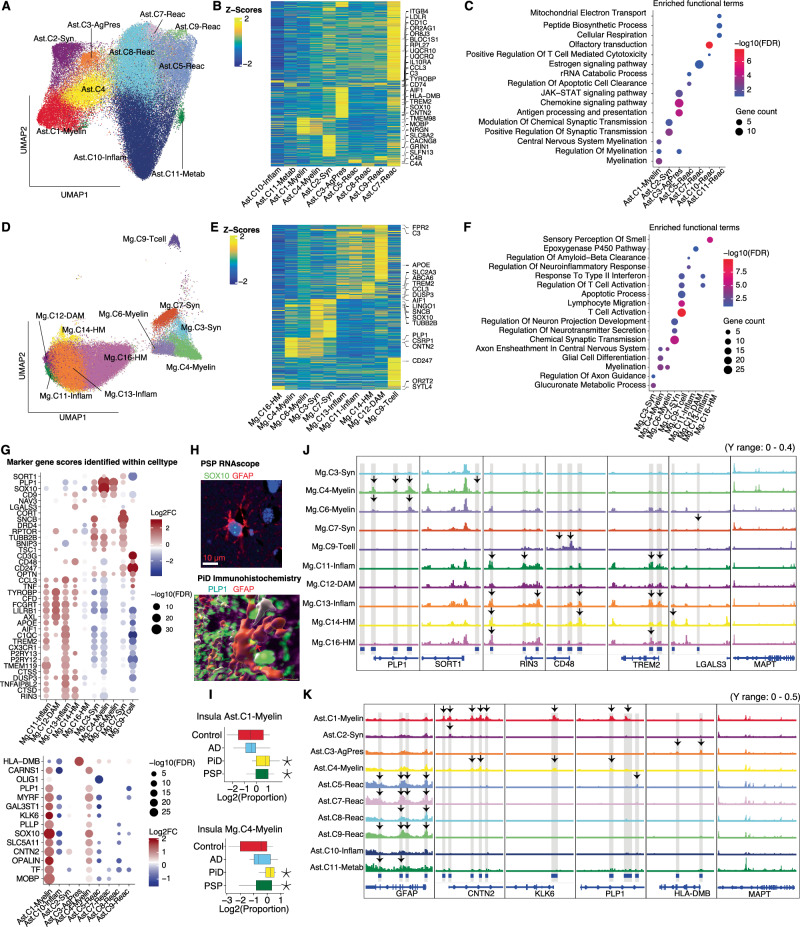
Diversity and heterogeneity of cellular subtypes in human brain of tauopathies. **A**–**C** UMAP embedding of subclusters of astrocytes (**A**), with a heatmap of gene score matrix labeled by Log2fc > 1 for marker gene scores (**B**) and enriched functional terms associated with these marker genes (**C**). ASC, astrocytes. **D**–**F** Similar analyses for microglia subclusters. MG, microglia. **G** Gene signatures of astrocyte subclusters (top) and microglia subclusters (bottom), defined by marker gene scores compared within each respective cell type. **H** RNAscope ISH for *SOX10* combined with GFAP IHC in human insular tissue from a PSP patient. Representative images show co-localization of *SOX10* mRNA and GFAP in a subset of astrocytes (Scale bar: 10 µm). **I** Boxplots displaying the relative abundance of insula ast.C1 (top) and mg.C4 (bottom) across conditions. Changes in cell composition between disease and control groups were tested using two-sided linear models implemented in the limma package, adjusted for age and post-mortem interval. These analyses showed significant increases in ast.C1 abundance in PiD (*P* = 0.02) and PSP (*P* = 0.011), and in mg.C4 abundance in PiD (*P* = 0.016) and PSP (*P* = 0.045). Data points represent individual donors for Control (*n* = 8), AD (*n* = 10), PiD (*n* = 8), and PSP (*n* = 11). The center line indicates the median; boxes represent the interquartile range (IQR); whiskers extend to 1.5×IQR. Source data are provided as a Source Data file. **J** Example tracks showing marker peaks for genes *PLP1*, *SORT1* in mg.C4; *RIN3* and *TREM2* in mg.C13 and mg.C6; CD48 in mg.C9 and mg.C6; and *LGALS3* in mg.C7 and mg.C14. **K** Example tracks showing marker peaks for genes *CNTN2*, *KLK6*, and *PLP1* in ast.C1; *HLA-DMB* in ast.C3; and GFAP in multiple astrocyte subclusters.

Having identified dynamic peaks including functional variants that link to risk genetic factors, we next sought to understand their organization across distinct cell subclusters representing coordinated cellular biological responses to tauopathies. For this, we used the 50 total subclusters that were achieved by combining all cells from a given major cell type across brain regions and disorder for high-resolution cellular subtyping, which we accomplished with negligible batch effect and donor effect due to our cross-disorder mixed library preparations (Supplementary Figs. 15, 16, and Methods). On average, over 80% of donors within each diagnostic group contributed at least 1% of cells to each subcluster, indicating balanced donor representation. These subclusters comprised 10 astrocytes, 10 microglia, 12 neurons, 8 oligodendrocytes, and 10 oligodendrocyte precursor cells (OPC) (Fig. 4A–F, Supplementary Figs. 15 and 17, and Methods). Consistent with previous studies^10,12,15,62–64^, we reproduced astrocyte subclusters associated with homeostasis, reactive states (ast.C5, ast.C7, ast.C8, ast.C9), synaptic transmission (ast.C2), antigen presentation (ast.C3), inflammation (ast.C10), and metabolism (ast.C11), as well as microglia subclusters relate to homeostatic (mg.C16, mg.C14); inflammatory (mg.C13, mg.C11); disease-associated microglia (DAM) (mg.C12), synaptic transmission (mg.C4, mg.C7) and T cell activation (mg.C9) (Methods). Additionally, we identified two myelin-related astrocyte subclusters (ast.C1, ast.C4) and microglia subclusters (mg.C4, mg.C6) expressing *PLP1* and *SOX10* (Fig. 4G, and Supplementary Figs. 17B, C), extending the findings of prior studies. The tau-encoding gene *MAPT* was upregulated in mg.C4 (Fig. 4J and Supplementary Fig. 18A, left panel) and differentially expressed in PSP in insula in ast.C1 (Supplementary Fig. 18A, right panel), suggesting that these two subclusters may be associated with tau dysregulation.

Chromatin tracks revealed that marker peaks of marker genes were consistently up-regulated in distinct subclusters, such as *GFAP* in reactive astrocytes; *HLA-DMB* in ast.C3 (Fig. 4K); *RIN3* in mg.C13, *CD48* in mg.C9, *TREM2* in mg.C13 and mg.C11, and *LGALS3* in mg.C7 and mg.C14 (Fig. 4J). Both ast.C1 and ast.C4 were myelination-related and oligodendrocyte-like, with ast.C1 displaying prominent marker peaks for *KLK6* and *PLP1* (Fig. 4K). Compared to other astrocyte subclusters, ast.C1 highly expressed oligodendrocyte markers, including *MOBP*, *TF*, and *OPALIN*, as well as disease signature genes found in oligodendrocytes, such as *CNTN2*, *SLC5A11* (Fig. 4G). Similarly, *PLP1*, a X-chromosome gene that participates in age-related resilience^65^, was most highly upregulated in mg.C4, while mg.C6 exhibited greater chromatin accessibility around *RIN3*, *CD48*, and *TREM2* (Fig. 4J).

For neurons, we identified four inhibitory neurons and eight excitatory neurons (Supplementary Fig. 17B). The four inhibitory neurons consist of four *SLC32A1*^+^ *GAD1*^+^ cells. Among them, three expressed additional markers: *PVALB*^+^ (neu.C7), *SST*^+^ (neu.C8) and *ADARB2* ^+^ *VIP* ^+^ (neu.C6). The four excitatory neuron subclusters expressed layer-specific markers: neu.C5 and neu.C11 were *FEZF2*^+^, neu.C14 was *RORB*^+^, and neu.C13 was *CUX2*^+^.

To contextualize our findings with prior published work, we compared these clusters with previously defined microglia states at the RNA level within this same dataset^19^ to identify overlapping and similar states (Supplementary Fig. 18B). ATAC data provided higher-resolution partitioning of cells compared to RNA data in several instances, notably with mg.C4 and mg.C6, two distinct ATAC-defined clusters that both overlap with the RNA-defined insula-microglia-3 cluster. These clusters resemble myelin-eating microglia observed in multiple sclerosis (MS) datasets^64^, all of which share *PLP1* as a marker gene. While both mg.C4 and mg.C6 share markers of myelination, they differ predominantly in the expression of lipid transporters (*ABCA2*, *ABCA8*, *SCAP*) and lipid metabolism genes (*HMGCS2*, *LPGAT1*), and genes associated with buffering metabolic stress (*SGK2*, *TSC1*). Comparing mg.C4 with the microglia subtypes described in ref. ^64^, we found significant marker overlap with a myelin-phagocytosing microglia population observed in MS (Supplementary Fig. 19A and Methods). However, our cluster demonstrated increased chromatin accessibility at myelin regulatory and processing genes (Fig. 4G, J), with co-enrichment at their promoters of *SOX10*, suggesting active regulation in mg.C4. Furthermore, upon re-clustering our in-house snRNA-seq microglial cells into 10 clusters, we identified a microglial subtype expressing both *SOX10* and *PLP1*. This subtype shows a strong alignment with the ATAC subcluster mg.C4 (Supplementary Figs. 19B, C), further supporting that the mg.C4 is putative myelination-related microglia.

To illustrate how disease-context chromatin remodeling engages specific glial populations, we identified cell type-specific CREs that were differentially activated in disease contexts. For instance, in mg.C14, we found a CRE of *GRN* that was uniquely open in PiD (Supplementary Fig. 20A). This CRE included binding motifs for *FOS* and *CEBPB*, key drivers of mg.C14. *FOS* motifs, known to be enriched in stress and immune responses, especially under Aβ stimulation^66^, while *CEBPD* is a significant regulator of inflammatory signaling^67^. Activation of this enhancer likely reflects an immune activation-induced regulatory circuit through which PiD microglia elevate *GRN* expression. Conversely, in PSP, we identified a *TREM2* enhancer selectively downregulated in *TREM2*⁺ mg.C13 (Supplementary Fig. 20B). This CRE contained motifs for *SPI1* and *BCL11A*, both enriched in mg.C13. SPI1, an established AD risk factor, controls microglial identity and survival^44^, whereas *BCL11A* acts as a chromatin regulator. The downregulation of this *TREM2* CRE in PSP suggests a regulatory mechanism where decreased *SPI1/BCL11A* activity may reduce *TREM2* expression, weakening microglial resilience in PSP. These findings illustrate how distinct disorders engage disease-specific CRE-TF-gene circuits in specific glial populations.

### Variable disease-enriched glial states by disorder

We next explored how cellular composition changes in disease within each brain region. Changes in cell proportions by cell type, disease, and brain region were summarized in Supplementary Data 2 and Supplementary Figs. 21–22, with notable examples shown in Fig. 4I. In the insula, we observed a proportional increase in total microglia in AD and in astrocytes in PiD (Supplementary Fig. 21B). At the subcluster level, we identified disease-specific and common subcluster composition changes in a region-specific manner (Supplementary Fig. 22).

We noticed prominent changes in PiD among oligodendrocytes and myelin-related subtypes. PiD exhibited the most pronounced disease-specific changes in subtype composition in inflammatory oligodendrocyte subclusters within the insula, characterized by a decrease in odc.C7 and odc.C8 and an increase in odc.C9. Notably, alterations in myelin-related *PLP1+* subclusters were shared by diseases in the insula but involved different glial types depending on the disorder. Both ast.C1 and mg.C4 were significantly increased in PiD and PSP (Fig. 4I), while mg.C6 was elevated in both PiD and AD. At the same time, Ast.C1, mg.C4, and mg.C6 express their expected markers of astrocytes and microglia, such as *GFAP* and *C3* (Supplementary Fig. 17B). The increase in ast.C1 and mg.C4 in PSP and PiD was also supported by bootstrapped analysis (Supplementary Fig. 18C and Methods). These combined observations of increased accessibility of genes associated with myelination, including *PLP1*, which has recently been shown to be neuroprotective^65^, in microglia and astrocytes, suggest a shared feature of primary and secondary tauopathies. These findings showed that the insula accumulated pathological signals with the myelin-related microglia and astrocytes increasing in PiD and PSP.

### TF drivers of disease-reactive microglia and astrocytes in PiD and PSP

Importantly, microglia containing *PLP1* mRNA have been reproducibly described^64^, and have been shown experimentally to occur secondary to myelin phagocytosis. Our findings suggested that *PLP1* chromatin accessibility varies within disease-associated reactive glia. We aimed to further investigate the gene regulatory mechanisms driving *PLP1* and other myelin-related genes in tauopathies and explore how cell stress and injury pathways interact with tau pathology.

We performed TF analysis and found the master regulator of myelination genes, *SOX10*, was significantly enriched in both mg.C4 and ast.C1 (Supplementary Data 3). *SOX10*, a transcription factor typically associated with oligodendrocytes but also with known roles in neuroprotection, was among the top markers distinguishing ast.C1 and mg.C4 from other astrocytes and microglia. Comparatively, *SOX10* exhibited its highest expression in oligodendrocytes, as expected (Supplementary Fig. 17B). To identify TF targets by mediating binding events in accessible chromatin, we predicted TF binding sites on the marker peaks of these subclusters (Methods). Importantly, while 34.4% of *SOX10* targets overlapped across oligodendrocytes, astrocytes, and microglia, including the myelin-associated genes *PLLP*, several targets were uniquely prominent among *SOX10* targets in the non-oligo cell types, including *PDGFA* and *RBPJ*, supporting shared and distinct effects of *SOX10* by cell type (Supplementary Data 4). In mg.C4, *SOX10* shared targets (such as *CHRAC1*, *API5*) with another enriched TF, *FOXP2*, which is associated with language development and linked to neuropsychiatric disorders, and has been shown to exhibit human-specific expression in microglia^68^. In contrast, in ast.C1, *SOX10* shared targets (such as *BEST1, CDH5*) with a different TF, *NFIX*, which is an astrocyte-specific TF with roles in astrocyte development and maturation levels (Supplementary Data 5).

To confirm TOBIAS’s predicted TF targets within subtype marker peaks, we compared these predicted binding sites with TF ChIP-Seq data from the Gene Transcription Regulation Database (GTRD)^69^, focusing on datasets for *SOX10*, *NFIX*, and *FOXP2* (Experiment IDs: EXP034107, EXP038549, EXP010606). Although these data come from non-brain cell lines, approximately 20% of the predicted TF targets overlapped with orthogonal ChIP-seq peaks in our myelin-related glia subtypes (Supplementary Fig. 23), offering partial external validation of these regulatory interactions.

To investigate the protective role of mg.C4 and its divergence from the disease-associated pathway, we employed Slingshot^70^ to infer pseudotime trajectories for microglia in the insula (Methods). This analysis included cells associated with PiD-linked mg.C4, DAM-linked mg.C12, and homeostatic mg.C16. Beginning from homeostatic mg.C16, we identified three main lineages: one progressing toward mg.C4, one toward mg.C12, and the third representing a mixture of all three subtypes (Supplementary Fig. 25). The mg.C4-directed trajectory showed gradual activation of autophagy-lysosome regulators (*PARK7*, *TFEB*, *NFE2L1*), lipid homeostasis (*ADAM10*), and myelination (*CNP*, *OLIG2*, *MOG*). In contrast, the DAM-directed lineage upregulated inflammatory mediators including *NRROS*, *PRDM1*, and classical DAM markers such as *GPNMB* (Supplementary Fig. 26). Together, these trajectories show that mg.C4 is a distinct, non-inflammatory microglial state that engages coordinated myelin-lysosome programs as an adaptive response to metabolic and disease stress.

Interestingly, *SOX10* target genes in mg.C4 and ast.C1 both enriched in process of cell population proliferation, developmental growth and lipid biosynthetic process (Supplementary Data 6). This suggests that these two clusters, driven by *SOX10*, may play a role in promoting cellular growth and lipid metabolism to maintain brain function. Importantly ast.C1 shows the highest gene activity and expression of *MAPT* compared to all other astrocytes in PSP (Supplementary Fig. 18A and Supplementary Data 7). We next identified TF target genes in each disorder by calculating gene-TF linkage scores within ast.C1 (Supplementary Fig. 24A, B and Methods). We found that *SOX10* was differentially activated in the PSP insula, targeting a SNARE protein, *STX4*, in all disease conditions (Supplementary Fig. 24A). In PSP, the predominant tau species retains exon 10, known as the 4 R isoform. SNARE genes mediating lysosomal membrane fusion affect tau propagation in iPSC models of 4 R tau^71^. Therefore, in ast.C1, *SOX10* participates in regulating both myelin genes and pathways previously implicated in the toxicity of 4 R tau. Consistently, epigenomic erosion analysis using single-nucleus methyl-3C sequencing data revealed significant gains and losses of heterochromatin across multiple astrocyte states in PSP, where ast.C1 remained unaffected (Supplementary Fig. 24C, Methods). The maintenance of epigenomic stability in ast.C1 may indicate relative protection of chromatin in ast.C1 compared to three other disease associated astrocytes (including ast.C10, and ast.C3, ast.C8).

### In vitro validation of disease-activated changes in glial gene regulation

*SOX10* expression is typically repressed in microglia and astrocytes relatively to oligodendrocytes, yet we observed low levels of ectopic expression of *SOX10* and downstream programs in multiple tauopathy associated glial states, including mg.C6, mg.C4 and ast.C1. To strengthen external validation of the observed expression of *SOX10* in subpopulations of microglia and astrocytes in disease, especially PiD and PSP, we performed complementary histological and imaging assays in independent human brain samples (Methods). RNAscope for *SOX10*, *GFAP* (astrocyte marker), and *C3* (microglia marker) in control and PSP insula tissue revealed a consistent increase in *SOX10*⁺ astrocytes and *SOX10*⁺ microglia across multiple sampled regions in PSP (Fig. 4H and Supplementary Fig. 28 A-E), supporting the presence of low levels of ectopic glial expression of *SOX10* in disease tissue. In parallel, immunohistochemistry for PLP1 (*SOX10* target) and GFAP (astrocyte marker) in control and PiD brains, combined with 3D confocal reconstruction and spot-distance analysis (Methods), demonstrated *PLP1*⁺ astrocytes tended to increase in PiD, showing close spatial association between *PLP1*⁺ structures within *GFAP*⁺ astrocytes in tauopathy samples (Fig. 4H and Supplementary Fig. 28F, G). Together, these orthogonal RNAscope and IHC validations support that *SOX10*⁺/*PLP1*⁺ myelin-related astrocyte and microglial states are present and enriched in human tauopathies, and a shared features of PiD and PSP.

To functionally test the role of *SOX10* in regulating microglial stress responses, we activated *SOX10* using CRISPRa in human iPSC-derived microglia and exposed these cells to PSP patient-derived synaptosomes, thereby modeling a primary tauopathy-related stress environment, followed by bulk RNA-seq profiling (Methods). In control microglia lacking CRISPRa induction, exposure to PSP synaptosomes induced robust upregulation of lysosome-associated genes linked to lysosomal stress and burden (Supplementary Fig. 29A). Exposure to synaptosomes also increases lysosome activity as measured by fluorescence imaging of active lysosomes in treated microglia. (Supplementary Fig. 29B, Methods). These findings are consistent with engagement and processing of diseased synaptic material.

Notably, CRISPRa drove limited *SOX10* expression in microglia cultured without synaptosomes. In synaptosome-treated microglia, CRISPRa induced stronger activation of *SOX10* expression and a markedly stronger transcriptional response, indicating that *SOX10* activity is selectively potentiated under stress conditions. This response closely mirrors the low-level *SOX10*-associated transcriptional programs observed in disease-enriched microglia and astrocytes in vivo, in contrast to the high *SOX10* expression characteristic of oligodendrocytes. Consistent with this stress-dependent effect, CRISPRa-*SOX10* microglia recapitulated transcriptional programs observed in human mg.C4 cells (Supplementary Fig. 29C), including coordinated induction of proliferation, cytokine signaling, and lysosomal-phagocytic pathways. Pathways enriched upon *SOX10* activation, such as myeloid leukocyte activation, Toll-like receptor signaling, and cell population proliferation (Supplementary Fig. 29D and Supplementary Data 15) closely mirrored the in vivo mg.C4 signature. Together, we validated that lysosomal stress induced by exposure to disease brain-derived synaptosomes increases the capacity of microglia to activate ectopic *SOX10* expression and its downstream gene programs. These findings support a model in which *SOX10* acts as a stress-responsive regulator that amplifies lysosomal-phagocytic and proliferative programs in microglia upon exposure to tau-associated synaptic material.

### Genetic heritability enriched in disorder-divergent cell states in PSP and PiD

Given emerging evidence that polygenic trait expression is most pronounced in disease-specific biological states related to causal pathways, we next employed heritability partitioning to assess relationships between gene regulation, enhancer co-accessibility, disease risk loci, and downstream pathways using diverse disease contexts. Therefore, we performed LDSC as previously described, applied to cell subclusters representing distinct states.

**Fig. 5:**
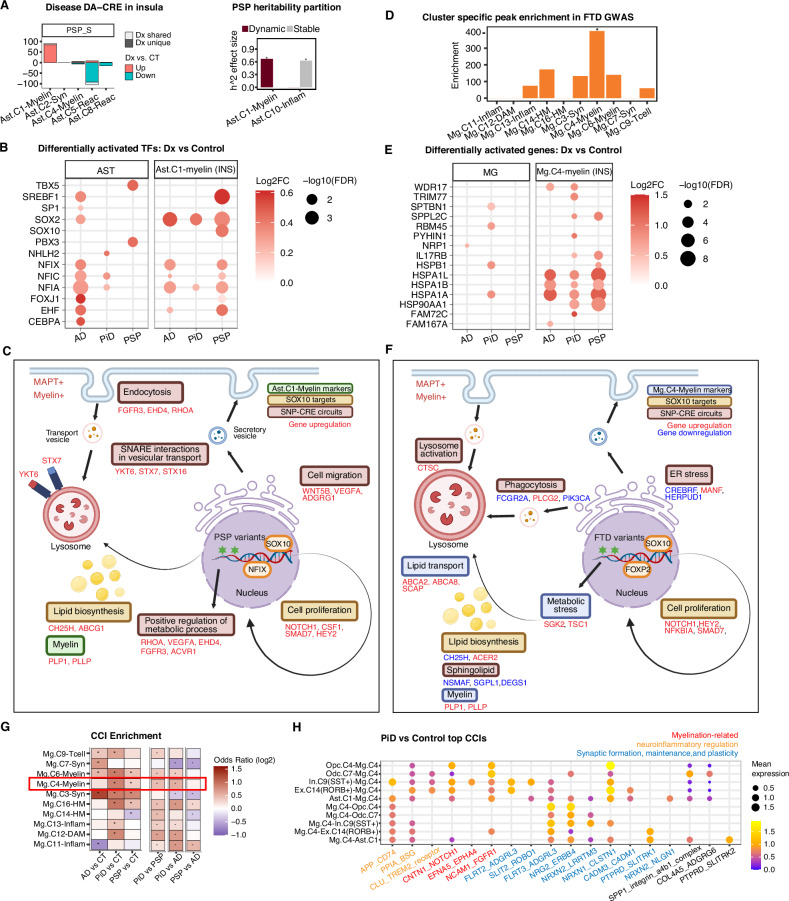
Integrated analysis of accessibility changes, gene activity, GWAS heritability partition, epigenomic stability, and cell-cell interactions identifies disease-associated glia subtypes. **A** PSP-associated chromatin accessibility changes in astrocytes. Bar plots (left) show the number of differentially accessible CREs in PSP across astrocyte subclusters, categorized by up- and down-regulation. The right panel shows partitioned disease heritability of dynamic peaks in ast.C1 and ast.C10 (right), displaying LDSC standardized effect size (${\tau }^{*}$). FDR * < 0.05; ** <0.005; *** <0.001. Source data are provided as a Source Data file. **B** Differentially activated TFs in astrocytes and ast.C1. **C** Schematic of molecular changes and dysregulated pathways driven by PSP GWAS risk variants in PSP ast.C1. Myelin-related astrocytes proliferate in tauopathy, activating SNARE-mediated vesicle trafficking and lysosomal pathway to mitigate lipid stress induced by PSP risk variants. Created in BioRender. Han, X. (https://BioRender.com/7adtg7s). **D** Disease heritability partition in subcluster-specific peaks for FTD GWAS across microglia subtypes. FDR * < 0.05; ** <0.005; *** <0.001. **E** Differentially activated genes in microglia and mg.C4. **F** Schematic of molecular changes and dysregulated pathways driven by FTD GWAS risk variants in PiD mg.C4. Myelin-related microglia proliferate in tauopathy, activating lysosome-phagocytosis pathways to counteract ER and metabolic stress induced by FTD risk variants. Genes with upregulation are shown in red font, and those with downregulation in blue, based on differential gene scores or genes linked to differentially accessible CREs. Created in BioRender. Han, X. (https://BioRender.com/7adtg7s). **G** Number of cell-cell interactions (CCIs) involving subclusters that are overrepresented in pairwise comparison. (*) FDR < 0.05; chi-square test. **H** Top ligand-receptor pairs mediating interactions between mg.C4 and the represented subclusters in PiD. Values represent the mean expression of ligand-receptor pairs in corresponding cellular pairs.

We applied LDSC analysis at the sub-cell-type level for all 50 clusters. Using FTD GWAS as input, we found two clusters (mg.C4, opc.C4) across all cell types were enriched for FTD heritability using LDSC enrichment (Fig. 5D and Supplementary Fig. 24D). Using PSP GWAS as input, LDSC enrichment in clusters did not pass the same *P* value significance threshold, while the trend matches to the LDSC ${\tau }^{*}$. The positive and significant ${\tau }^{*}$ implied that the PSP heritability enrichment effects were captured by five neuron subclusters (neu.C5, neu.C8, neu.C9, neu.C12, neu.C13), as previously seen at the major cell type level, and two astrocyte clusters (ast.C1, ast.C10) (Supplementary Fig. 24F). Importantly, the excitatory neurons demonstrated an overall loss of chromatin accessibility at PSP GWAS loci, while astrocytes in contrast exhibited a gain in accessibility at these loci (Fig. 3B).

Our goal was to identify cell states with the most pronounced aggregated co-regulatory structures affecting potential causal SNPs. We aimed to map their key regulators, targets, and downstream pathways and compare these features across disorders. At the sub-cell-type level, we found DA-CREs concentrated in glial subclusters across all three disorders and brain regions (Supplementary Fig. 24G). Ast.C1 showed the greatest number of disease-specific gained open chromatin accessibility in PSP samples in the insula (Fig. 5A, left panel). The significant enrichment of PSP heritability was found in both ast.C1-specific peaks and disease-dynamic peaks (Fig. 5A, right panel and Supplementary Fig. 24F), suggesting ast.C1 is a state harboring PSP heritability-relevant chromatin accessibility, which may contribute to PSP causality. *SOX10* was not only identified as a marker gene for ast.C1, but also exhibited differential activation in PSP (Fig. 5B). We then prioritized functional genes for ast.C1 based on their linked PSP differentially activated CREs associated with GWAS variants (Methods). Surprisingly, we found that three other SNARE-related genes, *STX7*, *YKT6*, and *STX16*, were activated and enriched in SNARE-mediated vesicular transport^72^ (Supplementary Fig. 27A and Supplementary Data 8). The SNARE protein *YKT6* is known to control the stress response and enhance lysosomal activity in Parkinson’s disease^73^. Notably, *STX7* and other SNARE-related genes, including a well-established PSP risk gene *STX6*^53^, were differentially upregulated in ast.C1, particularly in PSP (Supplementary Fig. 27B), supporting ast.C1 plays a role in vesicle trafficking. Moreover, other genes involved in the peak-to-gene regulatory circuitry were enriched in endocytosis, regulation of cell migration, synapse, and vesicle (Supplementary Data 9). These findings suggest that ast.C1 may enhance intracellular trafficking, including endocytosis and vesicle-mediated transport, in response to tau and myelin accumulation. This process may contribute to maintaining protein homeostasis and preventing cellular dysfunction, potentially mitigating neurodegenerative processes in PSP (Fig. 5C).

To validate our results of PSP-related ast.C1 with a more recent PSP GWAS^74^, we reran LDSC analysis on astrocyte subtype marker peaks (Log2fc > 0.5 and FDR < 0.1). We observed PSP heritability was only consistently enriched in ast.C1, confirming this subtype specifically captures genetic signals linked to PSP (Supplementary Fig. 24F). Although LDSC with the updated GWAS also detected ast.C3, an antigen-presenting astrocyte state, this subtype did not show significant differential accessibility in PSP across any region, suggesting PSP does not trigger an epigenomic response in these cells. Using the expanded variant set from the updated PSP GWAS, we reliably identified SNP-CRE circuits upregulated in PSP ast.C1 involved in SNARE interactions, endocytosis, and cell migration (Fig. 5C, in Supplementary Data 10-11). These non-coding regulatory circuits were additionally enriched for protein metabolic activation (Fig. 5C), indicating metabolic stress responses similar to those seen in PiD-associated mg.C4.

In microglia, we observed the highest gain of FTD heritability specifically in mg.C4 (Fig. 5D), implying mg.C4 is a PiD-associated subtype whose accessibility changes might capture FTD causality. Given that mg.C4 involved myelin genes, we tested multiple sclerosis (MS) GWAS as a comparison, but found MS heritability score maximal over a different cluster, mg.C11, marked by genes *TMEM119* and *TREM2* (Supplementary Fig. 24E). The known important FTD disease modifier genes affecting *TDP43* forms of the disease (*GRN*, *SORT1*) were more differentially expressed in mg.C4 than in any other microglia type (Supplementary Data 12)^75^. Interestingly, one CRE in mg.C4 contained an FTD SNP (rs10734151) targeting gene *CTSC*, a cathepsin protein which degrades proteins in a lysosome pH-dependent manner and was a candidate genome-wide significant hit reported in the original FTD GWAS study, but through a separate locus (rs74977128). We combined CRE with linked FTD GWAS-associated SNPs and performed pathway analysis to define downstream biology. Genes involved in immune system processes, sphingolipid signaling pathway, apoptosis, Fc gamma R-mediated phagocytosis, and regulation of response to endoplasmic reticulum stress were enriched among targeted genes (Supplementary Data 13 and 14). Genes differentially activated in PiD in mg.C4 include heat shock proteins and cytokine signaling, suggesting a potential role for regulating microglial pathways related stress and immune response (Fig. 5E). Taken together, mg.C4 might be subject to additional lipid regulation, which enhanced its ER stress response and activated the degradation pathways, including lysosome, phagocytosis, and apoptosis (Fig. 5F).

Additionally, cell-cell interactions (CCIs) inferred by CellPhoneDB based on imputed gene expression (Methods) showed higher CCIs involved in mg.C4 in PiD compared to the other three conditions (Fig. 5G), indicating increased cellular interaction activity in PiD. The cellular interactions orchestrate single-cell functions to maintain homeostasis and regulate physiological processes. We thus honed in on the interactions between mg.C4 and representative neuronal and glial subclusters, such as *RORB*^+^ excitatory neuron C14, the expanded *SST*^+^ inhibitory neuron C9, the largest oligodendrocyte subcluster odc.C7, and PSP-associated ast.C1 (Fig. 5H). We found key ligand-receptor pairs mediating signaling related to myelination, inflammation, and synaptic regulation, including PiD-risk genes *TREM2*, *CLSTN1*, and *PPIA*. *TREM2* plays a protective role in microglia and may promote myelin metabolism^76,77^. *NOTCH1*, previously identified in the PiD regulatory circuit (Supplementary Data 13), was found to mediate interactions between mg.C4 and neurons via the *CNTN1* ~ *NOTCH1* pair, highlighting its role in myelination and neuroinflammation. These results further underscore mg.C4 as a key subcluster responsive to PiD pathology.

## Discussion

### Major biological discoveries

Our study presents a comprehensive analysis of chromatin accessibility and transcriptional profiles of over 600,000 individual nuclei from the matched brain region samples of 41 individuals in tauopathies and controls. By jointly analyzing single-nucleus ATAC-seq and RNA-seq data, we identify cell type- and context-specific cis-regulatory elements and characterize chromatin accessibility changes using both pairwise disease-control and condition-wide comparisons. Leveraging disease-dynamic peaks, we interrogate GWAS variants, single-nucleus eQTLs, and MPRA-validated functional variants to define regulatory transcription factors, prioritize target genes, and uncover non-coding regulatory circuits disrupted in pathology-relevant cell states. Through this framework, we identify two disease-associated glial states, including PiD-related microglia mg.C4 and PSP-related astrocytes ast.C1, that are reproducibly expanded in the disease-affected insula and exhibit coordinated epigenomic, transcriptional, and functional signatures linked to disease context.

A central finding is that chromatin accessibility dynamics, rather than static cell-type identity, most effectively capture disease heritability and functional genetic variation. We observe a conserved transition in chromatin accessibility from moderately to severely affected brain regions, reflecting coordinated regulatory activation and repression during disease progression. Disease-dynamic cis-regulatory elements are disproportionately enriched for GWAS variants, single-nucleus eQTLs, and MPRA-validated functional variants; in particular, FTD heritability is most strongly explained by dynamic accessibility changes in microglia in our dataset. These dynamic CREs converge into transcription factor-centered regulatory modules, with *MEF2C* and *MEF2D* emerging as a key regulatory hub linking common genetic variation to lysosomal, phagocytic, and stress-responsive programs in disease-associated microglia.

Glial expansion represents a conserved response to neurodegeneration, often linked to immune activation and clearance of pathological material^77–80^. In tauopathies, this response resolves into distinct, genetically informed glial states rather than a uniform reactive program. In PiD, expansion of the myelin-associated microglial state mg.C4 concentrates FTD heritability within dynamic regulatory elements that converge on lysosomal and vesicle-mediated stress programs, consistent with a role in lipid and debris clearance. In contrast, PSP is characterized by expansion of a myelin-related astrocyte state, ast.C1, in which preserved chromatin accessibility and risk-variant-linked enhancers preferentially engage SNARE-dependent trafficking pathways. Notably, *SOX10* upregulation in mg.C4 and ast.C1 aligns glial responses with phagocytic and proteostatic functions previously linked to disease resilience^81^. Together, these findings suggest that disease-specific genetic risk shapes glial resilience by selectively tuning lysosome-vesicle pathways in distinct cell types, providing a mechanistic explanation for divergent glial responses across tauopathies.

Importantly, we provide direct experimental validation that these computationally defined states represent bona fide biological entities. RNAscope and immunohistochemistry confirm the presence and expansion of *SOX10*⁺/*PLP1*⁺ microglia and astrocytes in human primary tauopathy brain tissue. Functional perturbation experiments further demonstrate that synaptic phagocytosis enhanced *SOX10* activation in human iPSC-derived microglia. Microglia with CRISPRa-induced SOX10 expression, when exposed to disease synaptosomes, display greater induction of proliferative, phagocytic, and lysosome-associated programs compared to synaptosome-treated microglia without SOX10 induction. The induction of these genetic programs in the *SOX10*-induced cells recapitulates the in vivo mg.C4 transcriptional signature.

### Translational implications

Our findings establish a mechanistic framework in which common genetic risk acts through disease-context-specific regulatory elements to tune glial stress responses, rather than uniformly activating inflammatory pathways. By leveraging MPRA-defined functional variants, we refine genetic association signals to nucleotide-resolution regulatory elements and embed them within coherent transcriptional networks. This approach reveals candidate genes and pathways, including lysosomal trafficking, sphingolipid metabolism, vesicle fusion, and phagocytosis, that are selectively engaged in disease-relevant glial states.

These results nominate *SOX10*-, *MEF2C*-, *MEF2D*-, and SNARE-centered regulatory circuits as potential targets of therapeutic intervention aimed at enhancing glial resilience rather than suppressing glial activation globally. More broadly, our study illustrates how integrating functional genomics with single-cell epigenomics can move beyond locus-based genetic association toward actionable regulatory networks, providing a rational framework for prioritizing targets for perturbation-based rescue of disease-associated cellular phenotypes.

### Limitations and future directions

Several limitations warrant consideration. First, while MPRA and CRISPRa experiments provide functional validation, these assays were performed in in vitro microglial models that may not fully capture the complexity of in vivo cellular interactions. Second, our analyses focus on chromatin accessibility and transcriptional regulation, and future studies integrating proteomic and spatial readouts will be important for confirming the specific targets and downstream functional consequences. Third, although we identify convergent regulatory modules across tauopathies, our data are derived from end-stage human brain tissue, limiting our ability to resolve the temporal sequence and causal directionality between tau pathology, chromatin remodeling, and glial state transitions. Future work will benefit from targeted perturbation of prioritized regulatory elements and transcription factors in disease-relevant models, including co-culture and in vivo systems, to directly test their capacity to impact cellular function, such as organellar or functional resilience. Extending this framework to additional neurodegenerative disorders will further clarify how shared and disorder-specific genetic architectures shape glial responses across disease contexts.

In summary, we provide a cross-disorder atlas linking gene regulation, chromatin dynamics and cellular functions across three tau disorders to highlight disorder-specific glial states associated with differential resilience. We characterize epigenomic dynamics and map genetic variants to their targets through CREs, enhancing our understanding of disease regulatory circuits. We provide molecular targets linked to polygenic disease risk, prioritizing genes for experimental validation to inform causal mechanisms and therapeutic strategies. Our data enhance understanding of glial contributions to tauopathies at the single-cell level and underscore the importance of cross-disorder, cell-specific chromatin profiling in brain regions with moderate pathology.

## Methods

### Ethics

This study used de-identified postmortem human brain tissue obtained from the University of Pennsylvania Center for Neurodegenerative Disease Research Brain Bank and the UCSF Neurodegenerative Disease Brain Bank. Tissue collection and distribution by these brain banks were conducted under Institutional Review Board-approved protocols with informed consent from donors or their legal representatives. All specimens used in this study were de-identified and derived exclusively from deceased individuals. In accordance with U.S. federal regulations governing human subjects research (45 CFR 46), this work does not constitute human subjects research; therefore, Institutional Review Board review was not required.

### Sample pathology

Patients autopsied in this study underwent semi-quantitative scoring of neurodegeneration and tau pathology features as previously described^19^. Briefly, neurodegeneration was scored based on hematoxylin and eosin stain and included microvacuolation, astrogliosis, and neuronal loss, each graded on a scale of 0 to 3 (absent, mild, moderate, severe). Tau aggregates were visualized using a monoclonal anti-phospho-tau (pS202) antibody CP13. Pathomorphologies were graded using the same 0-3 scale and included neurofibrillary tangles, Pick’s bodies, neuronal cytoplasmic inclusions, globose tangles, astrocytic plaques, tuft-shaped astrocytes, thorn-shaped astrocytes, tau-positive threads and grains in both gray and white matter, and glial cytoplasmic inclusions. In addition to these scores, we have now added standardized neuropathological data from our samples to this manuscript (Supplementary Data 1). The pathology data for each sample can be found in (Supplementary Data 1). The insula, PreCG, and calcarine cortex exhibit differential vulnerability across diseases, with the insular cortex showing a higher disease burden in PiD and PSP compared with AD (Supplementary Data 1).

### snATAC-seq data processing

The nuclei extraction and sequencing settings of snATAC-seq data matched those described in our published work^19^. Using ArchR version 1.0.3^35^, we first retained cells with TSS enrichment ≥2 and a minimum of 1000 fragments per sample. Additionally, we further filtered out low-quality cells for 14 samples with sample-specific stringent cutoffs based on QC plots (Supplementary Fig. 1). Doublets were removed using the filterDoublets function. Dimensionality reduction was performed using iterative Latent Semantic Indexing with six iterations and 25,000 variable features. We applied the Harmony batch effect correcting for preparation batches (Supplementary Fig. 2C). Clustering was conducted using the addClusters function with a resolution of 0.1, generating 10 main clusters. Marker gene scores for each cluster were calculated using the getMarkerFeatures function, retaining those with an FDR < = 0.01 and log2 fold change (Log2FC) ≥1.25. Canonical marker genes were used to annotate the main brain cell type for each cluster, and two small undefined clusters were filtered out. Finally, we integrated the data with our in-house snRNA-seq dataset^19^ using the addGeneIntegrationMatrix function, ensuring the consistency of cell type annotation (Supplementary Figs. 2D–E). For the downstream analysis, we removed one PreCG sample and one insula sample of the PiD case P2301, defined as an outlier in our previous study^19^. Although calcarine samples passed basic QC metrics such as cell yield and TSS enrichment, clustering analysis revealed substantial inconsistencies in cell-type composition (Supplementary Fig. 21A). Across the two calcarine samples per condition, the oligodendrocyte fraction ranged from 9.14% to 68.82%, a level of variability incompatible with reliable downstream modeling. Given this instability and the limited sample size (*n* = 2), calcarine cortex was excluded from all subsequent analyses. Based on differences in tissue microdissection during sample preparation where white matter was grossly removed from PreCG and not insula samples, the cluster composition of samples exhibited regional heterogeneity and showed greater diversity between brain regions than between diseases (Supplementary Fig. 21A). Neurons were more frequently found in PreCG, with an average cell percentage of 42.6% ranging from 35.2% to 62.2%, and the highest occupation in 67.5% of samples (27 of 40). In contrast, cell proportions of control samples were more stable than disease samples, with 7 out of 9 control samples displaying the highest proportions in neurons. In the mid-insula, oligodendrocytes emerged as the predominant cell type in nearly all cases (94.7%, 36 of 38), with an average of 49.0%, ranging from 34.3% to 66.1%.

### Subcluster identification

We performed iterative LSI and clustering with different resolutions using Louvain to construct subclusters for each cell type. The resolution, which generates clusters with meaningful cell numbers, clear boundaries, and well-defined markers, was selected. We examined the cell proportions among library batches, sequencing batches, and samples (Supplementary Fig. 15). We removed subclusters with less than 100 cells, as well as those contributed by one batch or one sample uniquely (ast.C6, mg.C8, mg.C10, mg.C15). We end up identifying 57 subclusters in total.

To confirm the corresponding cell type of each subcluster, we calculated marker gene scores across all subclusters using ArchR’s getMarkerFeatures function, filtering by FDR < 0.1, and examined the Log2FC of canonical cell type markers (Supplementary Fig. 17B and Supplementary Data 16). In addition to the raw cell set, we created a high-quality cell set consisting of cells with a TSS enrichment score ≥ 4 to replicate the identification (Supplementary Data 17). Subclusters that did not express the corresponding cell-type markers in both datasets (Log2FC > 1) were filtered out. We then removed ambiguous subclusters that highly express markers of more than one cell type. Some potential hybrid clusters were retained when another cell type marker was expressed, but not as the highest. The average gene scores of each subcluster show consistent cell type marker expression (Supplementary Fig. 17B). We identified 50 subclusters, including 10 astrocytes, 10 microglia, 12 neurons, 8 oligodendrocytes, and 10 OPC subclusters. The high-resolution subclustering yielded 50 subclusters with minimal batch or donor-driven structure (Supplementary Figs. 15 and 16). Donor contribution analysis showed that >80% of donors within each diagnostic group contributed at least 1% of cells to every subcluster, indicating balanced donor representation.

### Subcluster annotation

We used marker gene scores identified across subclusters to confirm the cell type identity of subclusters, and marker gene scores identified within each cell type to assign subtype functional categories. To understand the functional diversity among subclusters. We first assigned functional groups based on the significant upregulation of disease signature genes. Next, we determined the marker gene scores within the same cell type (Supplementary Data 12) and conducted enrichment analysis for markers using enrichR^82^. The enriched GO terms (FDR < 0.1) and the associated genes were selected as the functional description for each subcluster relative to the other subclusters within the same cell type.

For astrocytes, *GFAP* was widely expressed in all subclusters, with the lowest in ast.C1 and the highest in ast.C5, based on the marker gene scores identified across all subclusters (Supplementary Fig. 17B). The hierarchical clustering of markers called within astrocytes reveals the four main categories of astrocytes (Fig. 4B, C). Reactive astrocytes, represented by ast.C5, ast.C7, ast.C8, and ast.C9, highly expressed *GFAP*, with slight variations in the marker peaks (Fig. 4K). Ast.C2 was related to synaptic transmission. Ast.C3 was related to antigen presentation, exhibiting marker peaks of *HLA-DMB* (Fig. 4C, K). Both ast.C1 and ast.C4 were myelination-related and oligodendrocyte like, with ast.C1 displaying prominent marker peaks for *KLK6* and *PLP1* (Fig. 4K). Ast.C10 was linked to inflammation and had the lowest MAPT gene score, and ast.C11 was metabolism-related (Fig. 4C). When compared with other astrocyte subclusters, ast.C1 highly expressed oligodendrocyte markers, including *MOBP*, *TF*, and *OPALIN*, as well as disease signature genes found in oligodendrocyte, such as *CNTN2*, *SLC5A11* (Fig. 4K and Supplementary Fig. 17B).

For microglia, we identified a total of 10 subclusters, including two homeostatic states mg.C16 *NAV*^+^, mg.C14 *P2RY13*^+^*CTSS*^+^; two transitional state microglia with both homeostatic and inflammatory marker activation: mg.C11 *TMEM119*^+^*TREM2*^+^, mg.C13 *C1QA*^+^*P2RY12*^+^*TREM2*^+^; one disease-associated microglia (DAM) mg.C12 *APOE*^+^*TYROBP*^*+*^; two myelination-related microglia mg.C4 and mg.C6 (*SOX10*^+^*PLP1*^+^); two synaptic transmission genes enriched states mg.C3 and mg.C7 (*SNCB*^+^*DRD4*^+^), and a T cell activation gene enriched subcluster mg.C9 *CD247*^+^*OPTN*^+^. The later five subclusters, except C6, also express autophagy markers *BPTOR* and *OPTN* (Figs. 4E and 4G). Marker peaks were consistently up-regulated in specific subclusters, such as *PLP1* and *SORT1* in mg.C4, *RIN3* in mg.C13, *CD48* in mg.C9, *TREM2* in mg.C13 and mg.C11, and *LGALS3* in mg.C7 and mg.C14. Compared with mg.C4, mg.C6 exhibited greater chromatin accessibility around *RIN3*, *CD48*, and *TREM2* (Fig. 4J).

All of the 8 oligodendrocyte subclusters are up-regulated genes related to myelination, such as *CNTN2*, *OPALIN*, *CARNS1*, *PLP1*, and *OLIG1* (Supplementary Fig. 17B). Furthermore, 4 of these subclusters (odc.C3, odc.C4, odc.C5, odc.C7) also up-regulated *SLC5A11*, which was associated with lipid transport. Odc.C7 was also inflammatory oligodendrocytes enriched in cytokine receptor binding, and odc.C8 was stress-related with markers enriched in DNA repair. In addition, we annotated 10 subclusters for OPC, three of which up-regulated AD signature genes. Opc.C1 expressed *IL6R*, which was associated with immune response, whereas opc.C8, opc.C9, and opc.C10 expressed *GALR1*, associated with neuronal activity. OPC.C9 and OPC.C10 also expressed *LRP1* related to lipid transport (Supplementary Fig. 17B). For neurons, we identified four inhibitory neurons and eight excitatory neurons (Supplementary Fig. 17B). The four inhibitory neurons consist of four *SLC32A1*^+^ *GAD1*^+^ cells. Among them, three expressed additional markers: *PVALB*^+^(neu.C7), *SST*^+^ (neu.C8) and *ADARB2* + *VIP* + (neu.C6). The four excitatory neuron subclusters expressed layer-specific markers: neu.C5 and neu.C11 were *FEZF2*^+^, neu.C14 was *RORB*^+^, and neu.C13 was *CUX2*^+^.

### Marker comparison of microglia subclusters with a public dataset

We obtained the complete list of marker genes of microglial subtypes from^64^ and compared them with the marker gene scores of microglial subclusters defined in our study. We computed the pairwise Jaccard scores and assessed the significance of the overlap using Fisher’s exact test. *P* values were adjusted for multiple testing using the Benjamini-Hochberg correction. The results, including Jaccard scores and significance levels, were visualized in a heatmap.

### CRE identification and validation

We identified potential cis-regulatory elements in each subcluster under each condition based on peak-to-gene relationships and peak-to-peak co-accessibility using ArchR. We used the addCoAccessibility function to calculate co-accessibility and the addPeak2GeneLinks function to calculate peak-to-gene links. A gene’s promoter peak was defined when the Pearson correlation coefficient between peak accessibility and gene expression was greater than 0.45 (FDR < 0.1). An enhancer peak was defined as any peak that was not a promoter peak but whose accessibility correlated with that of a promoter peak (Pearson coefficient > 0.5 and FDR < 0.1). To validate our candidate enhancers, we collected reference enhancers from 14 public resources, including ENCODE^36^, activity-by-contact (ABC) model predictions^37^, FANTOM5 (Functional Annotation of Mammalian Genomes 5)^38,39^, and three single-cell studies^17,32,40^. We downloaded the peak sets from the ENCODE portal (https://www.encodeproject.org). A total of 2,348,854 human reference CREs spanning 1888 cell and tissue types were obtained from the SCREEN database (https://screen.wenglab.org/). We classified a CRE as “known” if it overlapped with the loci of any reference enhancers and as “unannotated” if it did not.

### Identification of differentially accessible regions

Using ArchR’s iterative peak calling strategy, we generated pseudobulk replicates for each identified subcluster by grouping cells by sample using the “addGroupCoverages” function, followed by peak calling with MACS2 via the “addReproduciblePeakSet” function. The resulting peaks from all subclusters were then merged to create a consensus peak set for the entire dataset. To identify differentially accessible regions between disease and control within each cell type and brain region, we performed differential accessibility testing using the Wilcoxon test in the discovery dataset. For exploratory assessment of chromatin accessibility trends, we applied a more permissive significance threshold (*P* value < 0.001) combined with a more straightforward fold-changes (absolute log2 fold change ≥1.2). To ensure the robustness of DAR calling and reduce potential biases from unequal cell numbers across groups, we performed downsampling by randomly selecting 30 nuclei per sample per cell type, repeating this process 10 times, and re-running the Wilcoxon test for each iteration. To account for potential donor-specific biases in differential accessibility and validate our findings, we recalculated DARs using a linear mixed-effects model implemented in lmerTest::lmer^83^ with the formula: ~ Diagnosis + Number_of_fragments + TSS_Enrichment + (1 | Donor). Only peaks accessible in at least 5% of cells within a certain cell type in a brain region were included in the analysis. This mixed-model analysis, applied to both the discovery and downsampled datasets, yielded DARs consistent with the pseudobulk analysis (P < 0.005), confirming that donor effects did not drive the observed patterns.

### Identification of dynamic and stable peaks per cell type

We first identified cell type-specific peaks within the consensus peak set. We labeled each peak as 1 (present) or 0 (absent) to indicate its presence in each subcluster based on the original peak calling results. These labels were then concatenated to determine peak presence across each cell type. Next, we identified marker peaks for each of the four conditions within each subcluster by comparing one condition against the other three conditions. Cell type-specific disease marker peaks were determined based on *P* value < 0.05 and |log2 fold change | > 1.

### Heritability partition by LDSC

Integrating with GWAS studies of AD^51^, FTD^52^, PSP^53,74^, and MS^64^, we applied stratified linkage disequilibrium score regression (S-LDSC) to partition trait heritability using the baseline model v1.1, which includes 53 functional categories. We used two metrics to measure the contribution of a functional category $C$ to trait heritability: enrichment and standardized effect size ${\tau }_{c}^{*}$^84–86^. The enrichment score evaluates whether the per-SNP heritability ${\tau }_{c}$ is greater in the category than overall $\tau$:

1 $${{\rm{Enrichment}}}=\frac{{h}^{2}({C}_{c})/{h}^{2}}{M({C}_{c})/M}=\frac{{h}^{2}({C}_{c})/M({C}_{c})}{{h}^{2}/M}$$

where ${h}^{2}$ is the heritability and $M$ is the number of SNPs; ${C}_{c}$ represents the category.

Since ${\tau }_{c}$ depends on trait heritability and the size of the annotation, the standardized effect size (τ*) is used to compare ${\tau }_{c}$ between different traits or annotations.

2 $${\tau }_{c}^{*}=\frac{{\tau }_{c\,}{{\rm{sd}}}(c)}{{h}^{2}/M}$$

The statistical significance of ${\tau }_{c}^{*}$ for each annotation was assessed by computing the *P* value assuming the tst statistic ${\tau }_{c}^{*}/{{\rm{s}}}.{{\rm{e}}}.({\tau }_{c}^{*})$ follows a standard normal distribution under the null hypothesis (^84–86^). To correct for multiple testing, Benjamini & Hochberg’s false discovery rate method was applied to adjust the *P* values. We applied LDSC to multiple peak sets, including subcluster-specific peaks, cell type-specific dynamic and stable peaks, dynamic peaks classified as upregulated or downregulated within each disease, and peaks overlapping with transposable elements.

### Dynamic CREs associated with GWAS variants

To identify regulatory circuitry mediated by GWAS genetic variants, we linked CREs with GWAS SNPs based on the following criteria: (1) SNPs localized within CREs ± 2 kb upstream or downstream, and (2) CREs containing a non-GWAS SNP in linkage disequilibrium (LD) with a GWAS SNP. LD scores were calculated by plink^87,88^ with variants in Europeans (1000 Genomes Phase 3). CREs that exhibited dynamic changes ( | Log2FC | > 1) in disease conditions were selected. Genes with dynamic CREs associated with GWAS SNPs (either directly or through LD, R² > 0.8) were used for functional enrichment analysis by ShinyGO 0.80^89^.

### Reproducing dynamic peaks analysis using SEA-AD snATAC-seq

We used publicly available SEA-AD snATAC-seq data from the middle temporal gyrus to replicate findings of dynamic peaks. The SEA-AD dataset includes 24 pre-defined cell subclasses across four conditions: no AD, low AD pathology, intermediate AD pathology, and high AD pathology. We consolidated these subclasses into six major cell types: astrocytes, microglia, oligodendrocytes, OPC, excitatory neurons, and inhibitory neurons, while excluding VLMC and endothelial cells. Utilizing the 218,882 consensus peaks defined by SEA-AD^54^, we first identified cell-type marker peaks by calling marker peaks within each subclass and then aggregating them to the cell-type level. Marker peak calling was performed using Scanpy with FDR < 0.1 and log2 fold change > 1. Subsequently, we identified marker peaks for each of the four conditions within each subclass (FDR < 0.1). We selected condition marker peaks that were also identified as cell-type marker peaks to define cell-type dynamic peaks. Peaks that remained unchanged across all four conditions were classified as stable peaks. Finally, we performed LDSC enrichment analysis using AD GWAS data^51^ to partition AD heritability among cell-type dynamic peaks, stable peaks, and dynamic peaks identified in each condition.

### Massively parallel reporter assay

We performed a lentiviral-based Massively Parallel Reporter Assay (lentiMPRA) to functionally assess the transcriptional regulatory activity of candidate sequences. Oligonucleotides containing each test sequence upstream of a minimal promoter and a barcoded reporter were cloned into a lentiviral backbone. The resulting plasmid library was packaged into lentivirus and transduced into the human microglial cell line HMC3 (ATCC CRL-3304), with an estimated coverage of 100 barcodes per candidate sequence and approximately 100 integrations per barcode. After overnight incubation, the virus was removed, and cells were cultured for an additional two days to allow sufficient expression of the reporter gene. Genomic DNA and total RNA were then extracted, and barcoded reporter transcripts were quantified by high-throughput sequencing. Parallel DNA sequencing of the integrated barcodes was used to normalize for integration efficiency. Regulatory activity was calculated as the RNA/DNA ratio for each barcode, averaged across barcodes per sequence. Functional variants were defined as those showing significantly different regulatory activity between reference and alternative alleles, with a false discovery rate (FDR) below 0.05. Regarding specificity controls, our MPRA design followed established best practices^90^, using a scrambled sequence as the negative control rather than non-regulatory SNPs. Allele-specific effects were quantified with MPRAnalyze^90^, which uses nested generalized linear models to estimate transcription rates of allele pairs and establishes the null expectation through likelihood ratio testing^55^. This design provides an internal, statistically defined baseline, making additional non-regulatory SNP controls unnecessary.

### MPRA and single-nucleus eQTL enrichment analysis

We analyzed the distribution of *P* values, FDR, and log2 fold changes across all MPRA variants, identifying functional MPRA variants with a *P* value < 0.05. We obtained the single nuclei eQTLs from^60^ with an FDR < 0.00, and from^59^ with significant_by_2step_FDR column label as Yes. To assess the enrichment of MPRA frVars and significant eQTLs, we used Fisher’s Exact Test to examine variants overlapping dynamic and stable peaks. An eQTL was considered colocalized with a peak if the eGene matched the gene linked to the peak. We performed variant enrichment analyses in dynamic and stable peaks across different peak types: all peaks, CRE and gene body peaks, CRE peaks alone, and enhancer-only peaks. For genes with differentially accessible enhancers containing MPRA frVars across disease conditions, we conducted functional enrichment analysis using ShinyGO 0.80^89^, selecting enriched terms with an FDR-adjusted *P* value < 0.1.

### Detection of the CRE module linked with frVars or eQTLs

To define co-occurring regulatory modules based on chromatin accessibility across subclusters, we first generated 823 pseudobulked samples, considering two to five replicates per disease within each of the 50 subclusters, using the ArchR strategy. For each peak in each pseudobulk sample, we aggregated peak counts, depth-normalized them, and applied a log2 transformation. The resulting peak-by-pseudobulk matrix was then quantile normalized across samples. Next, we calculated the average peak activity per peak at the subcluster level, and subsetted the matrix for CRE peaks overlapping functional variants. Hierarchical clustering identified co-occurring CRE modules with their subcluster specificity visualized in heatmap. TF regulators of modules were identified by overlapping predicted TFBS of CREs with TF enrichment analysis using MEME.

### cisTopic modeling of variant-linked CRE modules

To identify co-regulated CRE modules harboring all potential functional genetic variants, we began with an ArchR peak-by-cell matrix restricted to microglia and constructed a curated CRE set comprising dynamic microglial enhancers overlapping either MPRA-validated functional variants (frVar; *P* < 0.05) or significant microglial eQTLs, with overlaps retained only when the eQTL target gene matched ArchR peak-to-gene annotations. Peak counts for this CRE subset were binarized and used to fit latent Dirichlet allocation models with cisTopic across a range of topic numbers, selecting the optimal model based on likelihood and perplexity derivatives. Each topic was defined by its top-weighted CREs and interpreted as a regulatory module. Topic activity was quantified across disease groups and microglial subclusters, and modules were annotated by mapping CREs to nearby genes followed by pathway (GO, KEGG) and transcription factor motif enrichment analyses. Finally, CRE modules were integrated with frVar and eQTL metadata and motif calls to identify variant-linked regulatory programs and their putative transcriptional regulators in disease-associated microglial states.

### Slingshot trajectory inference

Microglial nuclei were subset from the ArchR project by subcluster (mg.C4, mg.C12, mg.C16) and brain insula region. To avoid diagnostic composition bias, nuclei were down-sampled so that each disease group (control, AD, PiD, PSP) contributed equal numbers of cells per subtype, defined by the minimum cell count observed across conditions. ArchR gene activity scores were converted to a *SingleCellExperiment* object with metadata retained. Gene activity values were normalized using the *scran* deconvolution workflow and batch effects across preparation batches were corrected with *fastMNN*. The corrected embedding was used to construct a shared nearest-neighbor graph, followed by Louvain clustering and UMAP visualization. Lineages were inferred with *Slingshot* using UMAP coordinates and cluster assignments, with biologically informed root and terminal clusters specified for each analysis. Principal curves were fit to each lineage to obtain lineage-specific pseudotime values and cell-level lineage weights. Pseudotime-associated changes in gene activity and TSS enrichment were modeled using lineage-weighted generalized additive models, and pseudotime-dependent genes were identified using *tradeSeq* by fitting negative-binomial GAMs to gene activity scores with FDR correction.

### Identification of regulatory TFs and their target genes

To determine if a TF motif plays a potential regulatory role, we first calculated the per-cell accessibility for each TF motif using ChromVar^91^ in ArchR. Motif annotations were obtained from the curated CISBP human v2 database TFs (chromVARmotifs::human_pwms_v2, 870 motifs) via the “addMotifAnnotations” function. Background peaks were selected with the “chromVAR::getBackgroundPeaks” function, which matches peaks for GC content and fragment count across all samples using Mahalanobis distance, thereby adjusting for GC bias and background accessibility. Per-cell deviation z-scores were computed with the “addDeviationsMatrix” function, and candidate motifs were retained if their quantile z-scores exceeded 0.5. Subsequently, we calculated the Pearson correlation between TF activity and TF expression across subclusters, identifying motifs with correlation coefficients greater than 0.5 (FDR < 0.1). This process led to the identification of 164 TF motifs with regulatory roles. Additionally, we identified the top regulatory motifs whose ChromVar variability z-score is above the 90th quantile. To explore the TF variability changes in disease, we calculated delta variability between disease and control (delta z-score) and identified the top 10 TFs with the highest delta z-score for each cell type.

We calculated case-control differentially activated TFs for each cell type and subcluster using the ArchR’s getMarkerFeatures function with the Wilcoxon test on the gene score matrix, retaining significant genes with FDR < 0.1. To identify putative target genes for a TF, we first selected genes whose gene scores are positively correlated with TF activity (Pearson coefficient >0.25 and FDR < 0.1). For each gene, we calculated gene-TF linkage scores based on the peaks that are linked to the gene and contain the motif^92^: Within each cell set (e.g., a disease subcluster like PSP ast.C1), we selected peaks containing the motif that also had accessibility positively correlated with gene activity (Pearson coefficient > 0.1 and FDR < 0.1). The linkage score was calculated as the aggregated square of Pearson coefficients of all selected peaks. Finally, we identified genes with linkage scores above the 80th quantile as potential target genes within the given cell set.

### Prediction of TF binding sites in subcluster regulatory elements (in ast.C1 and mg.C4)

To identify TF targets by mediating binding events in accessible chromatin, we predicted TF binding sites on the marker peaks of subclusters using TOBIAS^93^, as described in our previous study^19^. Briefly, we used the TOBIAS functions *ATACorrect* to correct Tn5 insertion bias in mapped ATAC-seq reads and then utilized *FootprintScores* to calculate foot printing scores across peak regions. To estimate the binding positions of individual TFs on marker peaks, we applied *BINDetect* which combines the predicted footprint scores with TF binding motif information. Genes with CREs bounded by any TF were considered as targets of that TF for further analysis.

### Cellular composition change analysis

To infer composition changes between disease and control groups, we employed a linear regression model by Limma^94^ to assess the statistical significance of compositional changes between disease and control groups while adjusting for covariables such as age and post-mortem interval (PMI). A *p* value threshold of <0.05 was applied to determine significance. To replicate the cellular changes in ast.C1 and mg.C4, we performed a bootstrapped subcluster composition analysis. Over 15 iterations, we sampled 20% of cells from the entire dataset and computed subcluster proportions for each condition. We then applied the Wilcoxon rank-sum test (Wilcox. test in R v4.2.2) to compare composition differences between disease and control groups. Bootstrapped p-values were adjusted for multiple testing using the Benjamini-Hochberg method.

### Analysis of single-nucleus RNA-seq-derived subclusters

To validate the snATAC-seq-derived subclusters ast.C1 and mg.C4, we aligned them with pre-identified subclusters from in-house snRNA-seq data. We first aligned the snATAC-seq dataset with the snRNA-seq dataset to determine the mapped RNA subgroup and then identified markers for the mapped subgroup to confirm their consistency with the ATAC subcluster. We restricted the cellular alignment within each cell type and subset both the ATAC and RNA datasets to cell type level using Seurat’s framwork^95^. The snATAC-seq gene activity and snRNA-seq gene expression were normalized separately. Canonical correlation analysis (CCA) was then performed to identify anchors for pairs of cells using Seurat’s FindTransferAnchors function. We retained results with a prediction score greater than 0.5. We found that 100% of mg.C4 and 89 % of mg.C6 mapped to an RNA subgroup insula-microglia-3, and 86% of ast.C1 mapped to insula-astrocyte-3 (Supplementary Fig. 18B). After alignment, the cellular composition changes in mapped RNA subclusters were measured using bootstrapping with 15 iterations with Wilcoxon rank-sum test.

### Epigenomic stability analysis with single-nucleus methylation data

To assess disease-related epigenomic stability at subcluster level, we measured the extent to which disease chromatin accessibility changes occurred at cell type-specific hypermethylated regions in each subcluster, as described in our previous study^19^. First, we categorized cells into four conditions (Control, AD, PiD and PSP) within each subcluster, and called peaks for each condition using MACS2 in ArchR. Second, we integrated public single-nucleus methyl-3C sequencing data^96^ to quantify methylation levels for each ATAC peak. We used ALLCools^97^ functions allc-to-region-count to calculate methylation level and generate-dataset to compute the hyper-methylated score. ATAC peaks were considered methylated if they met the criteria of a hyper-methylation score ≥0.9 and a methylation level > 0. Third, we performed a permutation test (*n* = 10,000) to compare the proportion of chromatin-accessible regions annotated as methylated between disease and control groups. A higher proportion of methylated regions in disease samples suggests a loss of heterochromatin, while a higher proportion in control samples indicates heterochromatin gain in disease, both reflecting epigenomic relaxation or instability. Empirical *p* values were adjusted for multiple testing (*** FDR ≤ 0.001, ** FDR ≤ 0.01, * 0.01 < FDR ≤ 0.05).

### Cell-cell interactions analysis

To infer cellular interactions across conditions, we used the CellphoneDB DEG-based method. First, we imputed expression values for cells identified from snATAC-seq data for each individual. The snATAC-seq data were processed using the Signac package^98^, and alignment with snRNA-seq data was performed using Seurat’s FindTransferAnchors function, employing canonical correlation analysis (CCA). We downsampled each sample to 35% of its total cell count while maintaining the subcluster distribution. For each sample, we calculated the targeted number of cells representing 35% of the total, then randomly sampled from each subcluster in proportion to its original representation within the sample. This approach preserved the subcluster structure after downsampling. Differentially expressed genes (DEGs) were calculated on the downsampled dataset using imputed expression values with Seurat’s FindMarkers function. DEGs for each disease condition were identified through pairwise comparisons with the control samples, while control samples were collectively compared against all three disease conditions. DEG analysis was conducted separately for each cell type and subcluster, retaining up-regulated genes with a log2 fold change (Log2FC) > 0.25 and a false discovery rate (FDR) < 0.1. Finally, we ran CellphoneDB for each of the four conditions using default parameters to infer cell-cell interactions based on the identified DEGs. The differences in the number of cellular interactions between conditions were assessed using a chi-square test, with a false discovery rate (FDR) threshold set at <0.05.

### TF enrichment

TF motif enrichment was carried out using the runAme function from MEME Suite in R. For motif enrichment in cell condition-dynamic peaks compared to stable peaks within each cell type, we used the JASPAR 2022 non-redundant motif database^99^. Other peak sets were analyzed using the CISBP 2.00 Homo sapiens database^100^. Significantly enriched functional TFs were retained with an FDR < 0.1.

### RNAscope and immunofluorescence

Experimental validation was performed using RNAscope and immunohistochemistry (IHC) on formalin-fixed paraffin-embedded (FFPE) human insular brain tissue from one PSP case (sample ID: I1_5) and one control case (sample ID: i3_5_at). Brain tissue was sectioned at a thickness of 6 µm. RNAscope™ assays were performed using an automated Leica Biosystems platform at the UCLA Translational Pathology Core Laboratory. Target RNA-specific oligonucleotide probes used for mRNA of human *SOX10* (CATALOG # 484128-C4) and *PLP1* (CATALOG# 499278) were synthesized by Advanced Cell Diagnostics. GFAP protein was detected by IHC using anti-GFAP antibody (Dako, clone M0761) at a 1:200 dilution. IHC staining was performed after completing the RNAscope procedure. Images were analyzed using Phenochart 2.2.0 with spectral unmixing to minimize background signals. Signals located within or adjacent to nuclei and exceeding the average background intensity were considered positive RNAscope signals. Six regions were randomly sampled per slide ( > 60 astrocytes and > 100 microglial cells) for quantifying astrocytes and microglia with detectable SOX10 signals. Cells were regarded as SOX10-positive when they exhibited either more than one distinct punctate signal in the SOX10 channel or a single bright, prominent signal within the cell, clearly distinguishable from the surrounding background. Independent IHC staining for GFAP and PLP1 was performed on FFPE human insular slides from PiD and control cases (N_PiD=7; N_Control=8) using the same procedure described in previous study^19^. Briefly, paraffin sections were deparaffinized, subjected to antigen retrieval in citrate buffer, blocked with 5% donkey Serum, and incubated with primary antibodies overnight followed by fluorescent secondary antibodies before imaging. The staining used anti-GFAP antibody (CATALOG #173308) from Synaptic Systems (SYSY) at a 1:500 dilution and anti-PLP1 antibody (CST #28702) from Cell Signaling Technology at a 1:150 dilution. Images were captured via Vectra Polaris microscope and analyzed with Imaris 10.1 (RRID:SCR_007370) to identify colocalization signals through spot-distance analysis, with a cutoff between −3 and −1 μm.

### Human iPSC-derived iTF-Microglia cell culture and differentiation

iTF-microglia iPSCs (male WTC11 background) were cultured and differentiated as described^101^. iPSCs were cultured in StemFlex media (Gibco A3349401) on cell culture-treated plates (Thermo Fisher Scientific; assorted Cat. Nos.) coated with Matrigel (Corning 356231) diluted 1:100 in Knockout DMEM (Gibco 10829-018). StemFlex was replaced every other day. When 70–80% confluent, cells were passaged by aspirating media, washing with DPBS (Gibco 14190-144), incubating with StemPro Accutase Cell Dissociation Reagent (Gibco A11105-01) at 37 °C for 7 min, diluting Accutase 1:5 in StemFlex, collecting cells in conicals, centrifuging at 300 g for 5 min, aspirating supernatant, resuspending cell pellet in StemFlex supplemented with 1x CultureCEPT Supplement (Gibco A56799), and plating onto Matrigel-coated plates at a 1:20 dilution.

To differentiate the iPSCs into microglia, iPSCs were grown to 70–80% confluence, then dissociated as described above. Before centrifugation, cells were counted and the appropriate number of cells for differentiation was reserved. The number of cells varied depending on the size of the plate being used: 10,000 cells per well for 96-well plate, 0.5 million per well for 24 well plate, 0.1 million per well for 12-well plate, 0.15 million per well for 6-well plate, 2 million per dish for 10-cm dish and 8 million per dish for 15-cm dish. The reserved cells were then centrifuged at 300 g for 5 min and resuspended in day 0 differentiation medium containing the following: Essential 8 Medium (Gibco A15169-01) as a base, 1x CulureCEPT Supplement, and 2 μg/mL doxycycline (Sigma D9891-5G). iTF-iPSCs were seeded onto double-coated plates (Poly-d-Lysine-precoated Bio plates (Thermo Fisher Scientific, assorted Cat. Nos.; or TC-treated plates with manually coated with poly-D-lysine (Gibco A3890401)) + Matrigel coating). On day 2, medium was replaced with day 2 differentiation medium containing Advanced DMEM/F12 Medium (Gibco 12634-010) as a base medium containing the following: 1x Antibiotic-Antimycotic (Anti-Anti) (Gibco 15240-062), 1x GlutaMAX (Gibco 35050-061), 2 μg/mL doxycycline, 100 ng/mL Human IL-34 (Peprotech 200-34) and 10 ng/mL Human GM-CSF (Peprotech 300-03). Two days later, on day 4, the medium was replaced with iTF-Microglia medium, containing Advanced DMEM/F12 as a base medium and the following: 1x Anti-Anti, 1x GlutaMAX, 2 μg/mL doxycycline, 100 ng/mL Human IL-34, 10 ng/mL Human GM-CSF, 50 ng/mL Human M-CSF (Peprotech 300-25) and 50 ng/mL Human TGFB1 (Peprotech 100-21 C). On day 8, the medium was replaced with fresh iTF-Microglia medium. iTF-Microglia can be cultured for at least 12 more days in iTF-Microglia medium with full medium changes every 3–4 days. When differentiating with induction of the CRISPRi/a cassette, the medium was supplemented with 50 nM TMP (MP Biomedical; Cat. No. 195527) starting at day 0 of differentiation and changed every 2 d to maintain strong knockdown/overexpression.

For dissociation, iTF-Microglia were washed twice with PBS before adding TrypLE Express (Gibco; Cat. No. 12605-028) and incubating for 5-8 min at 37 °C. Cells were diluted 1:5 in Advanced DMEM/F12 and spun down at 300 g for 5 min before resuspending in appropriate media.

### gRNA Transduction of iPSCs

Initially, gRNA sequences for the target genes were cloned into a gRNA expression plasmid with a U6 promoter and Blasticidin resistance via Golden Gate Assembly following to the manufacturer’s protocol (NEB #E1601). Plasmids were sequence verified prior to lentiviral packaging, and 300 uL aliquots were frozen at −80C for later use. For lentiviral transduction, 300 uL of each virus suspension was thawed on ice and added dropwise to a well of a 6-well plate containing 2.5 × 105 iPSC cells in complete growth medium supplemented with 8 ug/mL protamine sulfate. Cells were allowed to incubate for 72 h, then cells were split and seeded into a fresh 6-well plate in complete growth medium supplemented with 5 ug/mL Blasticidin. Selection media was replaced every 2–3 days until the appearance of visibly selected cells began to grow. Cells were split and expanded for future use. iTF-microglia iPSCs were plated at a density of 250,000 cells per well of a 6-well plate and transduced with lentivirus carrying the TCV1001-pCLIP-gRNA-EFS-Blasticidin vector containing selected sgRNA sequences. The SOX10 sgRNA protospacer sequence was GGGGCCTGGGCAGTAGGGCA. Lentiviral packaging and transduction were performed by the UCLA Molecular Screening Shared Resource (MSSR). Transduced iPSCs were then selected with 5 µg ml−1 blasticidin for 5 days, then passaged and cultured as described above.

### Human brain synaptosome isolation

Human postmortem brain tissue was processed to isolate synaptosomes as described^102^. Briefly, frozen tissue was gently thawed on ice and homogenized in ice-cold 0.32 M sucrose buffer supplemented with protease and phosphatase inhibitors using low-speed mechanical homogenization. Homogenates were sequentially centrifuged to remove nuclei and debris (P1) and to obtain a crude synaptosomal fraction (P2). The P2 fraction was washed and resuspended in sucrose-based medium.

### iTF-microglia synaptosome treatment

iTF-microglia were prepared in 24-well plates as described above and treated with human brain-derived synaptosomes at a concentration of 20 μg/mL for 24 hours. Cells were then dissociated as described above and resuspended in Zymo 1X DNA/RNA Shield™ (Zymo Research; Cat. No. R1200-125). Bulk RNA-sequencing was performed by Plasmidsaurus using Oxford Nanopore Technology with custom analysis and annotation.

### Fluorescence imaging

iTF-microglia were prepared in 8-well chamber slides (Ibidi; Cat. No. 80806) as described above with a seeding density of 30,500 cells per well and treated with human brain-derived synaptosomes at a concentration of 20 μg/mL for 24 h. Next, media was replaced with fresh iTF-microglia media supplemented with 50 nM LysoTracker® Red DND-99 (Thermo Fisher Scientific; Cat. No. L7528) and 1 μM LysoSensor™ Blue DND-167 (Thermo Fisher Scientific; Cat. No. L7533) and cells were incubated for 30 minutes. The media was then replaced with fresh iTF-microglia media supplemented with 1 μg/mL DiD (Thermo Fisher Scientific; Cat. No. D7757) and cells were incubated for 5 minutes. Next, the cells were washed once with Advanced DMEM/F12 and then fresh iTF-microglia media was added to each well. Fluorescence imaging was performed at the UCLA Broad Stem Cell Research Center Microscopy Core with the Nikon AX R with NSPARC using NIS-Elements acquisition software.

### Reporting summary

Further information on research design is available in the Nature Portfolio Reporting Summary linked to this article.

## Supplementary information

Supplementary Information

Supplementary Data 1-17

Reporting Summary

Transparent Peer Review file

## Source data

Source Data

## Data Availability

Single-nucleus RNA-seq and ATAC-seq data used in this study were previously generated and are available through Synapse under accession syn52074156 (https://www.synapse.org/Synapse:syn52074156/files/). Single-nucleus CUT&Tag data were previously generated and deposited in Synapse under accession syn53191971 (https://www.synapse.org/Synapse:syn53191971/files/). The publicly available datasets we incorporated in this study include single-cell ATAC-seq data of SEA-AD^54^ MTG; and cell-type-specific enhancer peak sets from single-cell studies including syn26670419 (https://www.synapse.org/Synapse:syn26670419)^17^ (under controlled access, request for access can be made on the Synapse portal), ATAC-seq, ChIP-seq and PLAC-seq datasets for each brain cell type available on https://genome.ucsc.edu/s/nottalexi/glassLab_BrainCellTypes_hg19^32^, and single-cell chromatin accessibility data from the human brain available on https://catlas.org/catlas/^40^. Single-nucleus eQTL datasets from the human brain were obtained from two previously published studies: available through Synapse under accession code syn52335732 (10.7303/syn52335732)^59^ and on Zenodo (10.5281/zenodo.5543734)^60^. GWAS summary statistics were obtained for AD from http://ftp.ebi.ac.uk/pub/databases/gwas/summary_statistics/GCST90027001-GCST90028000/GCST90027158/^51^, for PSP from https://dss.niagads.org/open-access-data-portal/#NG00169^53^ and https://dss.niagads.org/datasets/ng00045/^74^ and for FTD on https://rdr.ucl.ac.uk/articles/dataset/IFGC_Summary-statistics_Data-sharing/13042166^52^. Human reference cis-regulatory elements were obtained from the SCREEN database (https://screen.wenglab.org/) and peak sets from the ENCODE portal (https://www.encodeproject.org) with the following identifiers: ENCFF198KYT, ENCFF791URB, ENCFF395QLP, ENCFF815WRM, ENCFF024XNY, ENCFF124JXP, ENCFF155FWO, ENCFF243CHP, ENCFF283LVU, ENCFF307QYO, ENCFF448UVY, ENCFF509IXE, ENCFF528AIU, ENCFF586OIU, ENCFF595JKW, ENCFF693JGW, ENCFF730HDE, ENCFF756JDB, ENCFF788DLD, ENCFF810ZTP, ENCFF812QHM, ENCFF860TAY, ENCFF861YME, ENCFF878EFJ, and ENCFF983DQA. Source data are provided with this paper.

## Code availability

All original code generated in this study is available on GitHub [https://github.com/rexachgroup/pci_snATAC] and archived at Zenodo [10.5281/zenodo.18904265]^103^.
