# Supplementary material for: Single-nucleus epigenomic dysregulation unmasks genetic risk-associated neurodegenerative glia states
Source: Nat Commun. 2026 May 14;17:6439. doi: 10.1038/s41467-026-73007-1 (PMC13376791; doi:10.1038/s41467-026-73007-1)
Supplement: Supplementary file 1 — Supplementary Information [file 41467_2026_73007_MOESM1_ESM.pdf]

Supplementary Table 1. Definitions of peak categories

Term	Definition
Consensus peak set	A unified collection of reproducible peaks created through an iterative overlap merging process in ArchR. Peaks are first identified in pseudo-bulk replicates per subcluster, then merged across replicates and subclusters using fixed-width (501 bp) peaks and iterative overlap removal to prevent daisy-chaining. This final set provides a stable reference for all subsequent analyses.
DAR	Differentially accessible regions where peaks show significant differences in accessibility between disease and control groups.
Marker peaks	Peaks uniquely enriched in a specific cluster compared to others.
Dynamic peaks	Peaks whose accessibility significantly varies in one condition (Control, AD, PiD, PSP) relative to the other three.
Stable peaks	Peaks with no significant changes across all conditions (control and disease groups).
CRE	Cis regulatory elements, mainly enhancers, identified via peak-peak co-accessibility and peak-gene linkage using integrated snATAC-seq and snRNA-seq.
DA-CRE	Cis-regulatory elements that differ in accessibility between disease and control groups.

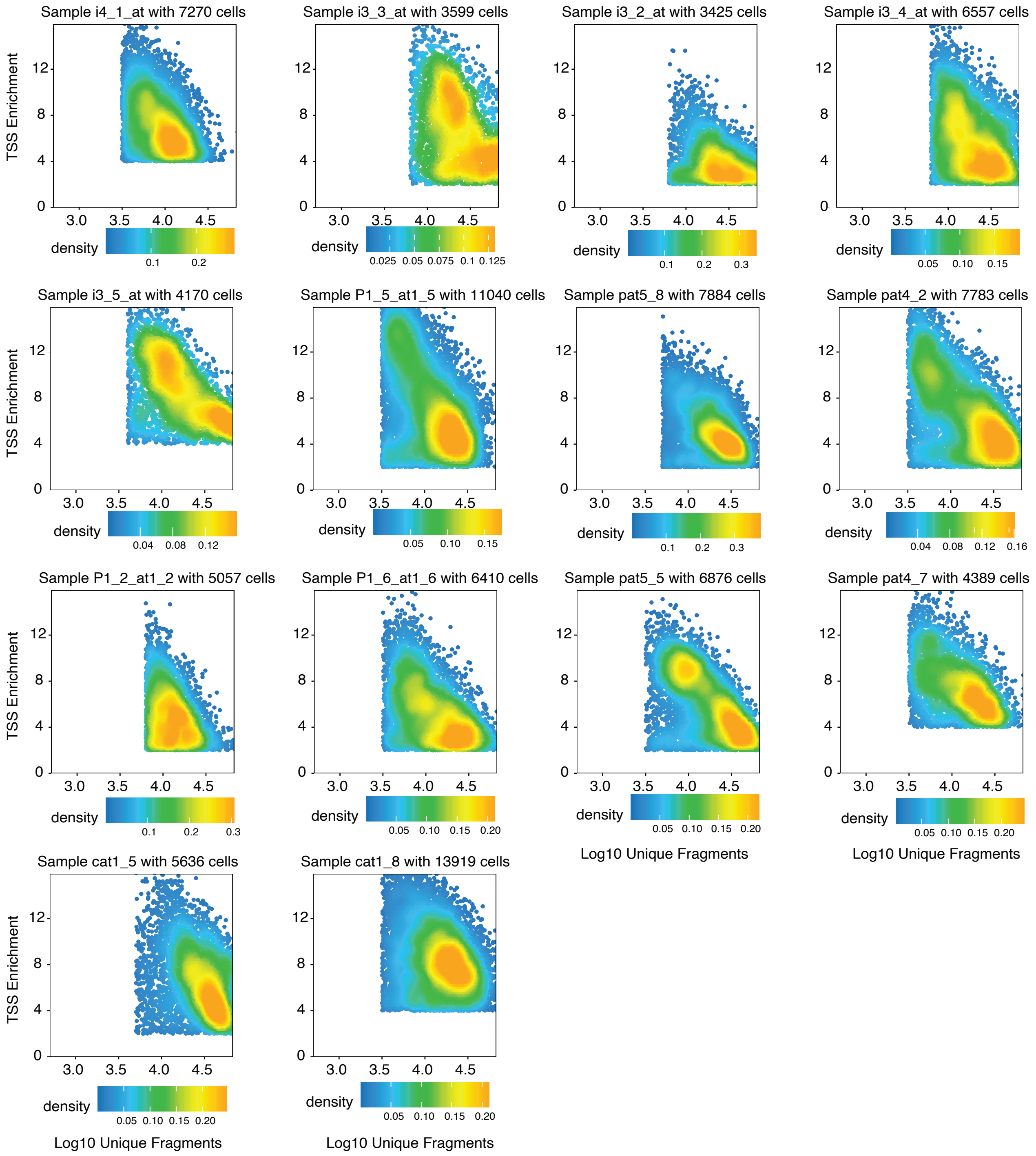

Supplementary Figure 1. Quality control plot for 14 samples with sample-specific thresholds.

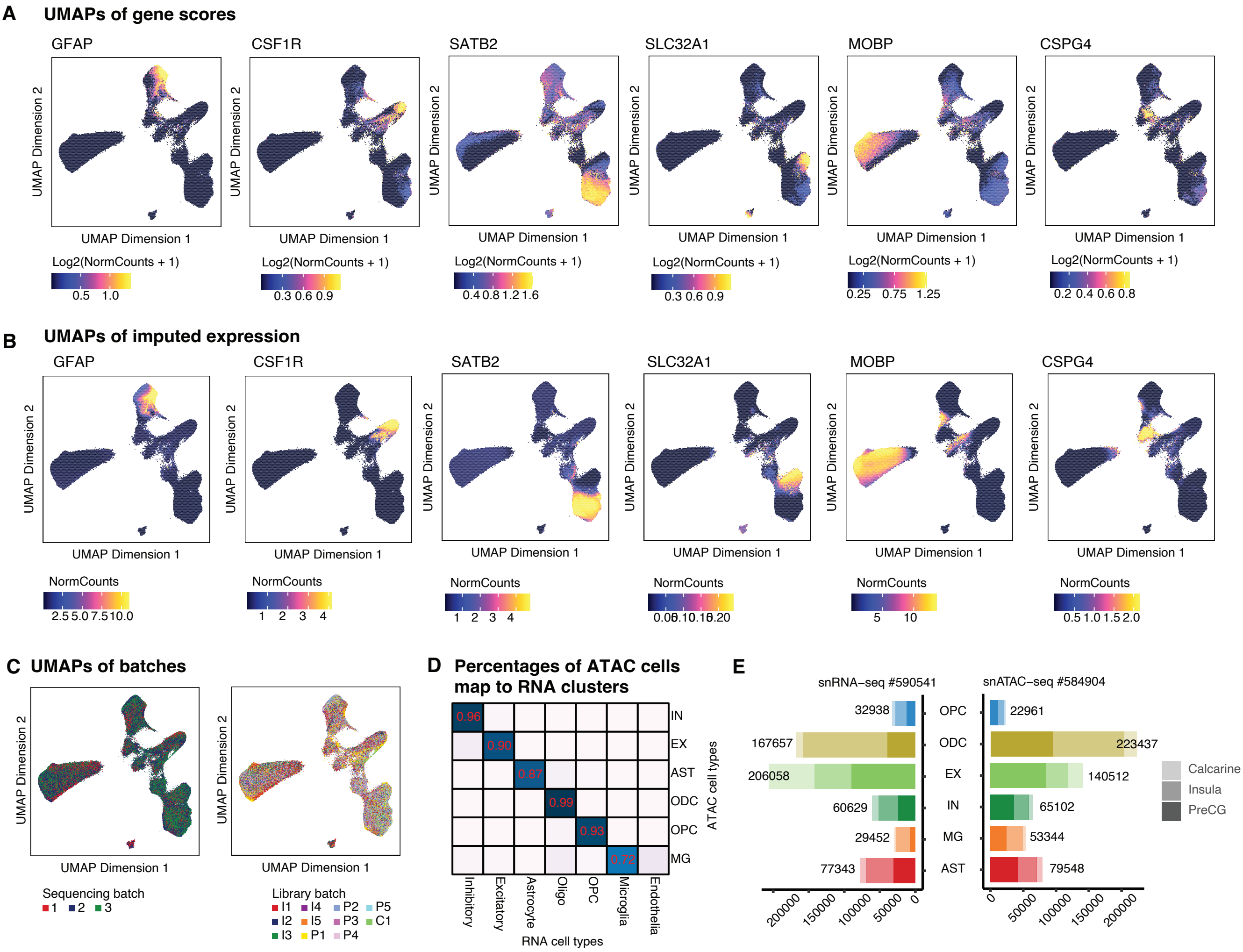

Supplementary Figure 2. Cellular abundance and gene signatures among cell types.

(A-B) UMAP plots displaying gene scores (A) and imputed expression (B) for canonical markers of each cell type.

(C) UMAPs of cells colored by sequencing and library batches.

(D) Alignment between snATAC-seq and in-house snRNA-seq showing the percentage of ATAC cells (rows) mapped to RNA cell types (columns), performed using Seurat's Canonical Correlation Analysis (CCA).

(E) Bar plot showing the number of cells detected per cell type in the snATAC-seq and snRNA-seq datasets.

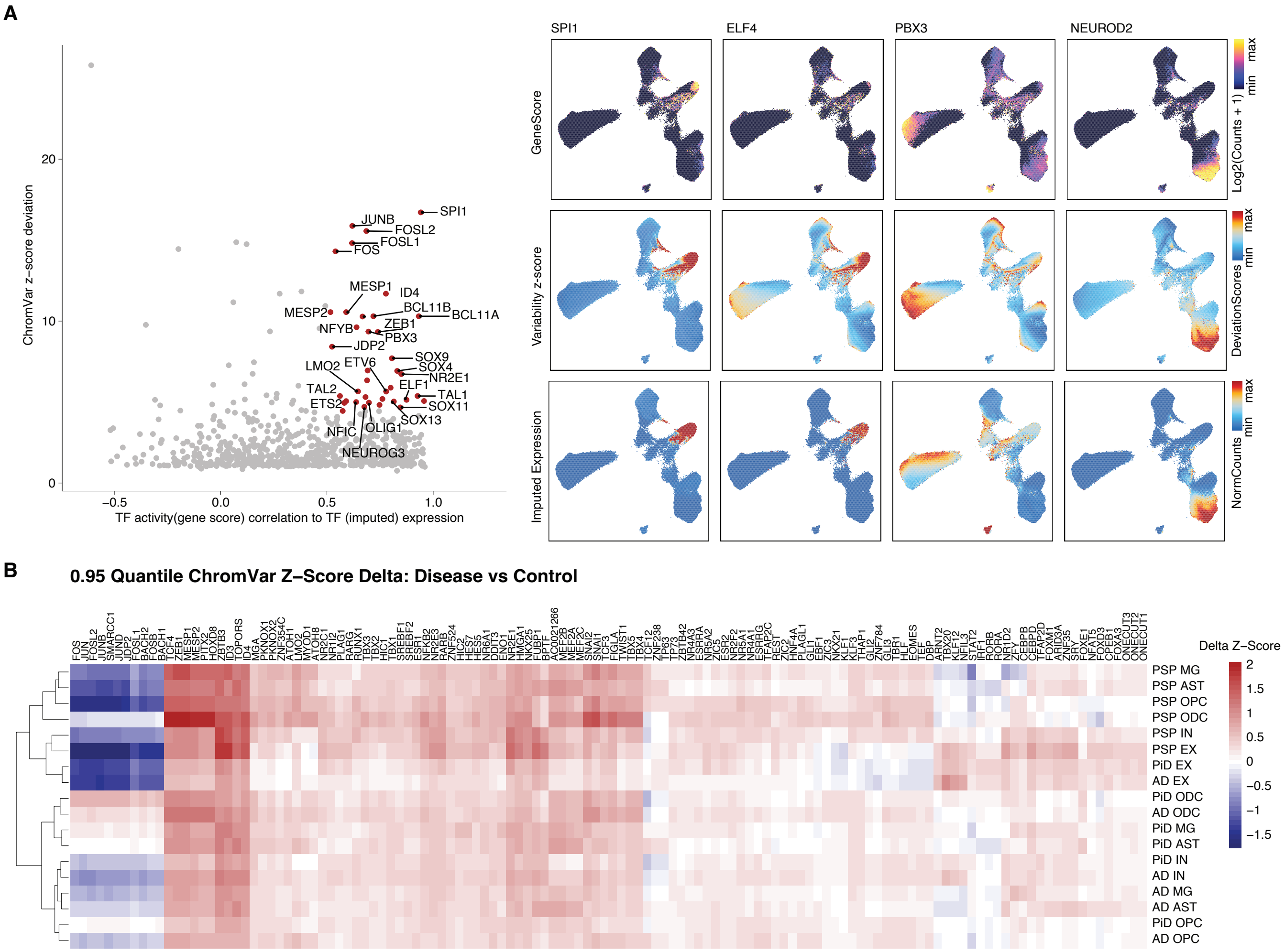

Supplementary Figure 3. TFs with regulatory roles across cell types and diseases.
(A) Prioritization of TFs with regulatory roles across subclusters. The x-axis shows the Pearson correlation between TF activity and TF expression, and the y-axis shows TF variability defined by ChromVar. Top TFs with a z-score deviation above the 90th percentile and a correlation coefficient > 0.5 between activity and expression are highlighted in red (left). UMAP plots on the right depict the gene score, variability z-score, and gene expression of selected TFs.
(B) The delta TF variability for disease and control groups.

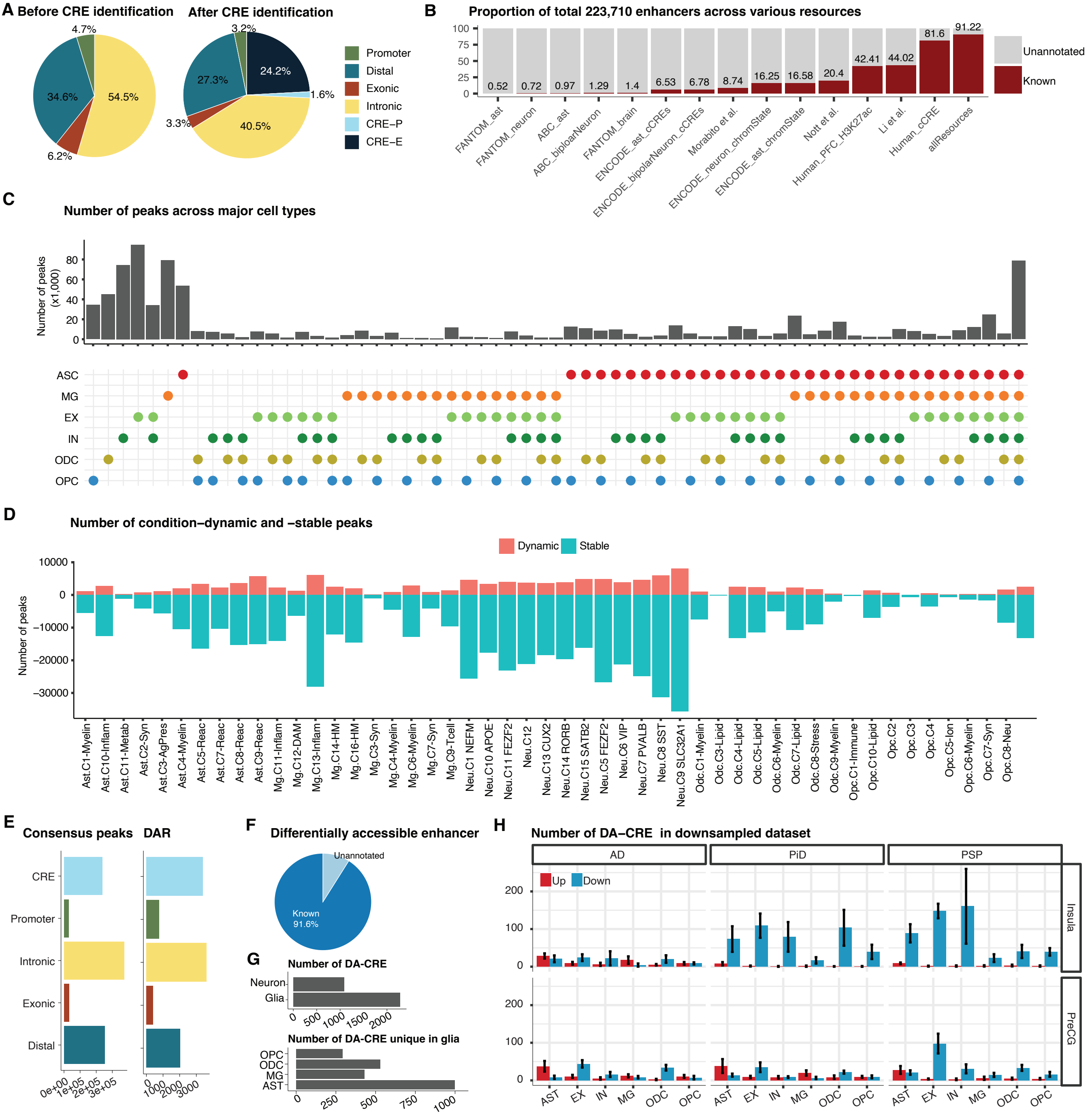

Supplementary Figure 4. Characterization of CREs across genomic features, reference resources and diseases.
(A) Pie chart depicting the distribution of consensus peaks across genomic contexts (CRE, promoter, intronic, exonic, or distal regions) before and after CRE identification. (B) Bar plot showing the percentage of snATAC-seq-derived enhancers overlapping reference enhancer annotations from multiple resources. Human_cCRE denotes the human candidate cis-regulatory elements (cCREs) from ENCODE, and Human_PFC_H3K27ac denotes bulk H3K27ac ChIP-seq from human prefrontal cortex generated by ENCODE. Additional references were obtained from ENCODE, FANTOM, and ABC databases. Experiment IDs are listed in Methods. (C) Bars indicate the number of peaks for each cell-type combination, while dots denote the presence of peaks in individual cell types, distinguishing cell-type-specific and shared peaks. (D) Bar plot displaying the number of dynamic and stable peaks detected in each subcluster. (E) Bar plots depicting the distribution of consensus peaks and DARs across genomic contexts (CRE, promoter, intronic, exonic, or distal regions). (F) Pie chart of differentially accessible enhancers categorized as known or novel. (G) Bar plots showing the number of differentially accessible CREs in glia and neurons (top) and glia-specific CREs by glia types (bottom). (H) Bar plot displaying the number of differentially accessible CREs detected in the downsampled dataset, categorized into up-regulated and down-regulated peaks.

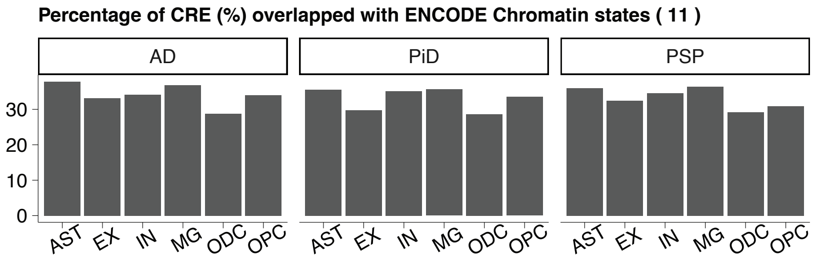

Supplementary Figure 5. Overlap of disease-specific CREs with ENCODE chromatin states. Disease-specific cis-regulatory elements (CREs) were intersected with active chromatin states defined in the human brain by ENCODE, including active enhancers (EnhA), genic enhancers (EnhG), weak enhancers (EnhWk), bivalent enhancers (EnhBiv), active transcription start sites (TssA), flanking TSS regions (TssFlnk), upstream flanking TSS regions (TssFlnkU), downstream flanking TSS regions (TssFlnkD), and bivalent/poised TSS regions (TssBiv)**.**

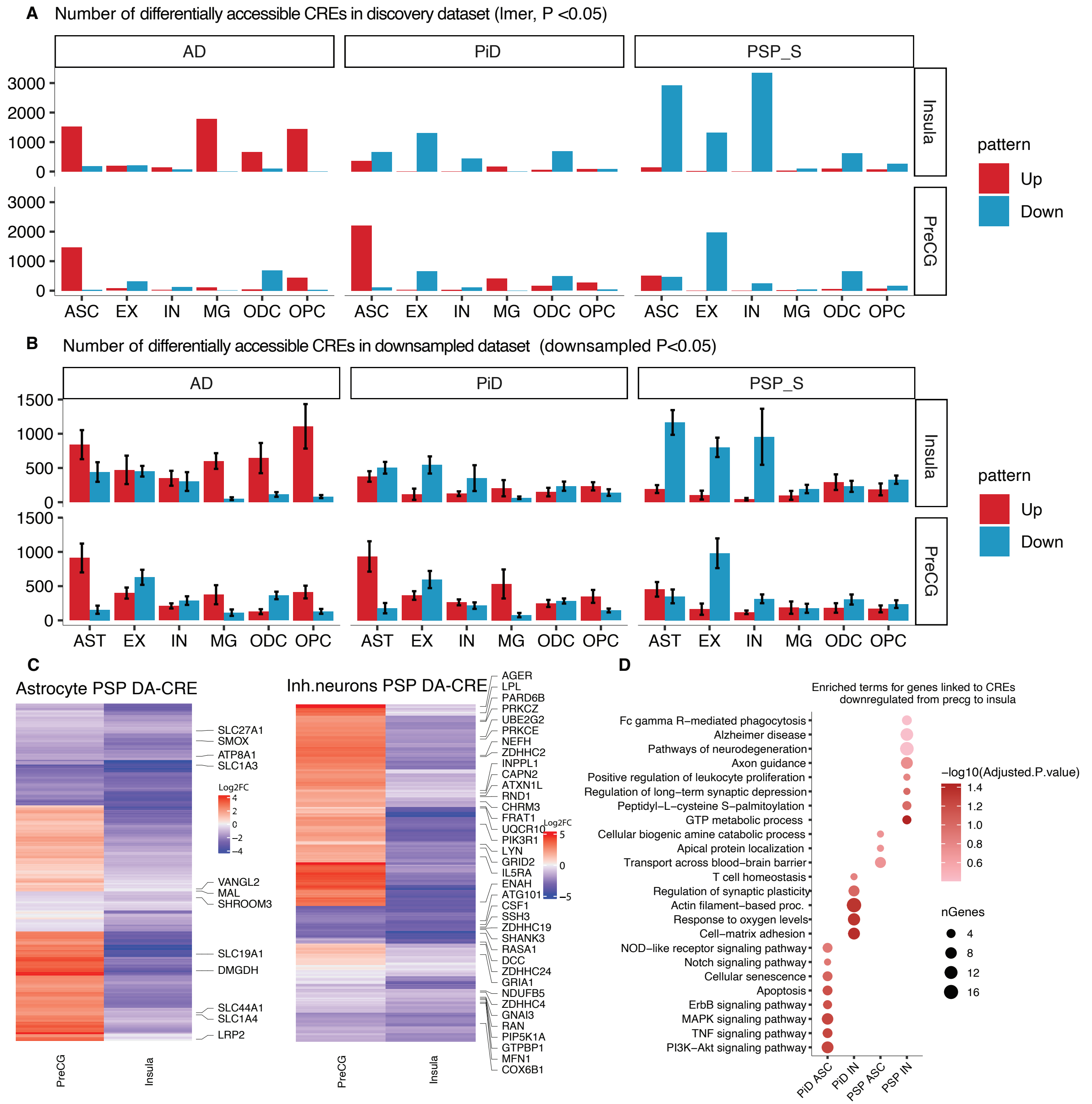

Supplementary Figure 6. Case-control differentially accessible CREs across human brain cell types.
(A-B) Numbers of differentially accessible cis-regulatory elements (DA-CREs) per cell type and brain region identified (A) in the discovery dataset using a two-sided linear mixed-effects model (lmer) and (B) in a downsampled dataset using the two-sided Wilcoxon rank-sum test. DA-CREs with $P < 0.05$ were included to illustrate overall trends. (C) Heatmaps showing PSP-associated DA-CREs upregulated in the precentral gyrus (PreCG) and downregulated in the insula for astrocytes (left) and inhibitory neurons (right). (D) Gene Ontology (GO) terms and KEGG pathways enriched among genes linked to the DA-CREs shown in (C).

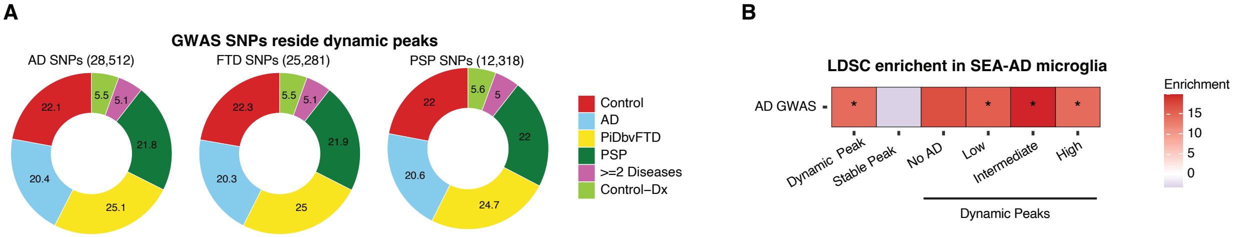

Supplementary Figure 7. Additional analysis of dynamic peaks across diseases.
(A) Pie charts showing the distribution of GWAS SNPs located in dynamic peaks across conditions. (B) AD heritability enrichment in dynamic and stable peaks within microglia in the SEA-AD dataset.

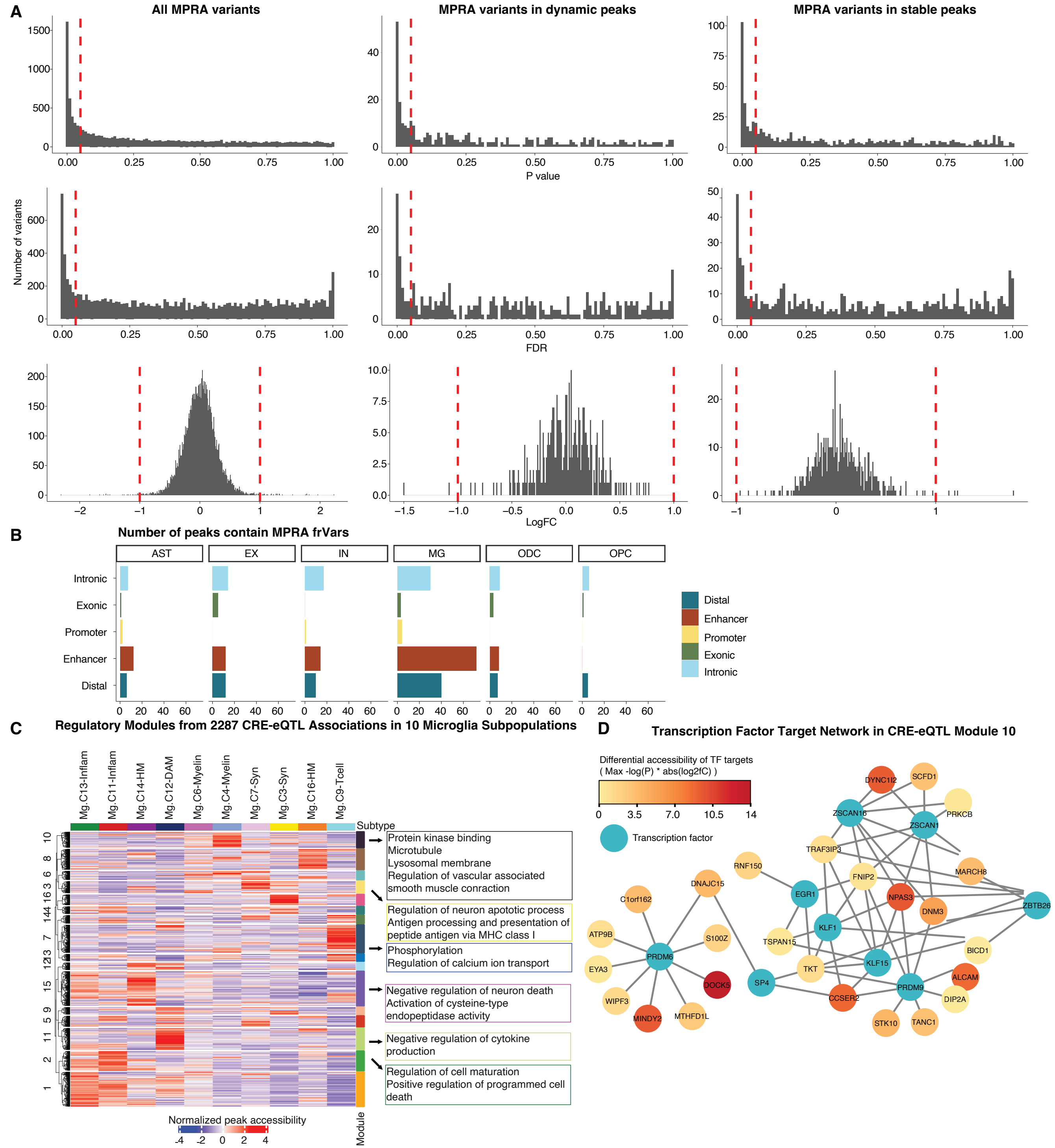

Supplementary Figure 8. Additional analysis of MPRA. (A) Distribution of p-value, FDR, and effect size for all MPRA variants (left), MPRA variants in dynamic peaks (middle), and stable peaks (right). (B) Bar plot displaying the number of cell type-specific peaks containing MPRA frVars, with peaks categorized by gene context (distal, enhancer, promoter, exonic, or intronic). (C) Heatmap showing regulatory modules of CREs with eQTLs across microglial subtypes. Peak accessibility in pseudobulked samples was log2-transformed after depth normalization, and the mean values of subcluster were quantile normalized. Functional enrichment of CRE-linked genes was analyzed using enrichR.(D) TF-target network of module 10 driven by nine key TFs was specifically activated in mg.C4, targeting CREs linked to genes enriched in vesicle-mediated transport and lysosomal functions. CREs linked to target genes colored according to their maximal differential accessibility score, calculated as -log(P-value) x Ilog2FCI from marker peak calling.

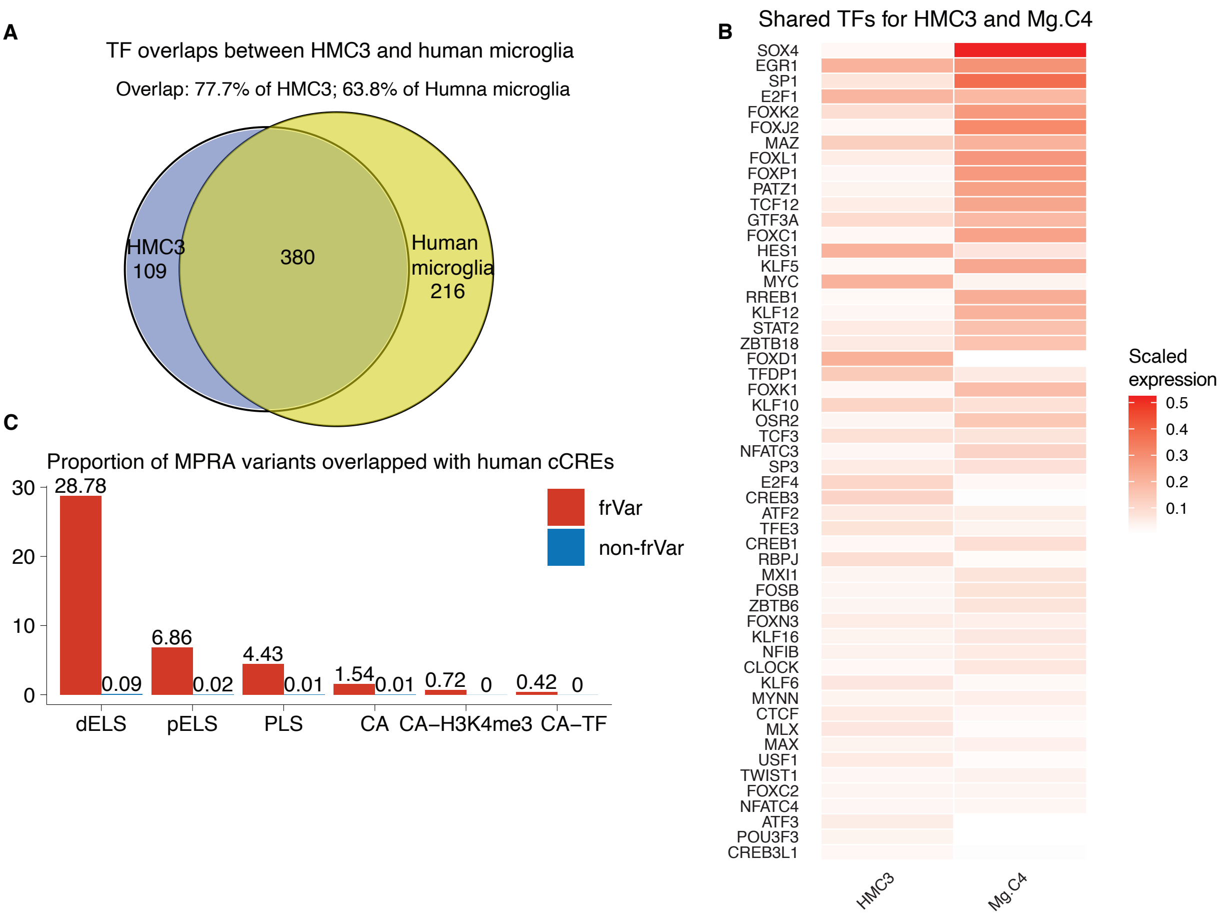

Supplementary Figure 09. Validation of MPRA construct design.

(A) Overlap between TFs expressed in HMC3 bulk RNA-seq and human microglia. HMC3 TFs were defined as the top 50% of TFs ranked by expression level, whereas mg.C4 TFs were defined as those active in more than 10% of cells.

(B) Scaled expression and enrichment values for TFs shared between HMC3 and mg.C4, using normalized TPM values for HMC3 and enrichment false discovery rates (FDRs) for mg.C4. Values were scaled to the [0-1] range within each dataset.

(C) Overlap of MPRA-tested variants with human consensus candidate cis-regulatory elements (cCREs) from ENCODE, categorized by CRE class. CRE classification is based on biochemical signatures across biosamples and proximity to the nearest GENCODE transcription start site (TSS): distal enhancer-like signature (dELS); proximal enhancer-like signature (pELS); promoter-like signature (PLS); chromatin-accessible elements with low H3K4me3, H3K27ac, and CTCF signals (CA); chromatin-accessible elements with high H3K4me3 but low H3K27ac, not within 200 bp of a TSS (CA-H3K4me3); and chromatin-accessible elements with low H3K4me3/H3K27ac/CTCF signals that overlap transcription factor binding clusters (CA-TF).

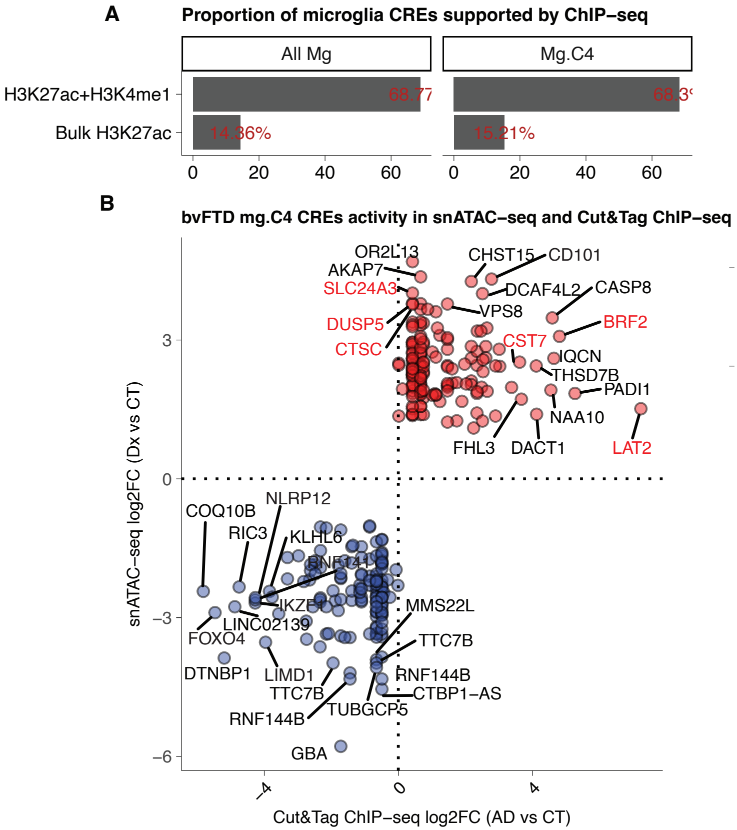

Supplementary Figure 10. Microglia CREs refined by single-cell histone modifications.
(A) Overlap of microglial cis-regulatory elements (CREs) with histone modification marks. Bulk H3K27ac ChIP-seq data from human prefrontal cortex (PFC) (top) and a single-nucleus CUT&Tag dataset profiling H3K27ac and H3K4me1 (bottom) were used to refine enhancer annotations in all microglia and in the mg.C4 subtype.
(B) Disease-associated CRE activity in mg.C4 showing concordant changes between single-nucleus CUT&Tag data (Alzheimer's disease vs. control) and chromatin accessibility differences in this dataset (Pick's disease vs. control). Features with FDR < 0.1 are shown.

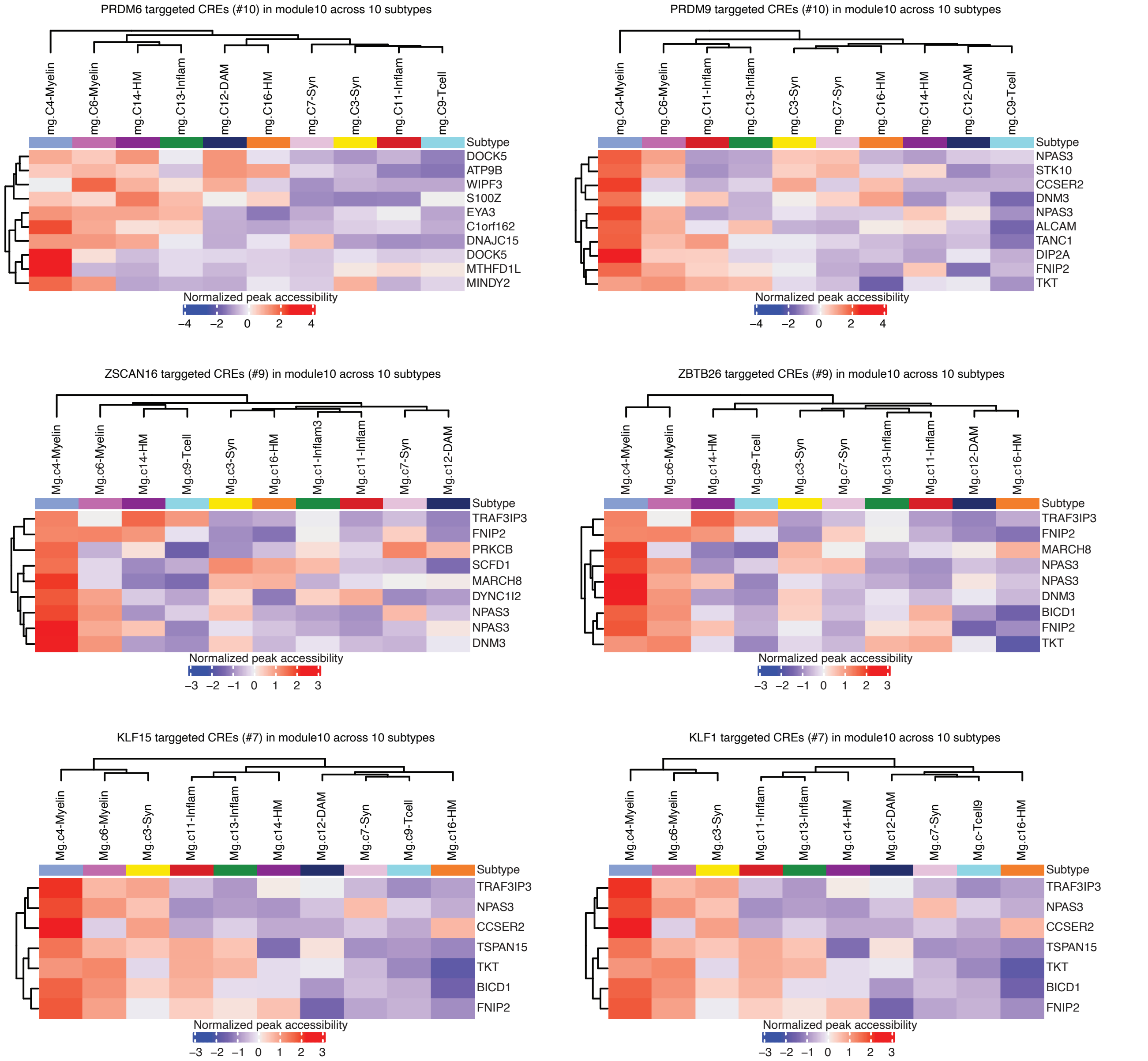

Supplementary Figure 11. Key TF regulators of module10 drive CRE activation in mg.C4. Heatmaps show the normalized chromatin accessibility of CREs bound by the corresponding TF across microglial subclusters, with CRE-linked genes displayed. Peak accessibility in pseudobulked samples is log_2-transformed after depth normalization, and the mean values for each subcluster are quantile n**ormalized.**

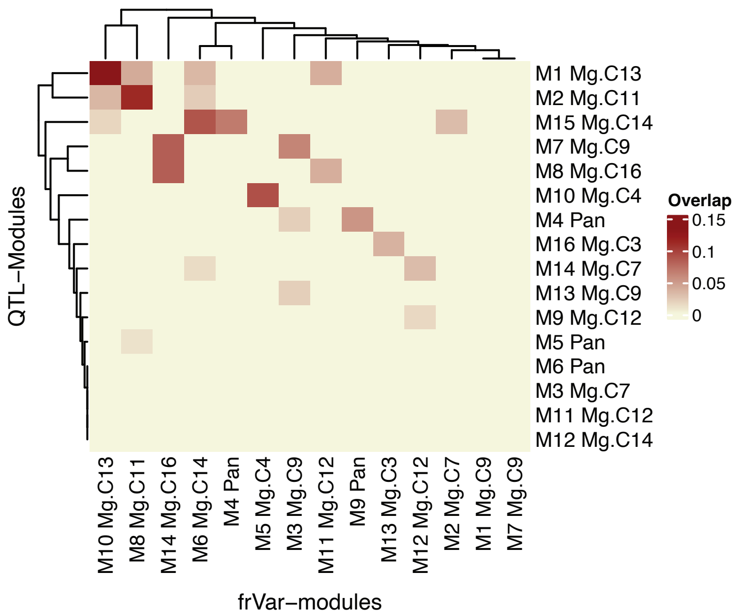

Supplementary Figure 12. Convergence between MPRA-validated functional variant modules and microglial eQTL-derived CRE modules. Microglial sn-eQTL CRE modules (rows) were compared with frVar-derived modules (columns) using fractional CRE overlap, defined as the intersection divided by the smaller module size. Of the 14 frVar modules, 12 aligned with a corresponding eQTL module, and 9 of these module pairs exhibited consistent microglial state specificity.

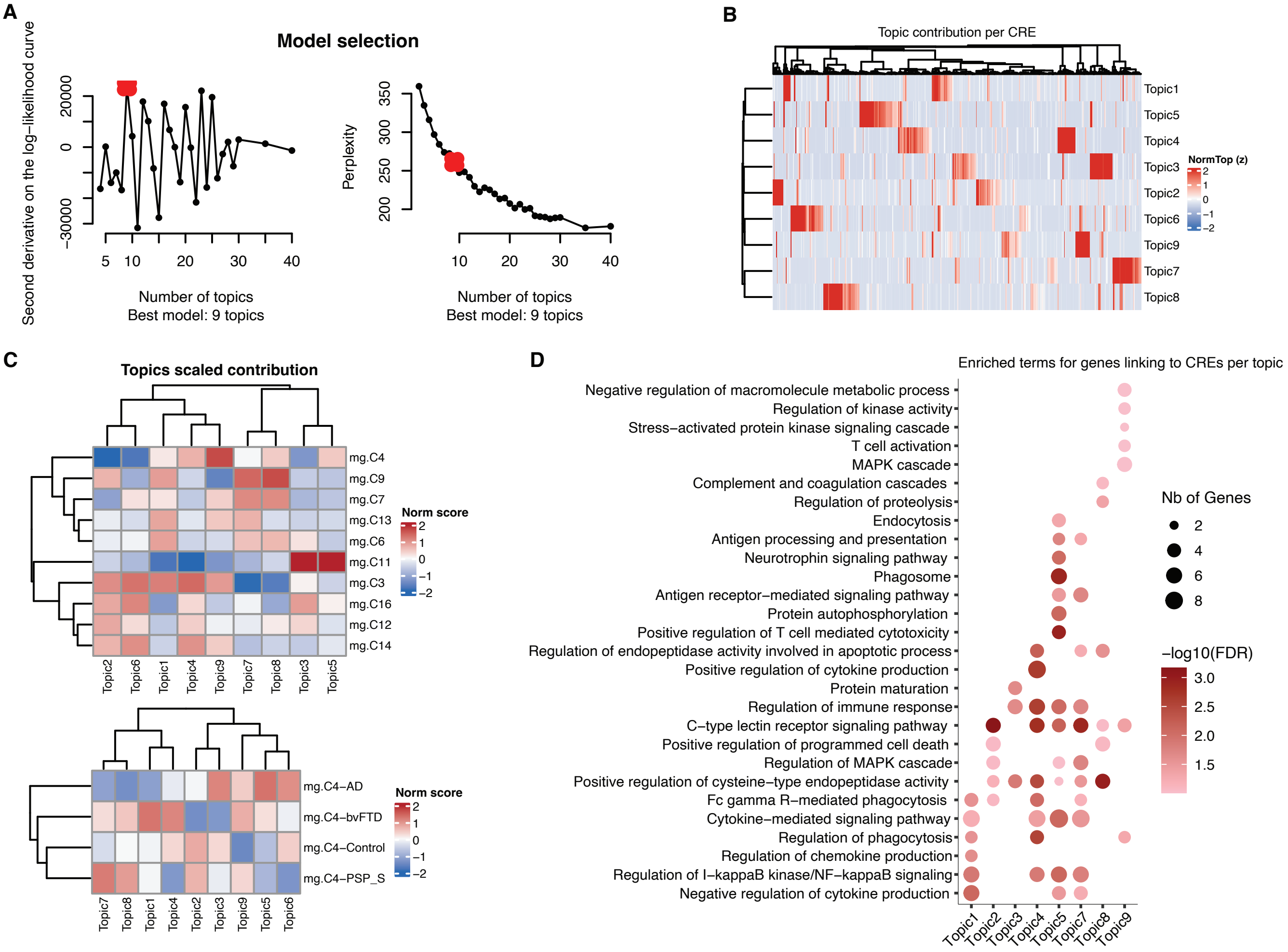

Supplementary Figure 13. Regulatory programs for CREs containing frVar or eQTLs.
(A) Selection of topic numbers for microglial CREs containing frVar or eQTLs using the derivative method in cisTopic.
(B) Heatmap showing topic contributions across CREs.
(C) Comparison of relative topic contributions across microglial subtypes and in PiD-associated mg.C4.
(D) Top enriched TFs and associated functional pathways for each topic.

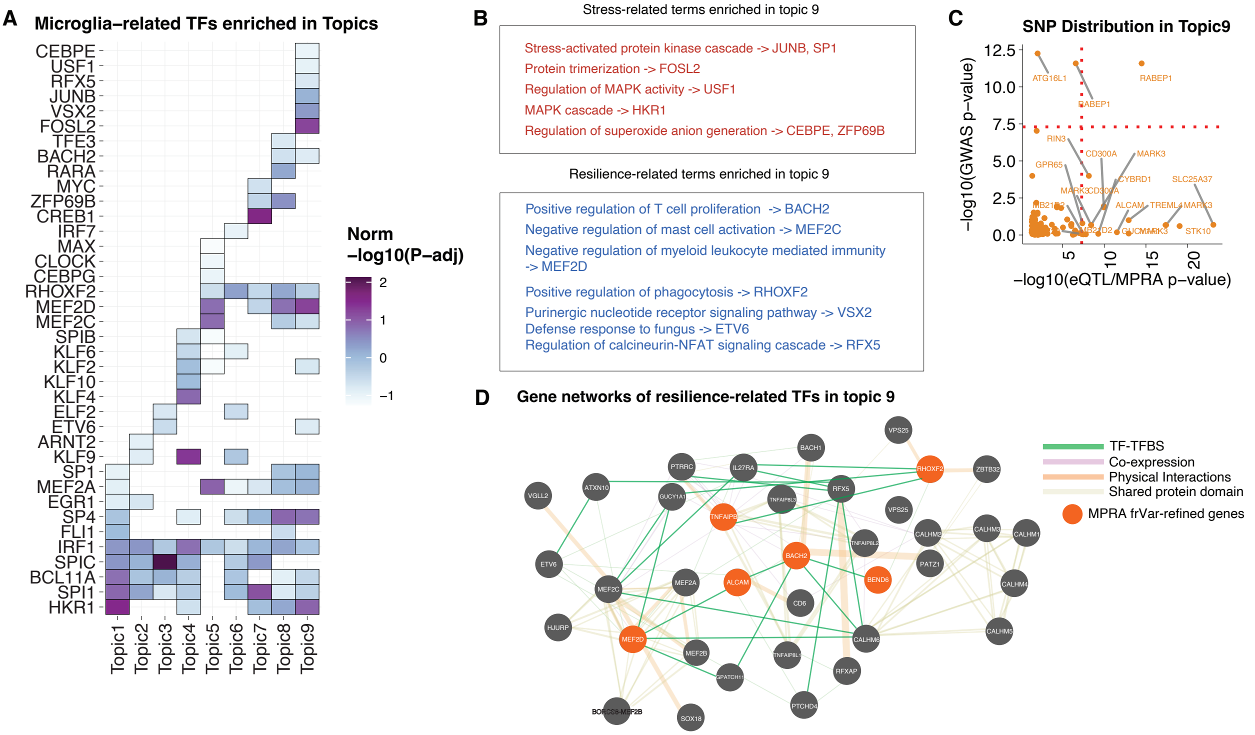

Supplementary Figure 14. Regulatory programs associated with CREs containing frVar or eQTLs linked to disease.
(A-B) Enriched transcription factors (A) and associated functional pathways (B) across topics.
(C) Distribution of genetic variants within Topic 9, emphasizing significance in AD GWAS and functional assays.
(D) Gene network for Topic 9 generated using GeneMANIA, illustrating target genes regulated by resilience-related transcription factors. Genes refined using MPRA-validated functional variants (frVars) are highlighted as orange nodes.

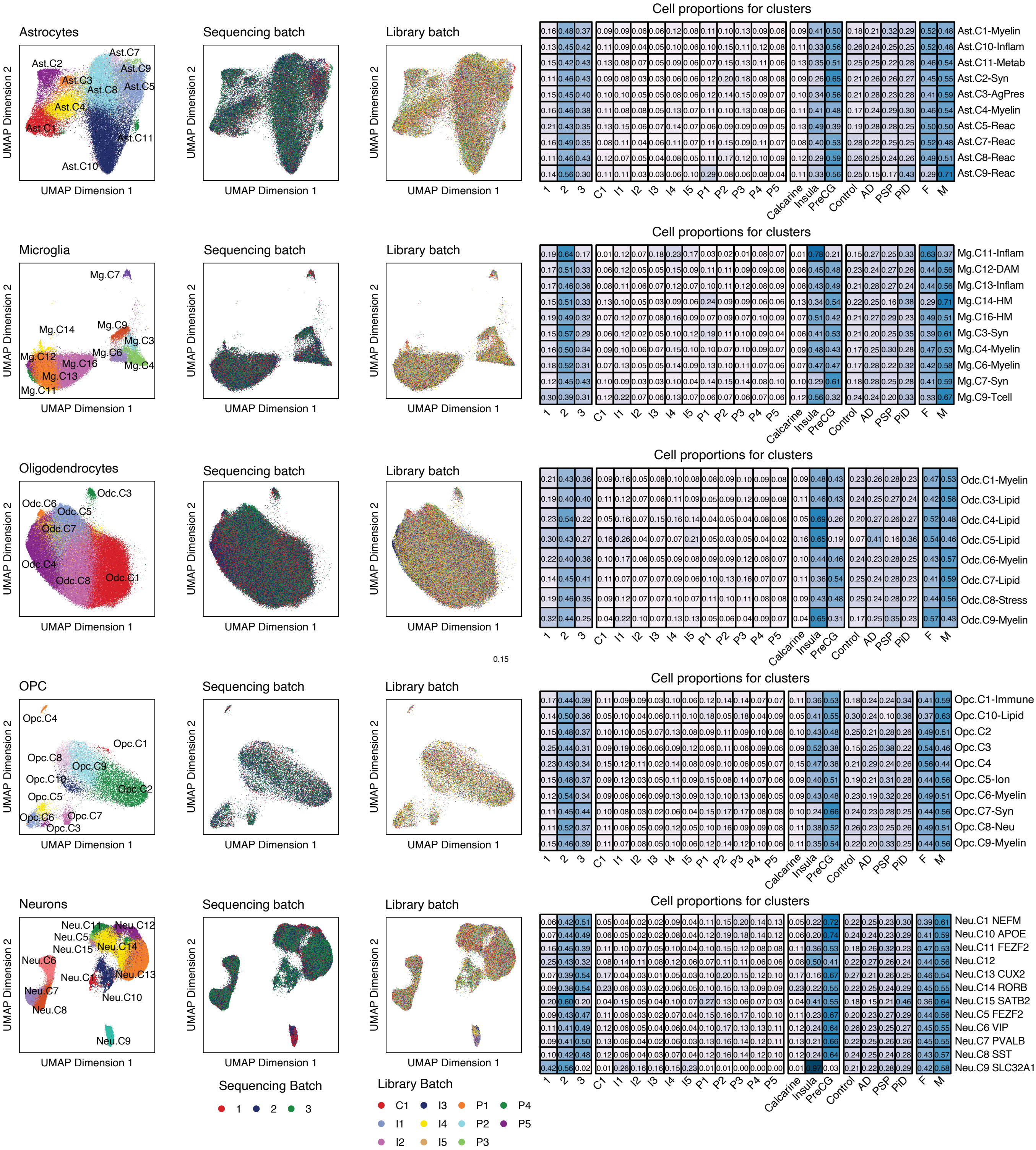

Supplementary Figure 15. Identified subclusters with negligible batch effects. UMAPs are colored by subclusters, sequencing batches, and library batches for each cell type. The proportions of cells per subcluster contributed by different batches, brain regions, conditions, and genders are displayed on the right.

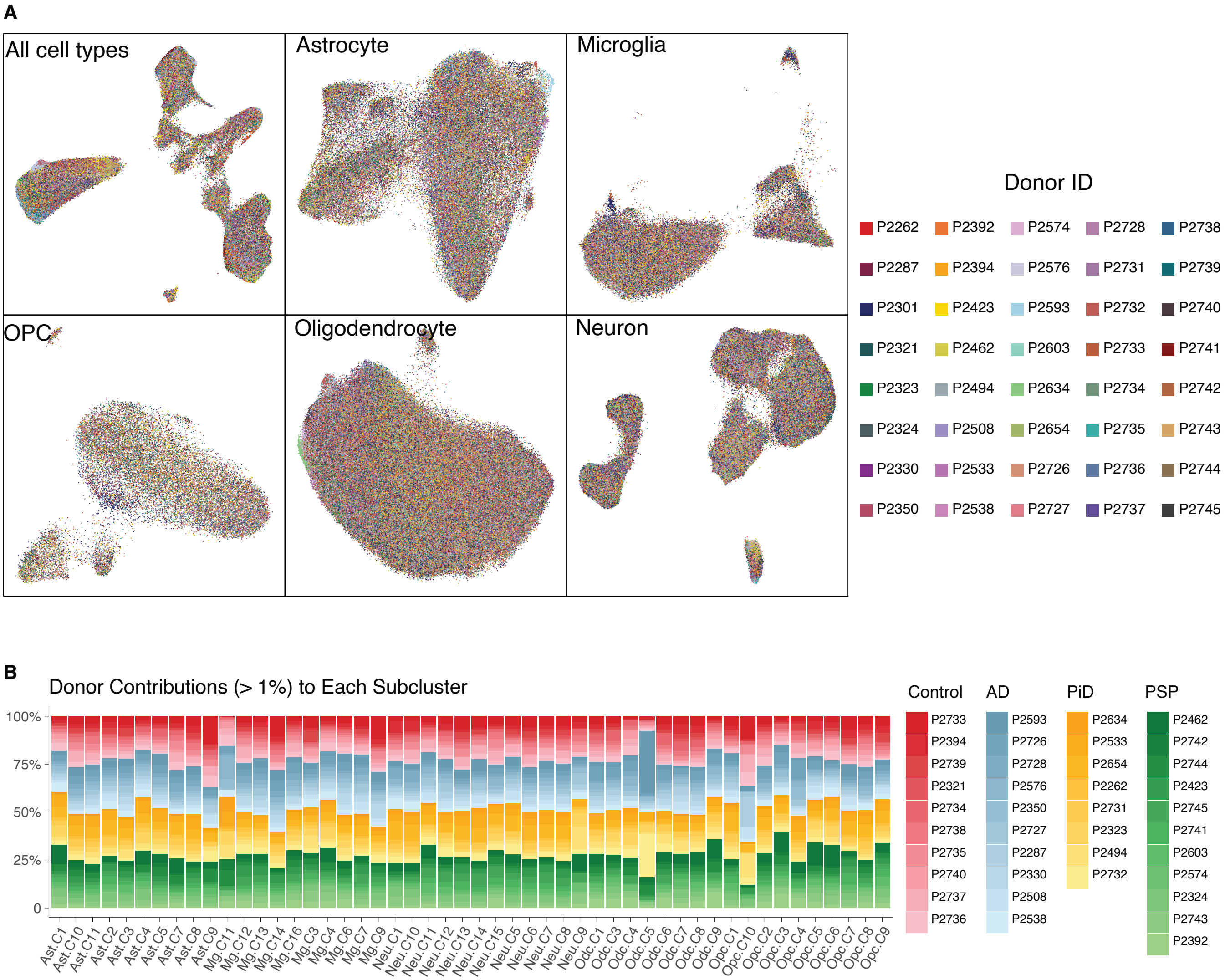

Supplementary Figure 16. Cluster distribution across donors.
(A) UMAPs for each cell type colored by donor.
(B) Percentage of cells in each cluster across donors.

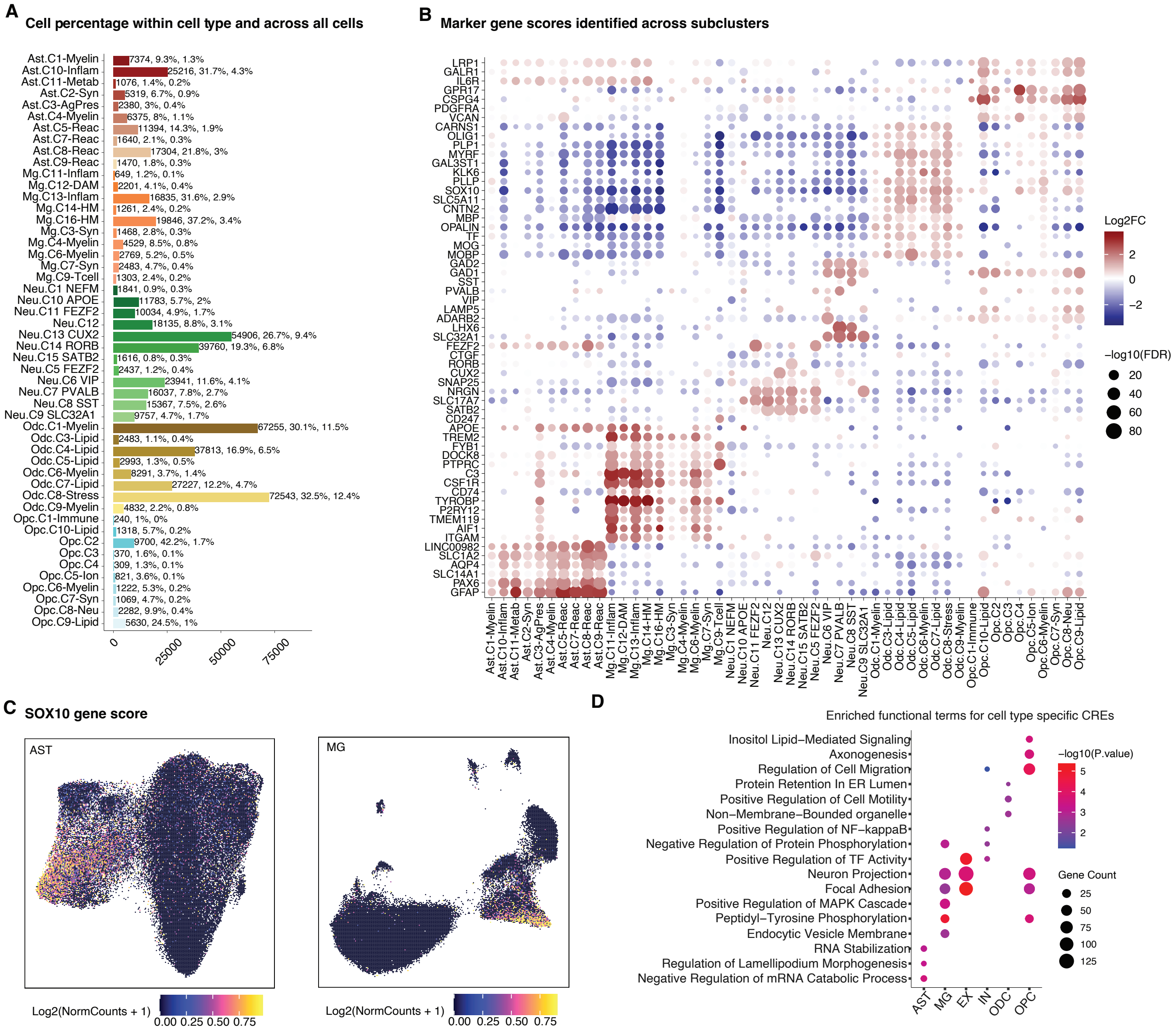

Supplementary Figure 17. Cell percentages and gene signatures of identified subclusters in human brain.
(A) Bar plot displaying the number and percentage for subclusters, labels representing cell number, subcluster frequency within the cell type, and overall subcluster frequency.
(B) Gene signatures of 50 subclusters, defined by marker gene scores compared across all subclusters.
(C) UMAPs showing subclusters and SOX10 gene scores in astrocytes and microglia.

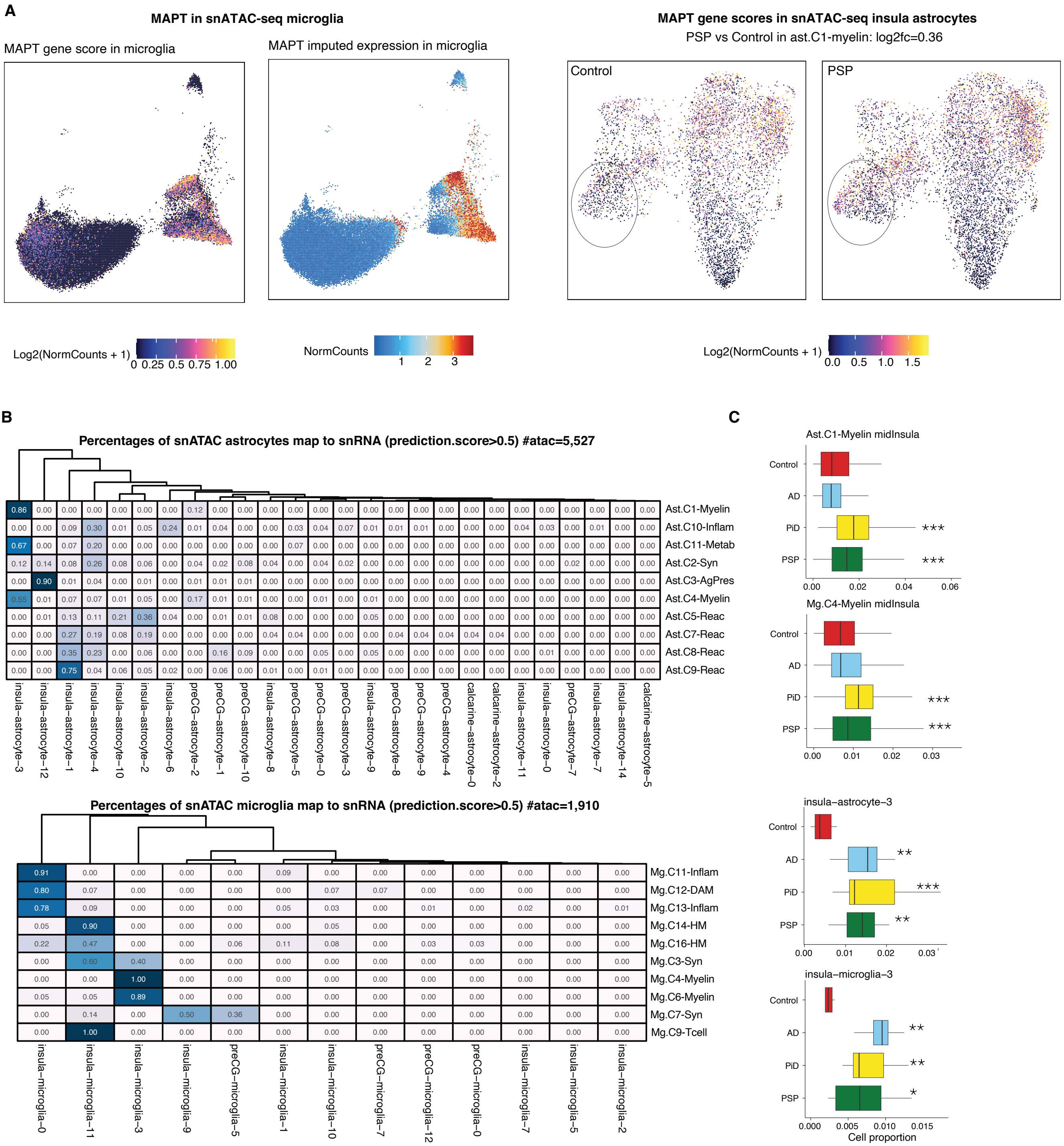

Supplementary Figure 18. Reproducing of cell proportion changes in ast.C1 and mg.C4.
(A) MAPT gene activity and imputed expression in microglia (top) and astrocytes (bottom) derived from snATAC-seq.
(B) Percentages of snATAC-seq astrocytes (top) and microglia (bottom) mapped to the pre-defined snRNA-seq subtypes. Cellular alignment was performed using Seurat CCA with prediction scores > 0.5.
(C) Boxplots showing cell composition in each condition for the snATAC-seq subclusters (top) and the mapped snRNA-seq subtypes (bottom). Composition differences between disease and control groups were assessed using the two-sided Wilcoxon rank-sum tests after bootstrapping 15 times, and P values were adjusted using the Benjamini-Hochberg false discovery rate method. FDR thresholds: *< 0.05; ** <0.005; *** < 0.001.

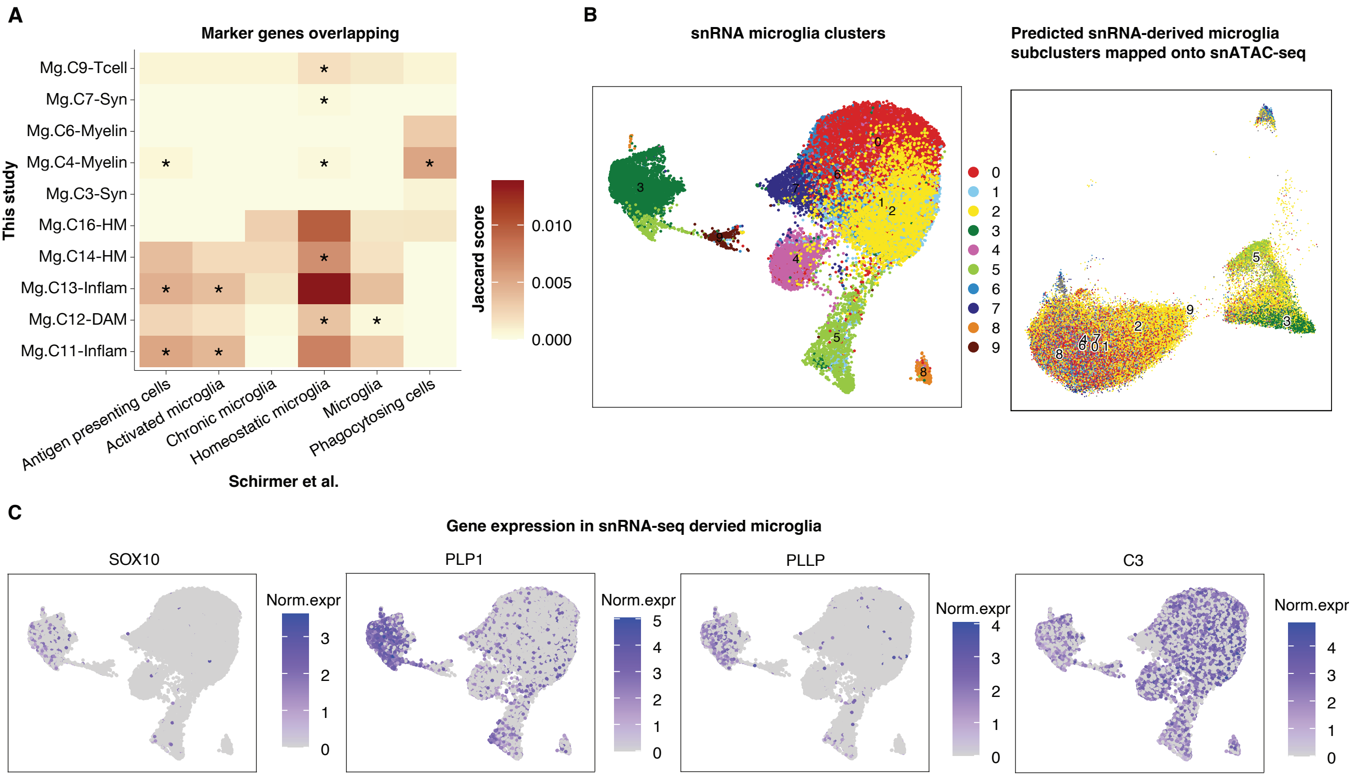

Supplementary Figure 19. Microglia Subtypes alignment with sn-RNAseq studies.

(A) Overlap of marker genes with microglia subtypes from Schirmer et al 54 by Jaccard score and two-sided Fisher's exact tests. and P values were adjusted using the Benjamini–Hochberg false discovery rate method. Significant overlaps with FDR-adjusted p value < 0.1 are marked with asterisks.

(B) UMAP plots of microglia subclusters profiled in snRNA-seq (top left) and snATAC-seq (top right).

(C) UMAPs display selected marker genes expressed in snRNA-seq-derived microglia subclusters.

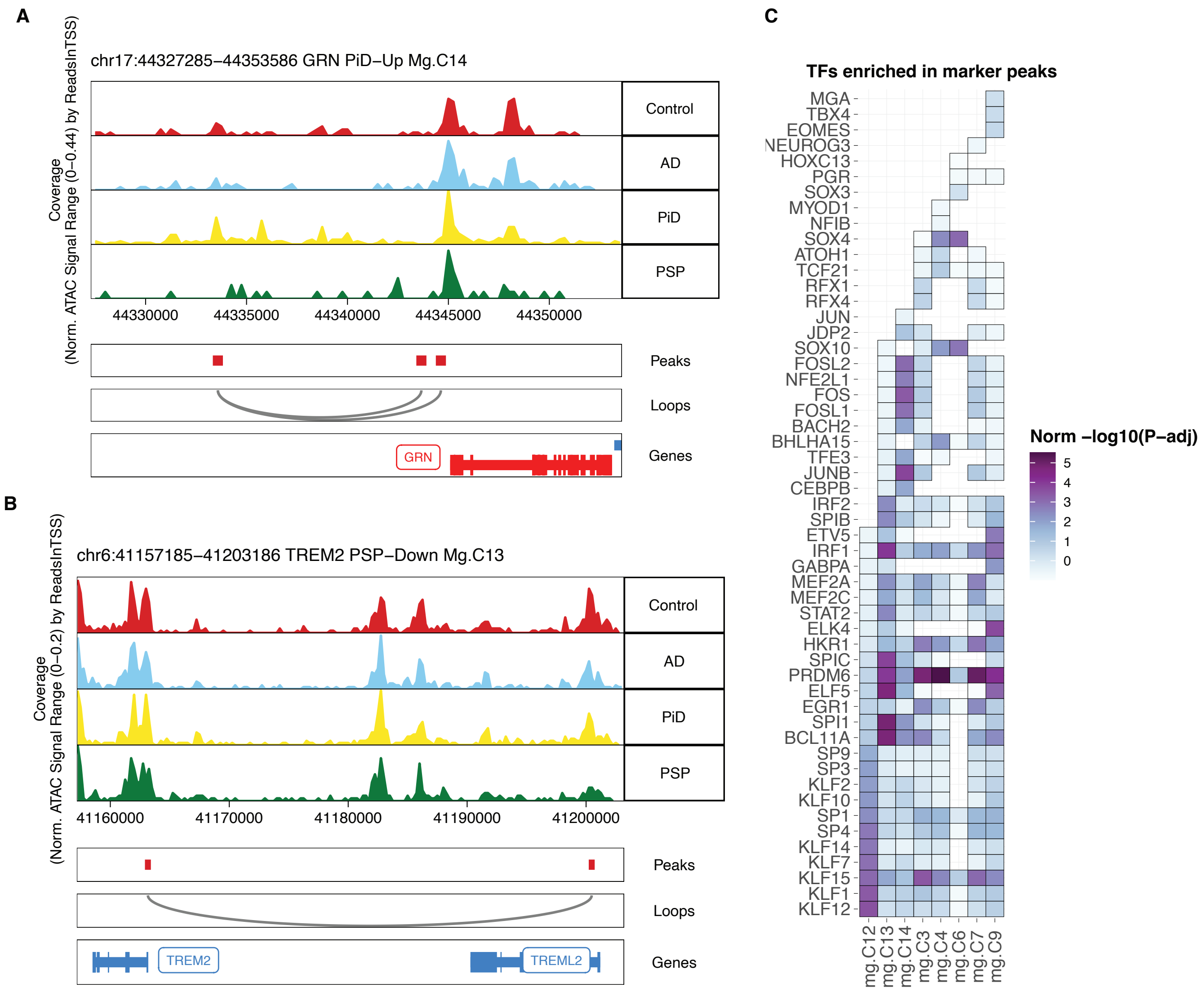

Supplementary Figure 20. Disease-specific enhancer accessibility and TF motif enrichment in microglial subtypes.
(A) Genome browser tracks illustrating differential enhancer accessibility across disorders, showing PiD-specific activation of the GRN enhancer in mg.C14.
(B) Genome browser tracks showing reduced accessibility of the TREM2 enhancer in PSP within mg.C13.
(C) Motif enrichment analysis of subtype-defining peaks performed using MEME. Normalized false discovery rate (FDR) values are shown for enriched transcription factors (TFs) in each microglial subtype.

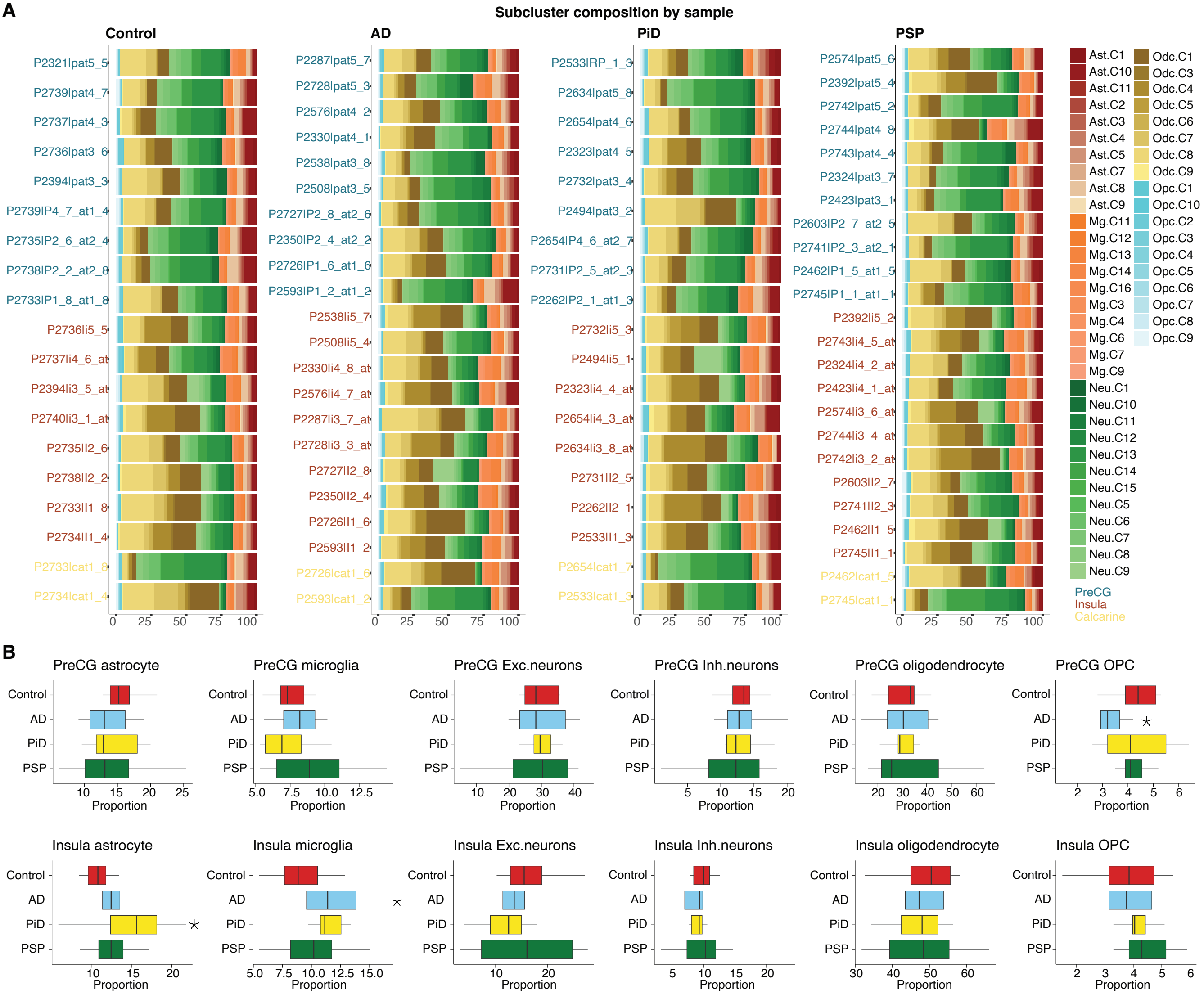

Supplementary Figure 21. Cell distribution across subclusters in samples and changes in cell type composition.
(A) Distribution of subclusters across samples, split by disease, with sample IDs colored by brain regions
(B) Boxplots displaying the relative abundance of each cell type across conditions. Changes in cell composition between disease and control were tested using the two-sided linear regression models via limma package, adjusting for age and PMI. *P < 0.05.

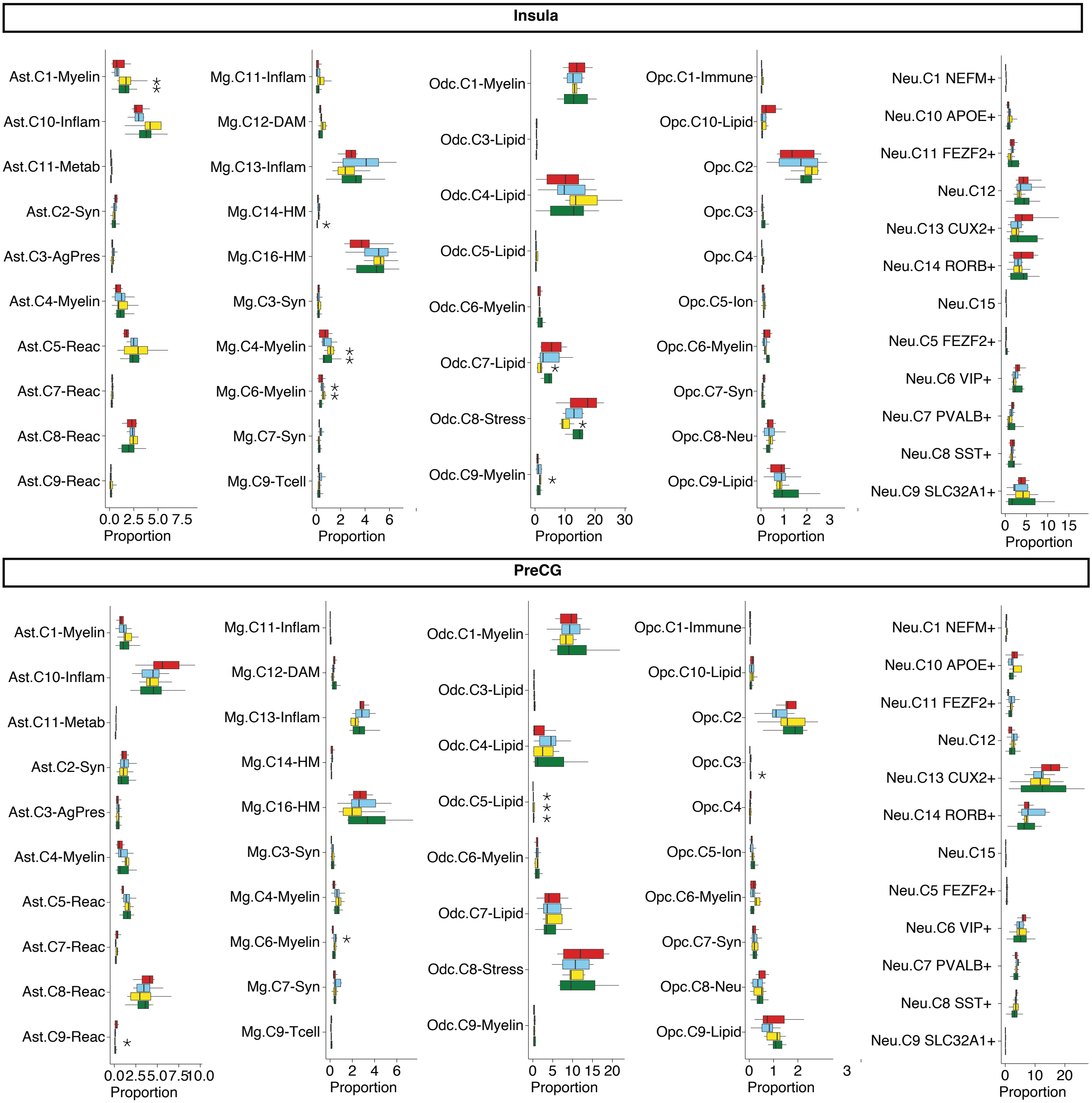

Supplementary Figure 22. Subcluster composition changes in disease vs. control.
Boxplots displaying the relative abundance of each subcluster across conditions. Changes in cell composition between disease and control were tested using two-sided linear regression mocel via limma package, adjusting for age and PMI. *P < 0.05.

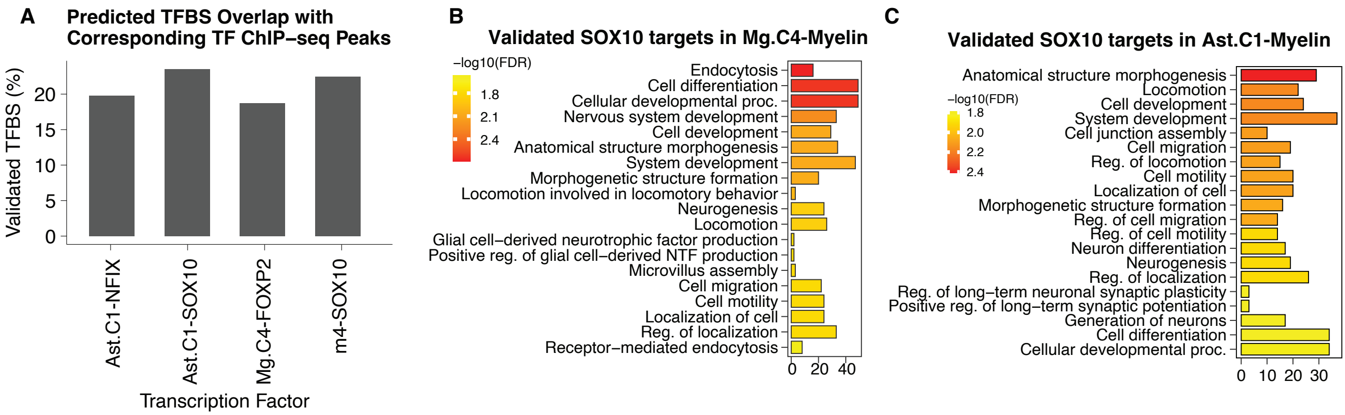

Supplementary Figure 23. TF binding sites verified using TF ChIP-Seq data.
(A) Comparison of predicted transcription factor binding sites (TFBSs) in ast.C1 and mg.C4 with TF ChIP-seq data.
(B-C) Functional enrichment of validated TF target genes in mg.C4 (B) and ast.C1 (C). TF ChIP-seq datasets for SOX10, NFIX, and FOXP2 were obtained from the Gene Transcription Regulation Database (GTRD) (experiment IDs: EXP034107, EXP038549, and EXP010606).

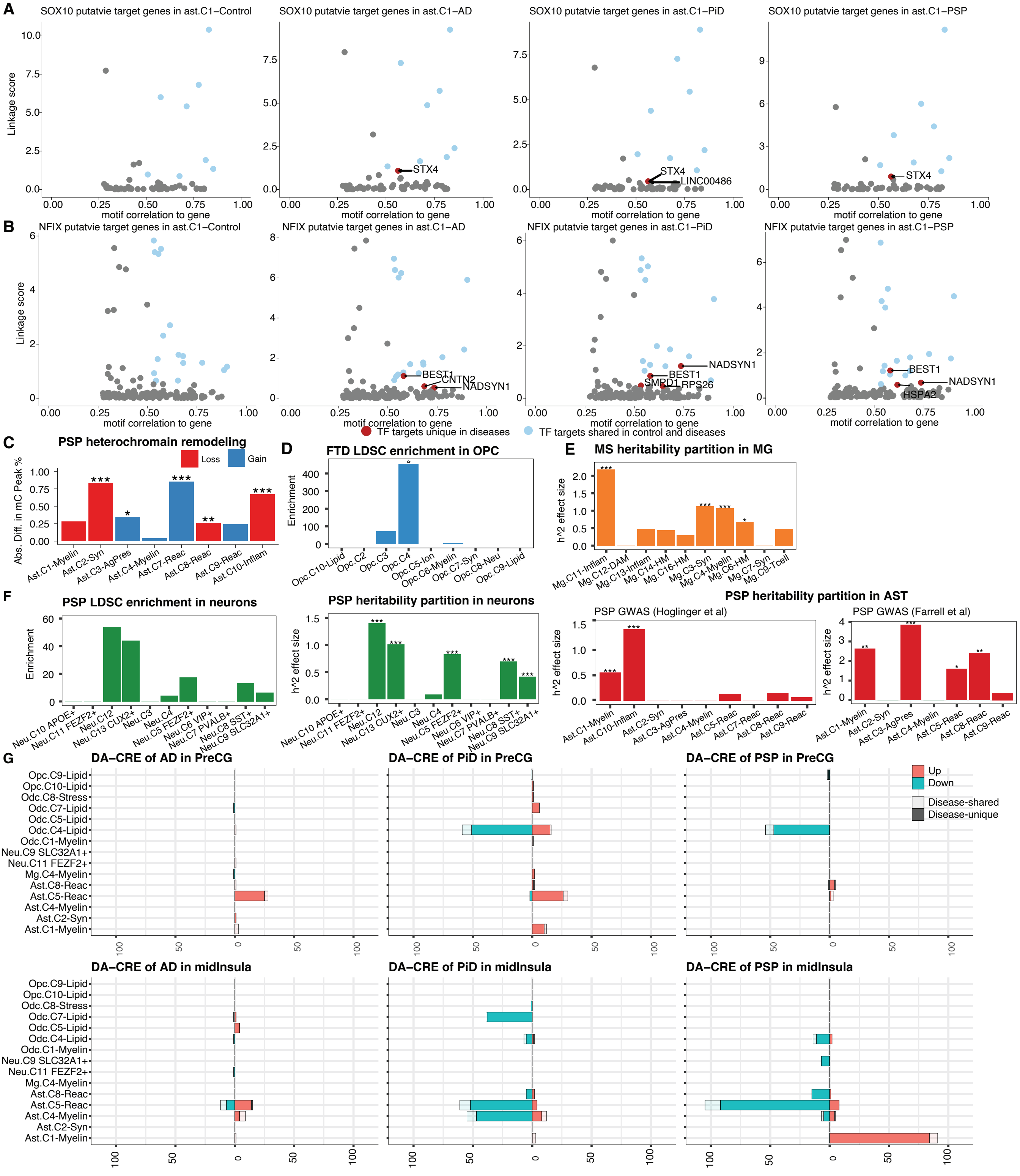

Supplementary Figure 24. Disease heritability partition by LDSC in subcluster-specific peaks.
(A-B) Target genes SOX10 (A) and NFIX (B) under different conditions in ast.C1. The x-axis represents the Pearson correlation between motif activity and gene activity, and the y-axis indicates gene-TF linkage scores.

(C) Bar plot showing astrocyte-specific heterochromatin remodeling in PSP. The values represent the percentage of ATAC peaks annotated as hypermethylated (mC%) and the absolute difference between PSP and control. A higher mC% in PSP indicates a loss of heterochromatin, while a lower mC% indicates a gain of heterochromatin. Methylation was quantified using single-nucleus methyl-3C sequencing data 76 with ALLCools 77. Empirical P-values were obtained from a two-sided permutation test (n = 10,000) and adjusted for multiple testing using the Benjamini-Hochberg false discovery rate method
(*** FDR ≤ 0.001, ** FDR ≤ 0.01, * 0.01 < FDR ≤ 0.05).
(D) FTD GWAS heritability partition in OPC subcluster-specific peaks measured by LDSC enrichment.
(E-F) GWAS heritability partition of MS in microglia (E) and PSP (F) in neuron and astrocyte subcluster-specific peaks, using both LDSC standardized effect size and enrichment metrics. (significant for τ_c^*, FDR *< 0.05; ** <0.005; *** < 0.001).
(G) Number of DA-CREs detected at the subcluster level. Bar plots show the distribution of DA-CREs for each disease in each subcluster, with upregulated DA-CREs in red and downregulated DA-CREs in green. The significant cutoff was set at |log2fc| >= 1.2 and FDR< 0.1. Ast, astrocytes; neu, neurons; MG, microglia; ODC, oligodendrocytes.

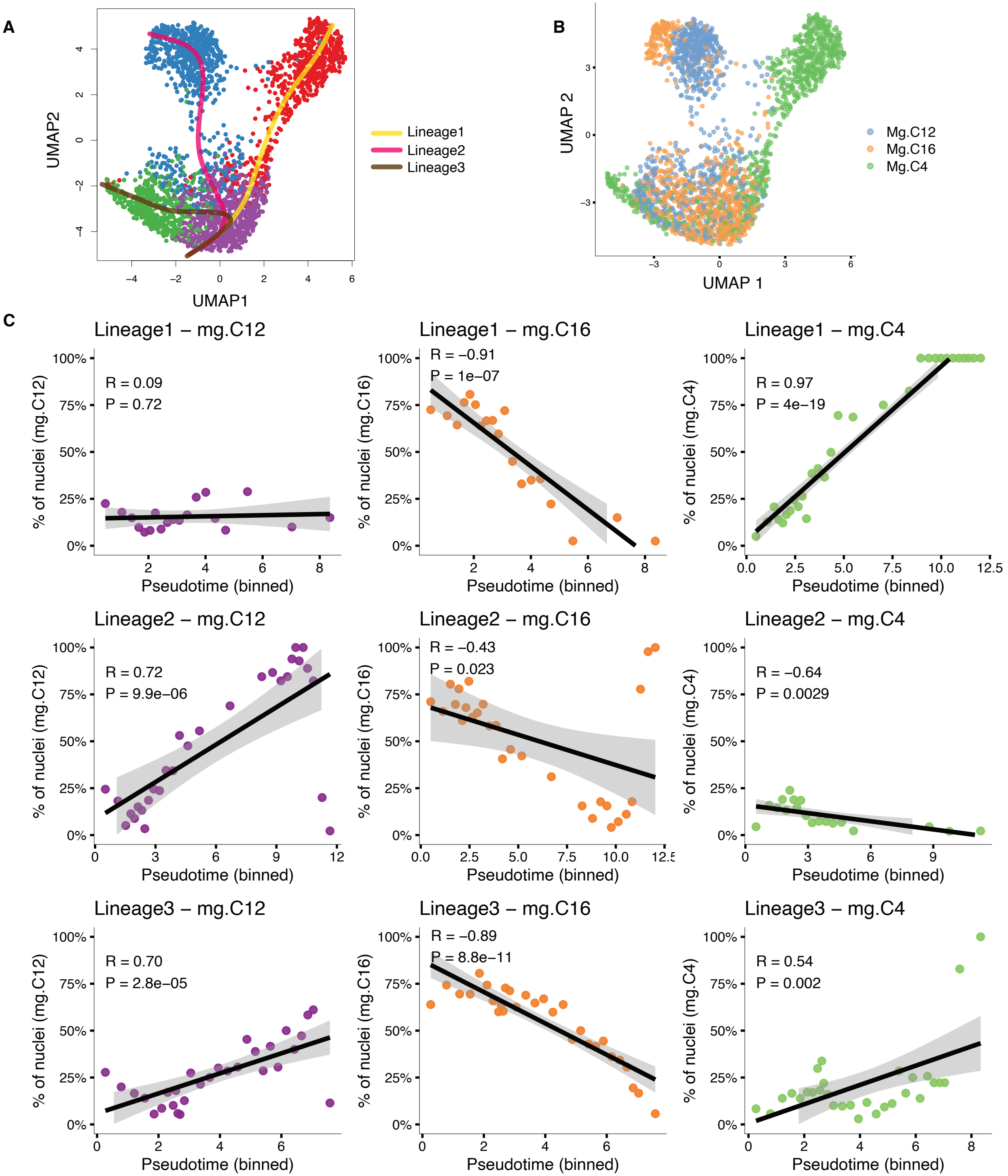

Supplementary Figure 25. Slingshot pseudotime trajectories of microglial subtypes in the insula.
(A) UMAP embedding showing Slingshot-inferred pseudotime trajectories for homeostatic mg.C16, PiD-associated mg.C4, and DAM-associated mg.C12, colored by lineage assignment.
(B) The same UMAP colored by microglial subtype identity.
(C) Dot plot showing Pearson correlations between pseudotime values and microglial subtype abundance across the three inferred lineages.

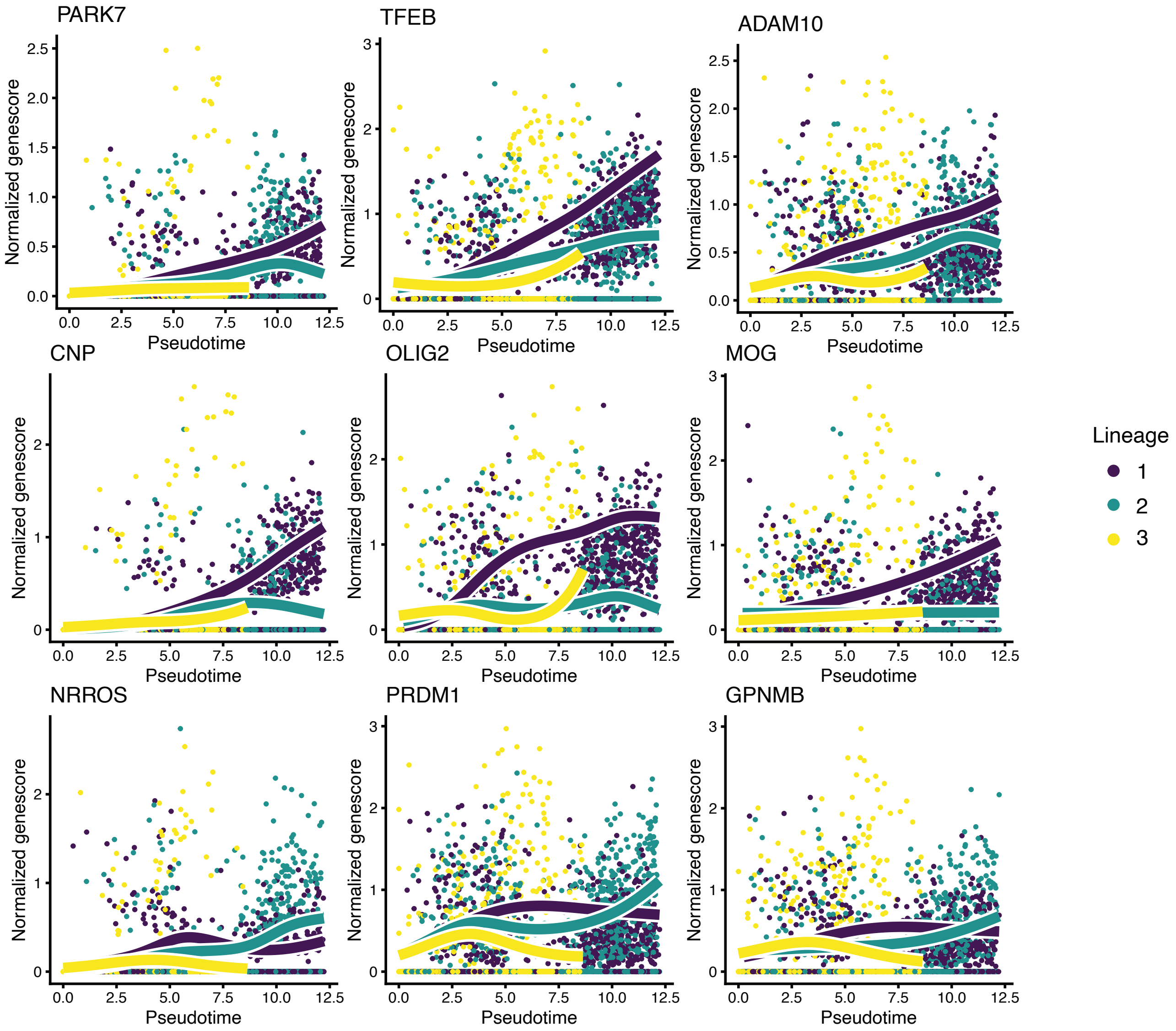

Supplementary Figure 26. Gene activity along pseudotime trajectories of microglial subtypes in the insula.
Slingshot trajectories were inferred for homeostatic mg.C16, PiD-associated mg.C4, and DAM-associated mg.C12. Points represent individual cells, colored by pseudotime lineage assignment. Normalized gene activity scores across along pseudotime were modeled using generalized additive models (GAM; y ~ s(pseudotime)) for each gene-lineage combination.

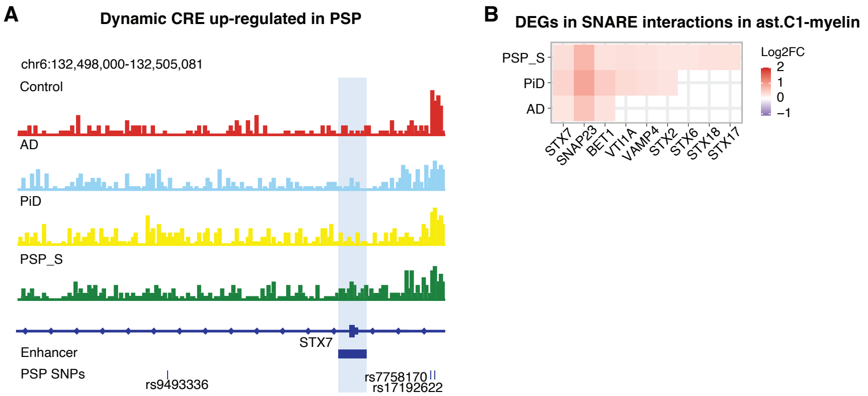

Supplementary Figure 27. SNARE interaction involved in PSP ast.C1.
(A) Chromatin accessibility track illustrating a PSP-upregulated STX7 enhancer near PSP-associated variants.
(B) Differentially expressed genes (FDR-adjusted p value <0.1) in ast.C1 involved in SNARE interactions.

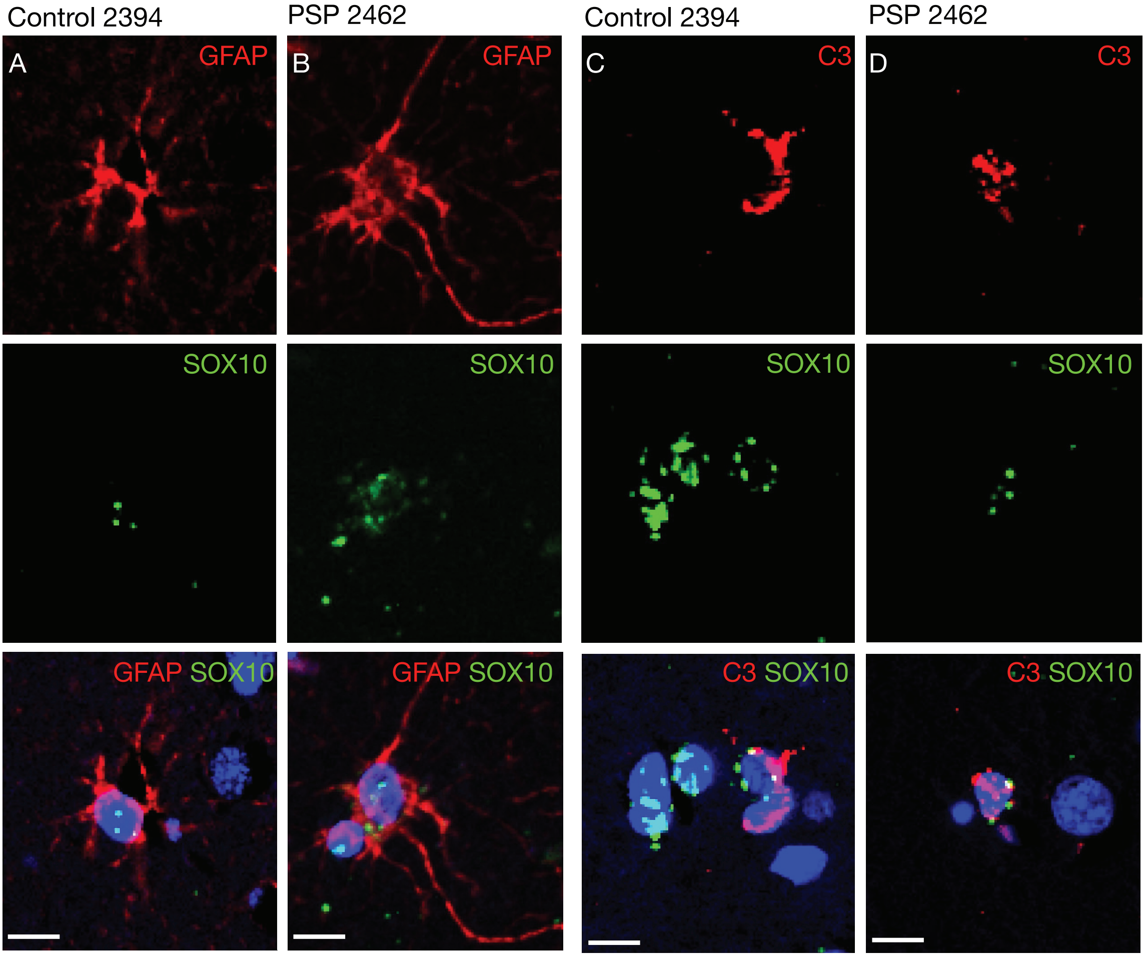

E Percentage of SOX10+ microglia and astrocyte

	SOX10,C3/C3	SOX10, GFAP/GFAP
Control	19.0	29.2
PSP	26.9	42.6

F

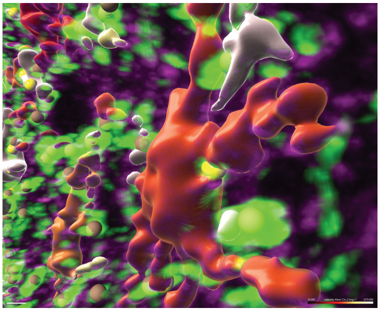

G

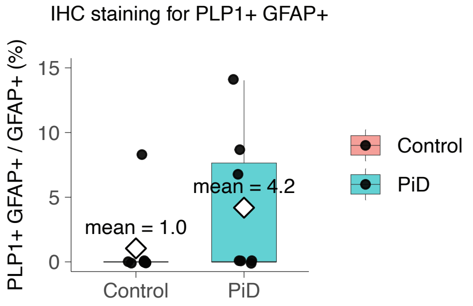

Supplementary Figure 28. RNAscope and IHC validation of myelin-associated glia in tauopathies.
(A-B) RNAscope imaging of SOX10 and IHC of GFAP in control and PSP insula tissue. (C-D) RNAscope imaging of C3 and SOX10 in control and PSP. GFAP and C3 signals are shown in red; SOX10 is shown in green. Scale bar: 10 μm. (E) Quantification table summarizing the average percentage of $SOX10^{+}$ astrocytes and $SOX10^{+}$ microglia per slide, based on six randomly selected regions (> 60 astrocytes and > 100 microglial cells). (F) High-magnification 3D confocal IHC reconstruction demonstrating close spatial association between PLP1 (green) and GFAP (red and grey). Red GFAP represents a closer proximity to PLP1, as compared to grey GFAP. Spot-distance analysis in Imaris was used to quantify PLP1-GFAP proximity, with colocalized yellow puncta representing Imaris standards of colocalization defined by a distance threshold of -3 to -1 μm. (G) Quantification of $PLP1^{+}$ astrocyte percentages in control (n=8) and PiD (n=7) samples.

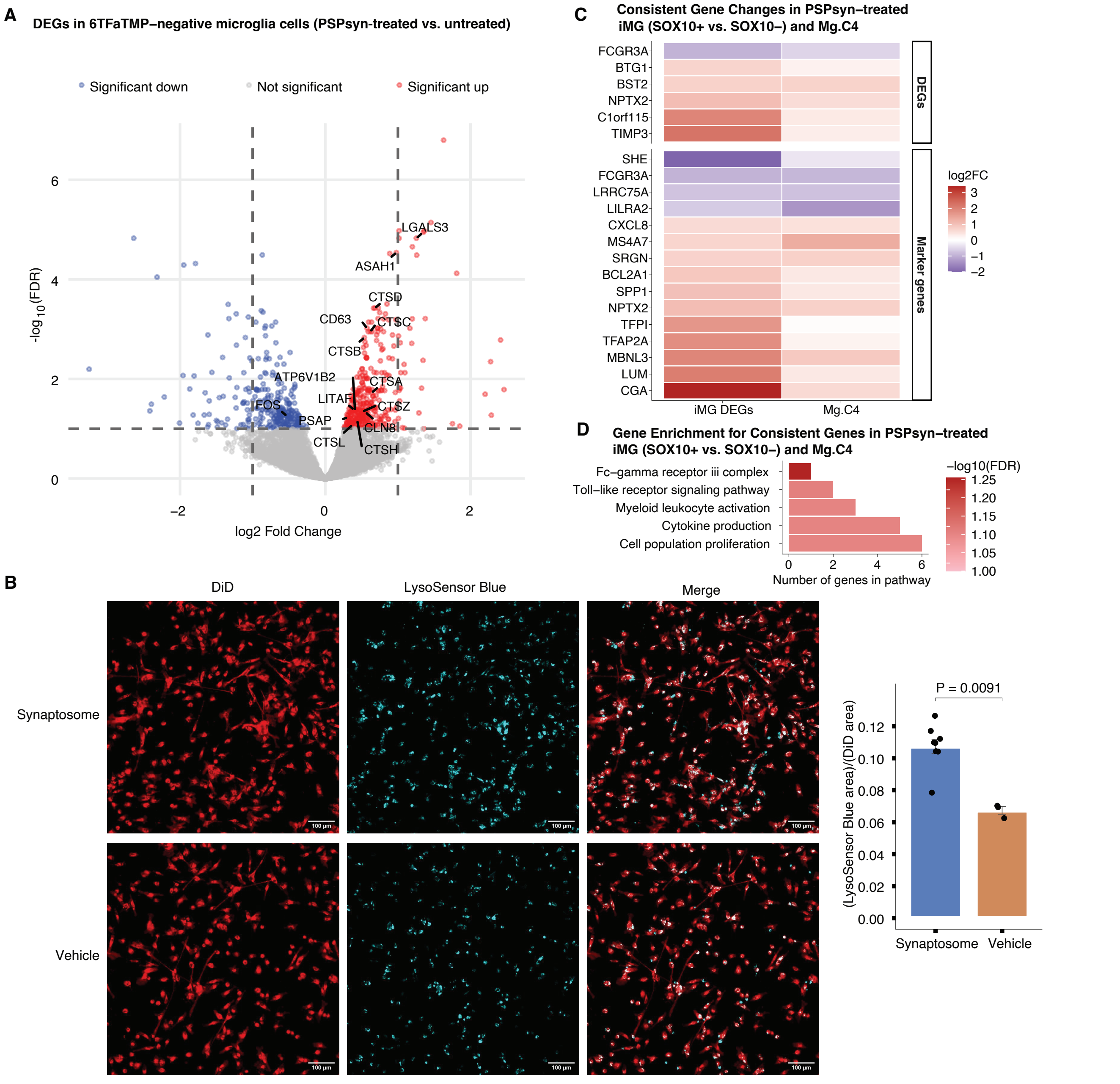

Supplementary Figure 29. CRISPR activation of SOX10 in iPSC-derived microglia (iMG) under stress induces differential gene expression (DGE) resembling gene programs of myelin-related microglia in the human brain.

(A) Differential expression analysis comparing 6TFaTMP-negative iPSC-derived microglia treated with PSP synaptosomes to untreated controls. SOX10 induction was observed following PSP synaptosome treatment, with higher log-normalized expression compared to untreated samples (1.67 vs 0.31; mean, n = 2 per group).

(B) Live-cell confocal microscope images of 6TFa iPSC-derived microglia treated with either 20µg/mL of disease synaptosomes or 0.32M sucrose with protease inhibitors (vehicle control) and stained with the lipophilic dialkylcarbocyanine dye DiD (red), which dyes all lipid membranes, and LysoSensor Blue DND-167 (cyan), which fluoresces brightly in acidic organelles, especially lysosomes. Quantification of the fluorescence was calculated by taking the area of fluorescence of LysoSensor Blue within the area of fluorescence of DiD and dividing by the area of fluorescence of DiD. This represents the proportion of cellular area occupied by active lysosomes, which can be interpreted as degree of lysosome activation. 9 images of disease synaptosome-treated cells and 3 images of 0.32M sucrose-treated cells were compared by two-sided Wilcoxon rank-sum tests.

(C) The heatmap shows consistent changes in SOX10-induced DGE in iMG under stress and in myelin-related microglial markers.

(D) Functional enrichment of genes show consistent profiles in (C).
