# Supplementary material for: Single-nucleus epigenomic dysregulation unmasks genetic risk-associated neurodegenerative glia states
Source: Nat Commun. 2026 May 14;17:6439. doi: 10.1038/s41467-026-73007-1 (PMC13376791; doi:10.1038/s41467-026-73007-1)
Supplement: Supplementary file 3 — Reporting Summary [file 41467_2026_73007_MOESM3_ESM.pdf]

nature portfolio

Corresponding author(s): Jessica E. Rexach

Last updated by author(s): 2026/03/11

Reporting Summary

Nature Portfolio wishes to improve the reproducibility of the work that we publish. This form provides structure for consistency and transparency in reporting. For further information on Nature Portfolio policies, see our Editorial Policies and the Editorial Policy Checklist.

Statistics

For all statistical analyses, confirm that the following items are present in the figure legend, table legend, main text, or Methods section.

n/a	Confirmed	
☐	☒	The exact sample size (*n*) for each experimental group/condition, given as a discrete number and unit of measurement
☐	☒	A statement on whether measurements were taken from distinct samples or whether the same sample was measured repeatedly
☐	☒	The statistical test(s) used AND whether they are one- or two-sided<br>*Only common tests should be described solely by name; describe more complex techniques in the Methods section.*
☐	☒	A description of all covariates tested
☐	☒	A description of any assumptions or corrections, such as tests of normality and adjustment for multiple comparisons
☐	☒	A full description of the statistical parameters including central tendency (e.g. means) or other basic estimates (e.g. regression coefficient) AND variation (e.g. standard deviation) or associated estimates of uncertainty (e.g. confidence intervals)
☐	☒	For null hypothesis testing, the test statistic (e.g. *F*, *t*, *r*) with confidence intervals, effect sizes, degrees of freedom and *P* value noted<br>*Give P values as exact values whenever suitable.*
☒	☐	For Bayesian analysis, information on the choice of priors and Markov chain Monte Carlo settings
☒	☐	For hierarchical and complex designs, identification of the appropriate level for tests and full reporting of outcomes
☐	☒	Estimates of effect sizes (e.g. Cohen's *d*, Pearson's *r*), indicating how they were calculated

Our web collection on statistics for biologists contains articles on many of the points above.

Software and code

Policy information about availability of computer code

Data collection	Immunofluorescence and RNAscope images were acquired using the Leica Biosystems platform. Manual immuniflurescence images were captured via Vectra Polaris microscope. cDNA libraries were generated with 10x Genomics and sequenced on the Illumina NovaSeq 6000 system. Bulk RNA-sequencing data was generated and processed by Plasmidsaurus using Oxford Nanopore Technology.
Data analysis	Cell Ranger ATAC Single-Cell Software Suite (v 2.1.0) was used for demultiplexing, barcode assignment, read mapping and UMI quantification. All downstream analyses were performed using R, Python, and shell scripts. The following packages were used for computation analyses: ArchR (v 1.0.3), Seurat (v 5.1.0), ALLCools (v 1.0.5), Limma (v 3.54.2), LDSC (v 1.0.1), Samtools (v 1.21), plink (v 1.90), Scanpy (v 1.9.8), ChromVar (v 1.20.2), TOBIAS (v 0.17.1), CellphoneDB (v 4.0.0), Signac (v 1.11.9000), enrichR (v 3.4), ShinyGO (v 0.80), MEME Suite (v 1.6.0).<br><br>Phenochart v2.2.0 and Imaris 10.1 were used for RNAscope and immunofluorescence images.

For manuscripts utilizing custom algorithms or software that are central to the research but not yet described in published literature, software must be made available to editors and reviewers. We strongly encourage code deposition in a community repository (e.g. GitHub). See the Nature Portfolio guidelines for submitting code & software for further information.

Data

Policy information about availability of data

All manuscripts must include a data availability statement. This statement should provide the following information, where applicable:

- Accession codes, unique identifiers, or web links for publicly available datasets
- A description of any restrictions on data availability
- For clinical datasets or third party data, please ensure that the statement adheres to our policy

Raw data from single nuclear RNAseq and ATACseq have been deposited in Synapse (syn52369053) and are available at https://www.synapse.org/Synapse:syn52369053. All original code generated in this study is available on GitHub (https://github.com/rexachgroup/pci_snATAC) and archived at Zenodo (https://doi.org/10.5281/zenodo.18904265)

Research involving human participants, their data, or biological material

Policy information about studies with human participants or human data. See also policy information about sex, gender (identity/presentation), and sexual orientation and race, ethnicity and racism.

Reporting on sex and gender	Sex was considered as a biological variable in the study design. For human snRNA-seq and snATAC-seq datasets, donor sex (female and male) was incorporated into sample selection and recorded in metadata, and samples were selected to be as balanced as possible by sex within the constraints of tissue availability. However, due to limited and uneven sample sizes within individual disease groups, formal sex-stratified statistical analyses were not performed. All cell line–based experiments were conducted using male-derived iPSC lines.
Reporting on race, ethnicity, or other socially relevant groupings	Information on race and ethnicity was not available and therefore was not included in the data analysis.
Population characteristics	For this study, we selected 41 participants including 10 subjects with clinical diagnosis of bvFTD and neuropathological diagnosis of Pick's disease (FTLD-tau), 10 subjects with clinical diagnoses of AD-type dementia and a neuropathological diagnosis of Alzheimer's disease, and 11 subjects with a clinical diagnosis of PSP-RS and a neuropathological diagnosis of PSP (FTLD-tau), and 10 non-demented controls, sex-matched to the patients. Freshly frozen human brain tissue (BA4: precentral gyrus, V1: calcarine cortex, INS: insular cortex) were obtained from the UCSF Neurodegenerative Disease Brain Bank and University of Pennsylvania Center for Neurodegenerative Disease Research Brain.
Recruitment	All procedures involving the use of postmortem human brain were conducted after obtaining the written informed consent, and after approval by the UCSF Committee on Human Research and the University of Pennsylvania Institutional Review Board. IRB exemption was obtained from the UCLA IRB to authorize use of de-identified human postmortem brain single nuclear sequencing data in this study. Neuropathological diagnoses were made prospectively at the contributing brain banks following standard criteria.
Ethics oversight	This study used de-identified postmortem human brain tissue obtained from the University of Pennsylvania Center for Neurodegenerative Disease Research Brain Bank and the UCSF Neurodegenerative Disease Brain Bank. Tissue collection and distribution by these brain banks were conducted under Institutional Review Board–approved protocols with informed consent from donors or their legal representatives. All specimens used in this study were de-identified and derived exclusively from deceased individuals. In accordance with U.S. federal regulations governing human subjects research (45 CFR 46), this work does not constitute human subjects research; therefore, Institutional Review Board review was not required.

Note that full information on the approval of the study protocol must also be provided in the manuscript.

Field-specific reporting

Please select the one below that is the best fit for your research. If you are not sure, read the appropriate sections before making your selection.

☒ Life sciences ☐ Behavioural & social sciences ☐ Ecological, evolutionary & environmental sciences

For a reference copy of the document with all sections, see nature.com/documents/nr-reporting-summary-flat.pdf

Life sciences study design

All studies must disclose on these points even when the disclosure is negative.

Sample size	Single-nucleus RNA-seq and ATAC-seq were performed on samples from 41 participants (Discovery cohort) to capture regional tauopathy diversity and provide sufficient statistical power for detecting changes in cell-type proportions and associations with disease traits.
Data exclusions	We excluded one donor from downstream analysis due to inconsistent clinical diagnosis and pathological presentation.

Replication	Our findings on disease heritability, based on LDSC applied to disease-dynamic peaks, were replicated in the SEA-AD dataset comprising 84 participants profiled by single-nucleus ATAC-seq.
Randomization	Each library construction batch consisted of four samples and was balanced across disease groups.
Blinding	Investigators were blinded to the pathological and clinical information of participants during the data processing and experimental phases of snATAC-seq, snRNA-seq, immunohistochemistry, and RNAscope. This included steps such as quality control, clustering, cell type annotation, and image analysis.

Reporting for specific materials, systems and methods

We require information from authors about some types of materials, experimental systems and methods used in many studies. Here, indicate whether each material, system or method listed is relevant to your study. If you are not sure if a list item applies to your research, read the appropriate section before selecting a response.

Materials & experimental systems

n/a	Involved in the study	
☐	☒	Antibodies
☐	☒	Eukaryotic cell lines
☒	☐	Palaeontology and archaeology
☒	☐	Animals and other organisms
☒	☐	Clinical data
☒	☐	Dual use research of concern
☒	☐	Plants

Methods

n/a	Involved in the study	
☒	☐	ChIP-seq
☒	☐	Flow cytometry
☒	☐	MRI-based neuroimaging

Antibodies

Antibodies used	Anti-GFAP antibody (Dako, clone M0761, at a 1:200 dilution; and CATALOG #173308, at a 1:500 dilution)<br>Anti-PLP1 antibody (CST #28702) at a 1:150 dilution.<br>RNAscope probes: human SOX10 (CATALOG # 484128-C4) and PLP1 (CATALOG# 499278)
Validation	Antibody specificity has been confirmed on human post-mortem brain tissues. The specificity of the RNAscope probes was validated by ACD.

Eukaryotic cell lines

Policy information about cell lines and Sex and Gender in Research

Cell line source(s)	We used the 6TF microglia iPSC line developed in the Kampmann Lab at UCSF, derived from a male WTC11 iPSC background.
Authentication	Low-depth Whole Genome Sequencing (WGS)-based CNV analysis for pluripotent stem cells (PSCs). This approach allows us to detect chromosomal abnormalities performed by the UCLA Human Stem Cell & Genome Engineering Center.
Mycoplasma contamination	Cell lines tested negative for mycoplasma contamination.
Commonly misidentified lines (See ICLAC register)	*Name any commonly misidentified cell lines used in the study and provide a rationale for their use.*

Plants

Seed stocks	*Report on the source of all seed stocks or other plant material used. If applicable, state the seed stock centre and catalogue number. If plant specimens were collected from the field, describe the collection location, date and sampling procedures.*
Novel plant genotypes	*Describe the methods by which all novel plant genotypes were produced. This includes those generated by transgenic approaches, gene editing, chemical/radiation-based mutagenesis and hybridization. For transgenic lines, describe the transformation method, the number of independent lines analyzed and the generation upon which experiments were performed. For gene-edited lines, describe the editor used, the endogenous sequence targeted for editing, the targeting guide RNA sequence (if applicable) and how the editor was applied.*
Authentication	*Describe any authentication procedures for each seed stock used or novel genotype generated. Describe any experiments used to assess the effect of a mutation and, where applicable, how potential secondary effects (e.g. second site T-DNA insertions, mosiacism, off-target gene editing) were examined.*
