# Supplementary material for: Single-nucleus epigenomic dysregulation unmasks genetic risk-associated neurodegenerative glia states
Source: Nat Commun. 2026 May 14;17:6439. doi: 10.1038/s41467-026-73007-1 (PMC13376791; doi:10.1038/s41467-026-73007-1)
Supplement: Supplementary file 4 — Transparent Peer Review file [file 41467_2026_73007_MOESM4_ESM.pdf]

nature portfolio

Peer Review File

Single-nucleus epigenomic dysregulation unmasks genetic risk-associated neurodegenerative glia states

Corresponding Author: Dr Jessica Rexach

This file contains all reviewer reports in order by version, followed by all author rebuttals in order by version.

Version 0:

Reviewer comments:

Reviewer #1

(Remarks to the Author)
The authors profile chromatin accessibility (snATAC-seq) across Alzheimer's disease (AD), Pick's disease (PiD) and progressive supranuclear palsy (PSP), integrate the data with matched snRNA-seq, and survey cis-regulatory elements, transcription-factor activity, and GWAS enrichments. The MPRA is a particular strength of the study, although data-wise underexplored. While technically solid, methodologically done well and decorated with beautiful figures, virtually the entire dataset and many of the analytical pipelines were presented in their earlier study (Ref. 19). In this submission the authors largely revisit those data with incremental analyses; the narrative remains descriptive and no specific biological mechanism is functionally validated. Novelty is minimal, and there is redundancy with recent AD and PSP community resources describing altered microglial and astrocytic CRE environments. Results are reported as lists of subclusters, peaks, and GWAS enrichments, but there is no unifying hypothesis tested or mechanistic model proposed. The reader is left without a clear take-home message. The article may be better suited as a Data Resource with a short, focused extension on one of the many possible storyline threads (e.g. lysosomal pathway activation in PiD, FTLD microglia MPRA integration, or cell-cell interactions).

(Remarks on code availability)
The code is organized well, documented sufficiently and easy to read - this is a strength of the paper. Upon random interrogation of the R script files, I could not find any mistakes.

Reviewer #2

(Remarks to the Author)
Summary
This study employs integrated single-nucleus ATAC-seq and RNA-seq analyses of brain tissue from Alzheimer's disease (AD), Pick's disease (PiD), progressive supranuclear palsy (PSP), and controls across three brain regions. The authors focus on disease- and cell-type-specific chromatin accessibility dynamics, identify cis-regulatory elements (CREs), and analyze their functional relevance through integration with GWAS summary statistics, eQTLs, and massively parallel reporter assays (MPRAs). They highlight disorder-specific glial subtypes, particularly ast.C1 and mg.C4, as states associated with chromatin accessibility changes linked to disease heritability and potential resilience mechanisms. The manuscript reads well, however I would like to raise a few major and minor concerns before it is published.

Major Points
1. Use of Outdated PSP GWAS Summary Statistics. The authors rely on a 2011 PSP GWAS (Höglinger et al.), despite the existence of more recent studies with larger cohorts and better-powered variant discovery (e.g., Wang et al., Mol Neurodegener 2024, Farrell et al 2024 Nature communications). This is particularly important since newer studies report variants not captured in earlier datasets, potentially altering interpretations of heritability enrichment and regulatory targeting in PSP. Analyses should be updated with recent summary statistics or at minimum benchmarked against them.
2.Justification for Excluding Calcarine Cortex. The exclusion of calcarine cortex samples is stated to be due to "limited and variable" data, but this lacks sufficient justification. A more quantitative rationale is needed—e.g., cell yield, TSS enrichment, or clustering inconsistencies—and ideally visualized in supplementary figures to demonstrate that this region was unusable or inconsistent across samples.
3.No Reported Quantification of Pathology in Analyzed Hemispheres. The manuscript lacks explicit data on pathology

burden in the sampled hemispheres. While the contralateral hemisphere may be used for fixed tissue pathology, this should be clearly described. Quantitative pathology scoring (e.g., tau, amyloid, TDP-43, alpha-synuclein) for each brain region and sample should be provided, ideally in a supplementary table or figure, to confirm diagnostic classification and rule out comorbid pathology.
4.No Discussion or Adjustment for Clinical Heterogeneity. The study does not mention antemortem clinical features, disease duration, symptom severity, or cognitive assessments, which are highly relevant for stratifying molecular data. Including these clinical parameters and adjusting for them where possible (or explaining why they were not considered) would strengthen the interpretation of disease-state-specific findings.
5.Lack of Validation for Identified Glial Subpopulations. While ast.C1 and mg.C4 are emphasized as disease-associated states, external validation is minimal. A more robust demonstration—such as immunostaining for PLP1 or SOX10 in multiple samples, co-labeling with disease markers, or external dataset replication—would strengthen the claim that these are conserved and disease-relevant glial subtypes.
6.Potential Circularity in Cell-State Definition and Enrichment Testing. The same dataset is used to define subclusters, assign chromatin accessibility, and test GWAS heritability enrichment. While the authors attempt replication using SEA-AD data, more detail is needed about how independent the replication is. For example, are subclusters defined de novo in SEA-AD, or mapped from the discovery dataset? Explicit steps and metrics for validation across datasets should be described.
7. Methodological Clarity Around Tool Usage and Parameter Justification.
-ArchR: The use of ArchR for clustering, co-accessibility, and differential testing is appropriate, but more information is needed on parameter choices, including the justification for thresholds used (e.g., $\log_2 FC > 1.25$, $p < 0.001$ for DARs).
-S-LDSC (Stratified LD Score Regression): The manuscript lacks details about the annotation models used, whether the baseline model was included, and how multiple testing correction was applied across peak sets.
-ChromVAR: Variability analyses using ChromVAR are central to interpreting TF dynamics but are presented briefly. Were the motif databases manually curated for cell type–specific relevance? Clarify whether motif enrichment was adjusted for GC content or background accessibility.
-MPRA: The study integrates MPRA results from human microglial cell lines, but it is unclear how well these lines replicate the chromatin state of in vivo microglia, especially disease-associated subtypes like mg.C4. Were chromatin marks (e.g., H3K27ac) used to validate MPRA constructs? Were control (non-regulatory) SNPs tested for specificity?
8. Unclear Extent of Batch Correction and Donor Contribution Control. While the authors mention using Harmony for batch correction, additional information is needed on how donor-level effects (e.g., sample imbalance, individual heterogeneity) were handled in clustering, differential testing, and enrichment analyses. Were mixed-effect models used? How many donors contributed to each subcluster?
9.Conclusion Section Lacks Focus and Needs Structural Improvement. The discussion, particularly the final paragraphs, is lengthy and repetitive, which dilutes the impact of the key findings. A more structured conclusion that clearly outlines (a) major biological discoveries, (b) translational implications, and (c) limitations/future directions would significantly improve readability.

Minor Points and Suggestions
1.Include Figure of Pathology Burden. Add a supplemental table with pathology scores by region and subject (e.g., Braak, Thal, tau burden) to reinforce disease classification and interpretation.
2. Improve Legends for Cluster Names (e.g., mg.C4, ast.C1). Include descriptive names alongside cluster IDs in all figures (e.g., “myelin-related astrocyte”) to avoid forcing the reader to cross-reference multiple parts of the manuscript.
3.Clarify "Dynamic" Peak Definitions. Provide a simple schematic or glossary to distinguish between stable, dynamic, and DAR peaks, and explain how these were used in downstream analyses (e.g., GWAS enrichment vs. TF identification).
4. Consider Additional External Validation.
Test whether disease-specific CREs overlap epigenomic marks (e.g., H3K27ac, H3K4me1) from public datasets like Roadmap/ENCODE for relevant brain regions. Consider cross-checking TF target predictions (e.g., SOX10, MEF2C) using orthogonal datasets such as ChIP-seq.

(Remarks on code availability)
The code is well documented and although I did not download and run it myself I did not notice any issue in making that possible.

Reviewer #3

(Remarks to the Author)
Noteworthy Results
The results provide new insights into the distinct and shared epigenomic alterations in tauopathies, highlighting specific glial subtypes that may differentially contribute to disease pathology. The identification of disease-specific transcriptional regulators and the enrichment of genetic variants in certain glial subclusters are particularly noteworthy. Interestingly the authors describe a subcluster of microglia (mg.C4) being associated PiD with significant enrichment in myelin-related gene expression and increased cellular interaction, indicating its crucial role in PiD pathology. In addition, they highlight ast.C1 as a PSP-associated astrocyte subtype showing distinct chromatin accessibility with potential protective roles against tau pathology. This subcluster's unique features offers new insights into PSP-specific mechanisms and potential therapeutic targets.
Significance to the Field
This work is highly significant as it expands the understanding of the epigenomic landscape of tauopathies and identifies potential mechanistic pathways that differ across diseases. By uncovering how genetic variants may drive these changes, the study adds a new dimension to the established literature on the etiology and progression of neurodegenerative diseases.

Support for Conclusions and Claims
The authors generally support their conclusions with robust epigenomic and transcriptomic data. However, there are sections where the evidence could be better highlighted or made more comprehensible.
Flaws and Methodological Concerns. The methodology seems thorough and well-executed, leveraging state-of-the-art single-nucleus sequencing and integrating it with various genomic data.
1. Interpretation of -log10(FDR) values should be clarified, as some figures suggest non-significant results that could lead to misinterpretations.
2. Reproducibility: The methodological details are comprehensive, ensuring enough detail for reproducibility.
3. Figures and data presentation could be clearer and more consistent (e.g., ensuring appropriate coding for significance and clearer labeling).
1. General Comments:
• Correct the typo "FAMTOM5" to "FANTOM5".
• Clarify whether the context of "primarily tauopathies" indeed means "primary tauopathies".
2. Results and Figures:
• Comprehensive significance representation: Ensure p-values and FDR corrections are clearly marked, and figures indicate significant results visually.
• Supplementary Figure 4C needs clarification for better understanding.
• Rework Figure 3 and rename FTD as PiD or PiD/FTD to avoid confusion.
• Make modules in Figure 3I more distinguishing by using numbers as well as colors.
• The discussion of MPRA and QTL enrichments needs more organization and clarity.
• Align text descriptions of findings more closely with visual representations.
3. More specific questions:
1. Transcription Factor Variability:
• "TF variability in PSP glia deviates from other diseases, with glial types clustering naturally within PSP rather than grouping by cell types across diseases." It appears that AD also exhibits a similar but weaker clustering of transcription factor patterns in glial cells. Could this similarity be due to the fact that PSP is characterized by 4R tau and AD by a mix of 3R/4R tau, suggesting a gradient based on the tau forms related to the disease?
2. Functional Enrichment Significance:
• "Functional enrichment of cell type-specific CRE-linked genes revealed the general functions associated with the corresponding cell type identity (Fig. 2D)." The significance of certain findings in the figure, such as the enrichment of astrocyte-specific peaks in regulators of lamellipodia, is unclear. It appears that the color coding does not indicate significance clearly, making it difficult to interpret the findings for astrocytes and oligodendrocytes (ODC).
3. Down-Regulated DAR Patterns:
• "With PiD showing the apparent pattern of down-regulated DARs increasing in astrocytes, inhibitory neurons, and oligodendrocytes." This pattern is not unique to PiD but is also observed in PSP while being absent in AD. Is this pattern specific to pure tauopathies, as it seems common between PiD and PSP? If so, the functional effects of the genes linked to DA-CREs should be investigated in both diseases, not only in PiD.
4. Cluster-Specific Upregulation:
• It is challenging to determine if the upregulation observed in distinct clusters is specific to the subclusters listed by the authors (Figure 4J and G). While it is clear for HLA-DMB, it is not as evident for the other markers listed. Could the authors further elucidate these findings to clarify the upregulation patterns within these subclusters?

(Remarks on code availability)

Version 1:

Reviewer comments:

Reviewer #1

(Remarks to the Author)
I appreciate the authors having gone to great lengths to strengthen the paper. My initial point of the MPRA results being underexplored is not valid anymore - the integration of frVars with sn-eQTLs was a good idea. Revisions suggested by the other reviewers also add value to this paper, and the discussion is a lot easier to read now.

The authors correctly identified several studies having previously examined open chromatin architecture in AD and PSP, but they are neither cited, nor discussed - this should still be corrected.

(Remarks on code availability)

Reviewer #2

(Remarks to the Author)
The author clearly addressed all of my concerns and the manuscript in my opinion is suitable for publication.

(Remarks on code availability)
The code is well documented

Reviewer #3

(Remarks to the Author)
Authors answered all my questions appropriately. No further comments.

(Remarks on code availability)

Open Access This Peer Review File is licensed under a Creative Commons Attribution 4.0 International License, which permits use, sharing, adaptation, distribution and reproduction in any medium or format, as long as you give appropriate credit to the original author(s) and the source, provide a link to the Creative Commons license, and indicate if changes were made.
In cases where reviewers are anonymous, credit should be given to 'Anonymous Referee' and the source.
The images or other third party material in this Peer Review File are included in the article's Creative Commons license, unless indicated otherwise in a credit line to the material. If material is not included in the article's Creative Commons license and your intended use is not permitted by statutory regulation or exceeds the permitted use, you will need to obtain permission directly from the copyright holder.
To view a copy of this license, visit https://creativecommons.org/licenses/by/4.0/

SUMMARY OF REVISION

Rebuttal Summary

We thank the reviewers for their constructive and insightful feedback. In response, we have substantially revised and strengthened the manuscript to clarify and expand upon our central discovery: **disorder-specific dynamic gene regulation** that aligns with **heritability architecture** and engages **resilience pathways** under disease-reactive conditions. Our results provide **direct functional evidence** that common genetic variation converges on defined domains of **microglial reactivity**, shaping downstream signaling programs relevant to disease causality.

By integrating **high-resolution MPRA data**, we delineate convergence within **transcription factor (TF) networks**, identifying the ***MEF2C-MEF2D* network** as a critical intersection linking human genetic variation, microglial reactivity, and glial resilience. Given that ***MEF2C*** and ***MEF2D*** harbor disease-associated variants that influence *GUCY1A1*, *IL27RA*, and *TMEM106B*, these findings establish a **cohesive, mechanistic framework** for understanding how common genetic variation drives both shared and disorder-specific disease pathways. We have **streamlined the manuscript** for clarity and impact, and incorporated **new complementary analyses** that enhance the rigor, resolution, and interpretive depth of our conclusions, as detailed below.

New Analyses and Revisions

1. Introducing new data from **single-cell CUT&Tag histone code profiling** (H3K27ac and H3K4me1) across 22,860 cells from human cortex, including both AD and control samples, verifying the quality and annotation of both stable and dynamic cis-regulatory elements (CREs) mapped using snATAC-seq and snRNA-seq.
2. Adding additional genetic association validation through integration of MPRA and eQTL datasets from the neocortex of 424 individuals of advanced age[52], and the prefrontal cortex, temporal cortex, and white matter from 192 individuals, including controls, AD, and multiple sclerosis[53], confirming enrichment of genetic signals within microglial modules and distinct functional pathways.
3. **Expanded transcription factor (TF) network analysis**, identifying *MEF2C*- and *MEF2D*-bound enhancers as a major hotspot for human genetic variation and FTD GWAS signals
4. **Refined annotation of FTD GWAS convergence** at the level of TF networks active in disease-context dynamic microglial responses, co-localizing established genome-wide genes with additional, previously uncharacterized genes connected by convergent TFs and CREs, including rare variants with potential relevance to FTD pathology.
5. **MPRA construction verification using MPRA HMC3 RNA-seq and human consensus CRE annotation,** demonstrating enrichment of microglia regulatory variants on enhancers within the human vivo-like microglia model.

6. **Enhanced characterization of PiD-related microglia** in progression, differentiating the resilient state from pathological DAM by distinct gene programs.
7. **Comprehensive resolution of all technical comments**, ensuring full methodological transparency, reproducibility, and alignment with reviewer recommendations.
8. **Immunostaining-based validation** of rare, context-dependent microglial and astrocytic states identified in human brain tissue.
9. **Cell-based validation** of rare disease-associated states and context-specific microglial gene regulation, supporting the generalizability of our findings across systems.

Together, these additions significantly strengthen the manuscript, providing **multi-layered validation** of our findings and **robust functional grounding** for the proposed framework linking genetic variation, microglial reactivity, and disease-associated resilience mechanisms.

We present these as a point-by-point response to review below.

REVIEWER COMMENTS

Reviewer #1 (Remarks to the Author):

The authors profile chromatin accessibility (snATAC-seq) across Alzheimer's disease (AD), Pick's disease (PiD) and progressive supranuclear palsy (PSP), integrate the data with matched snRNA-seq, and survey cis-regulatory elements, transcription-factor activity, and GWAS enrichments. The MPRA is a particular strength of the study, although data-wise underexplored. While technically solid, methodologically done well and decorated with beautiful figures, virtually the entire dataset and many of the analytical pipelines were presented in their earlier study (Ref. 19). In this submission the authors largely revisit those data with incremental analyses; the narrative remains descriptive and no specific biological mechanism is functionally validated. Novelty is minimal, and there is redundancy with recent AD and PSP community resources describing altered microglial and astrocytic CRE environments. Results are reported as lists of subclusters, peaks, and GWAS enrichments, but there is no unifying hypothesis tested or mechanistic model proposed. The reader is left without a clear take-home message. The article may be better suited as a Data Resource with a short, focused extension on one of the many possible storyline threads (e.g. lysosomal pathway activation in PiD, FTLD microglia MPRA integration, or cell-cell interactions).

Reviewer 1 Point 1: "The MPRA is a particular strength of the study, although data-wise underexplored."

Response: We thank the reviewer for recognizing the strength of the MPRA component. In this study, the MPRA provides a mechanistic anchor that links human genetic variation to glial regulatory function. By measuring variant effects at nucleotide resolution, we identify which transcription factor (TF) binding sites are directly perturbed by naturally occurring alleles and

determine how these disruptions shift chromatin accessibility, TF network activity, and downstream microglial states. This framework reveals that regulatory variants do not act diffusely across the genome, but preferentially converge on specific, disease-responsive cis-regulatory programs in disease microglia, particularly those governing lysosomal function, myelin interaction, and stress-activated signaling. Thus, the MPRA establishes a principled framework demonstrating that functional variation tunes the activation thresholds of core microglial pathways, shaping individual susceptibility, the magnitude of glial reactivity, and the trajectory of microglial state transitions across health and disease.

To strengthen this point, we expanded our analysis by integrating MPRA-derived functional variants (frVar) with single-nucleus eQTLs from over 400 individuals. This integration revealed a striking level of convergence (Page 15, lines 459-468):

"Over 80% of the modules generated from frVar align with those independently derived from brain snQTL data in microglia (Supplementary Fig. 13). Such orthogonal agreement demonstrates that these MPRA-defined variants capture stable, biologically meaningful regulatory programs rather than assay-specific effects. Importantly, 12 of the 14 frVar modules correspond directly to an eQTL module, and 9 demonstrate consistent specification for the same microglial states. This tight alignment indicates that common genetic variation preferentially affects the same CRE networks that undergo dynamic remodeling in disease, reinforcing a mechanistic model in which genetic variants modulate microglial state transitions by targeting TF-centered regulatory hubs. Together, this convergence strengthens the interpretation that frVar captures the functional axis along which genetic risk influences microglial behavior.

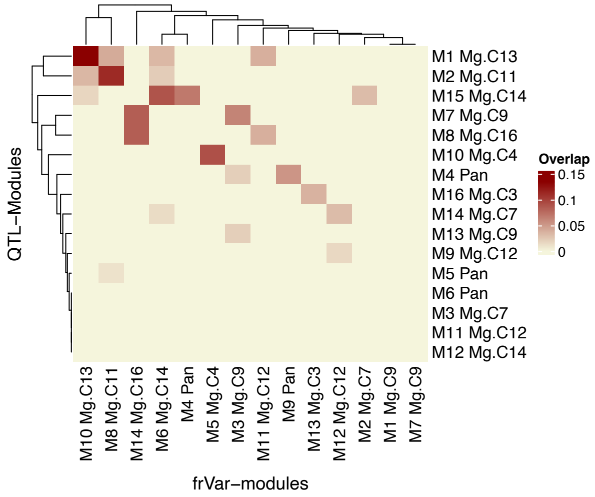

Supplementary Fig. 13. Convergence between MPRA-validated functional variant modules and microglial eQTL-derived CRE modules. Microglial sn-eQTL CRE modules (rows) were compared with frVar-derived modules (columns) using fractional CRE overlap, defined as the intersection divided by the smaller module size. Of the 14 frVar modules, 12 aligned with a corresponding eQTL module, and 9 of these module pairs exhibited consistent microglial state specificity."

To further dissect these convergent regulatory modules, we conducted topic modeling on CREs harboring either frVar or eQTLs in microglia. This analysis revealed that the regulatory framework identified in the overlap analysis resolves coherent and biologically meaningful programs associated with disease-related microglial states. Notably, Topic 9 specific to PiD-associated

mg.C4 captures regulatory programs involved in both stress-responsive and resilience-related processes, with well-defined target genes and transcription factors refined by MPRA frVars. Importantly, the *MEF2C*- and *MEF2D*-centered regulatory network, which was previously identified through independent analyses of MPRA frVars, is recapitulated in this topic-modeling framework, further supporting the robustness of this regulatory architecture and implicating *MEF2C* in mg.C4-associated regulatory program.

We have revised these components in the manuscript and added new figures (Page 16, lines 471-520):

"**Convergent gene regulatory modules regulated by MPRA frVar and eQTLs**

To deepen our understanding of functional variants converging within gene regulatory modules, we extended our analysis by applying topic modeling to CREs containing either frVar or sn-eQTLs in microglia. This method helped us determine whether the regulatory structure observed in the overlap analysis reflects coordinated biological programs or simply results from shared genomic location or CRE abundance. Topic modeling revealed nine microglial regulatory programs, each enriched for specific TF motifs and involved in disease-related microglial states (Supplementary Fig. 14). For instance, the PiD-associated mg.C4 program was enriched in pathways linked to stress activation and lysosomal-myelin interactions (Supplementary Fig. 14C-D) driven by *JUNB* and *SPI1*, along with resilience-related pathways involving phagocytic recognition, metabolic homeostasis, and myelin maintenance driven by *MEF2C* and *RHOXF2* (Supplementary Figs. 15A-B). This suggests that PiD-related mg.C4 contains functional variants capable of triggering stress-response pathways relevant to PiD pathology and supporting resilience. Importantly, the *MEF2C*-centered regulatory network, which was previously identified through independent analyses of MPRA frVars, is recapitulated in this topic-modeling framework (Supplementary Figs. 15D), further supporting the robustness of this regulatory architecture and implicating *MEF2C* in mg.C4-associated regulatory program. Overall, this analysis reinforces the mechanistic understanding that functional and expression-modifying variants converge within dynamic CRE networks, tuning TF-regulated pathways and directing microglial state changes specifically engaged in disease contexts.

To further evaluate whether these functional regulatory programs capture disease-relevant genetic signals that may be underpowered or missed in GWAS, we next examined p-value distributions of variants across GWAS and functional datasets. Approximately 13% of the functional variants within these topics fall below MAF 0.05. Detection of low-frequency variants under a genome-wide significance threshold ($\alpha = 5 \times 10^{-8}$) with >80% power, assuming a modest genotype relative risk (OR = 1.1), requires cohorts on the order of ≥100,000 total individuals (GAS power calculator, REF). In contrast, current FTD and PSP GWAS remain substantially smaller (N_FTD = 12,928, N_PSP = 8,363), limiting power to identify modest-effect regulatory associations. In the PiD mg.C4, we found 16 variants converging in topic 9 that exhibit functional regulatory activity yet fall below genome-wide significance thresholds ($p < 5\times10^{-8}$) in AD GWAS (Supplementary Figs. 15C). Notably, several variants lie near known AD risk genes such as *TREML4*, *RIN3*, *ALCAM*, and *CD300A*, as well as new candidates such as *SLC25A37*, *STK10*, and *MARK3*. *MARK3* encodes a kinase that phosphorylates tau and may affect tangle formation; *SLC25A37* is a mitochondrial iron transporter associated with iron dysregulation; and *STK10*, a kinase involved in T-cell signaling and inflammation, although less studied in the brain. Furthermore, *RABEP1*, an

endosomal trafficking protein crucial for lysosomal pathways, APP processing, and microglial phagocytosis, showed significance in both GWAS and functional assays, highlighting its role as a potential convergent disease node. These results indicate that functional regulatory variants, validated through MPRA and supported by sn-eQTL and chromatin accessibility, capture disease-relevant biology even when they fall below GWAS significance thresholds.

Together, these analyses demonstrate that frVar and eQTLs converge on biologically coherent and disease-relevant microglial programs, and that functional variants within these programs highlight regulatory mechanisms not fully captured by GWAS alone. This supports a model in which human functional genetic variants modulate microglial responsiveness by tuning TF-governed pathways that are selectively engaged across tauopathies."

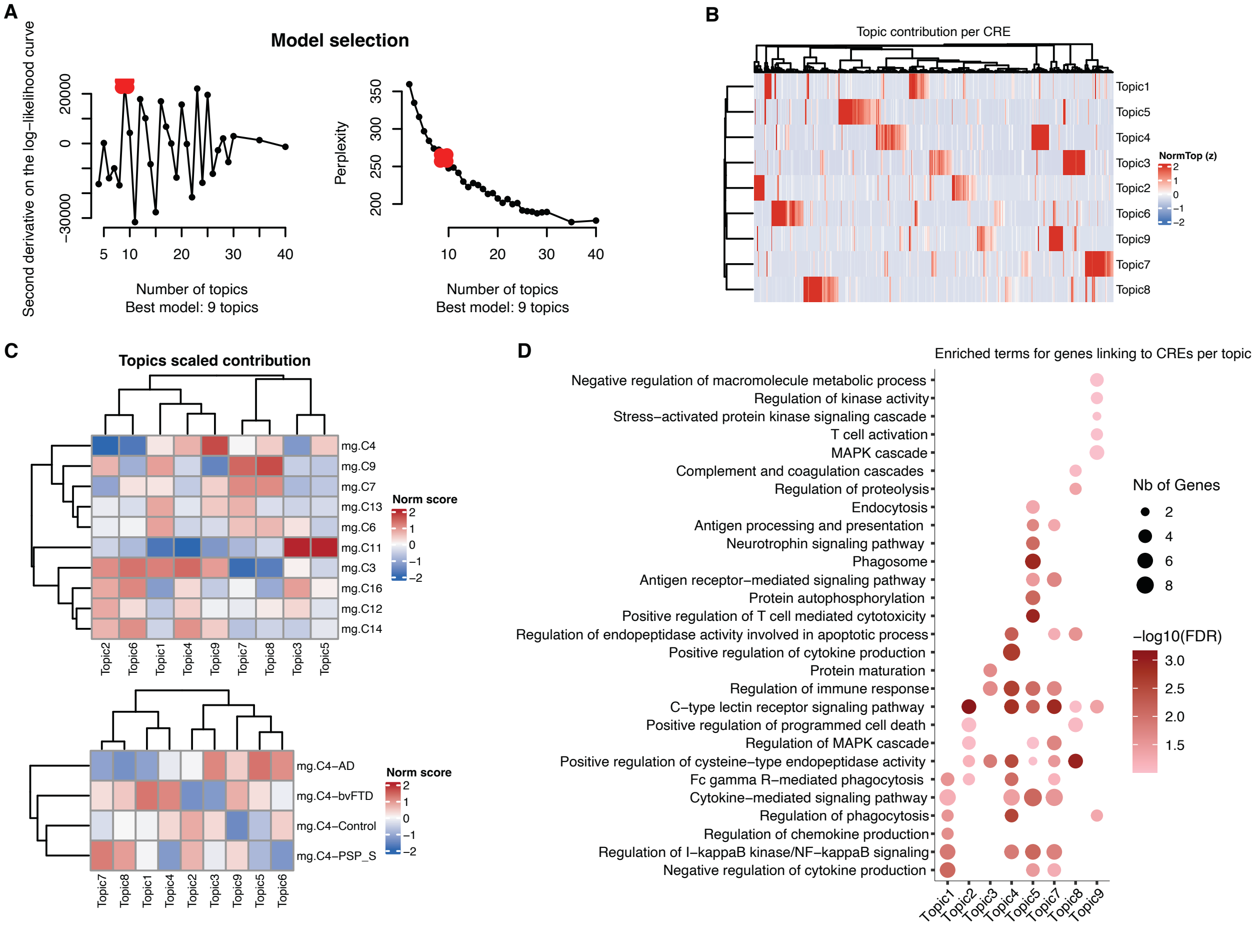

Supplementary Fig. 14. Regulatory programs for CREs containing frVar or eQTLs.
(A) Selection of topic numbers for microglial CREs containing frVar or eQTLs using the derivative method in cisTopic.
(B) Heatmap showing topic contributions across CREs.
(C) Comparison of relative topic contributions across microglial subtypes and in PiD-associated mg.C4.
(D) Top enriched TFs and associated functional pathways for each topic.

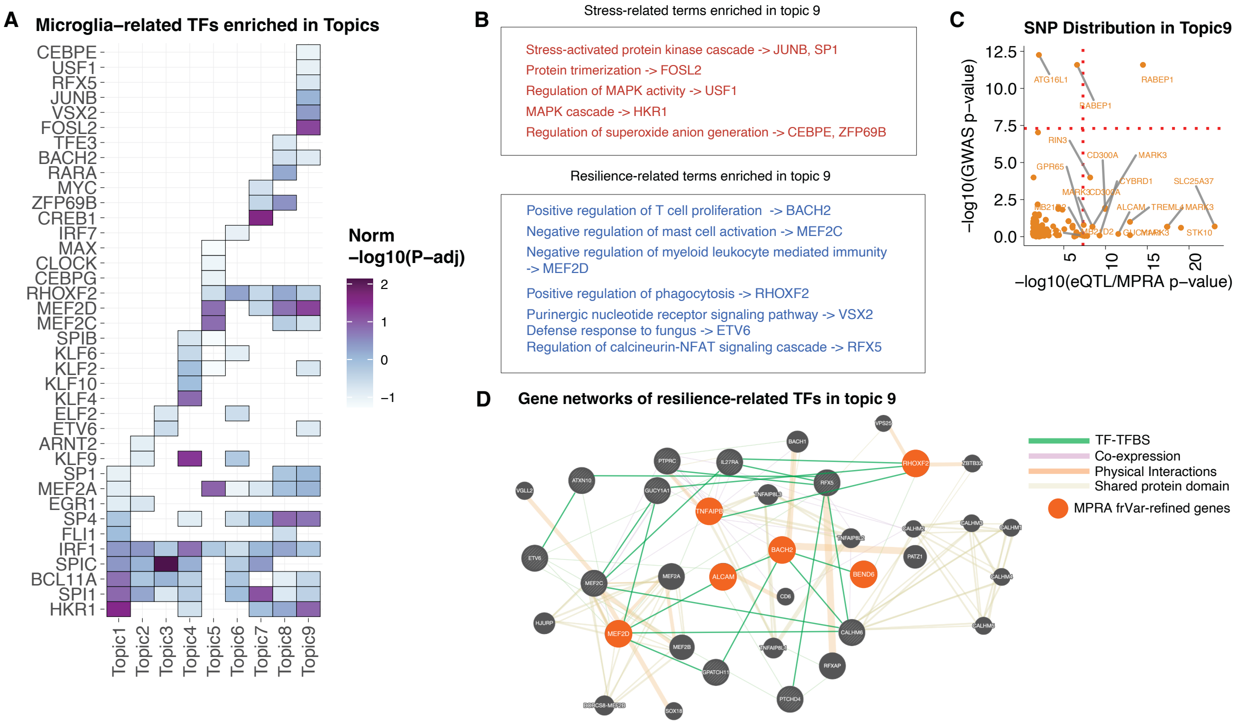

Supplementary Fig. 15. Regulatory programs associated with CREs containing frVar or eQTLs linked to disease.
(A-B) Enriched transcription factors (A) and associated functional pathways (B) across topics.
(C) Distribution of genetic variants within Topic 9, emphasizing significance in AD GWAS and functional assays.
(D) Gene network for Topic 9 generated using GeneMANIA, illustrating target genes regulated by resilience-related transcription factors. Genes refined using MPRA-validated functional variants (frVars) are highlighted as orange nodes."

Reviewer 1 Point 2. "While technically solid, methodologically done well and decorated with beautiful figures, virtually the entire dataset and many of the analytical pipelines were presented in their earlier study (Ref. 19)."

Response: We appreciate the reviewer recognizing the technical rigor and methodological quality of our work. However, we respectfully disagree with the suggestion that the analytical pipelines were already introduced in our previous study (Rexach *et al., Cell*, 2024). In the previous study, snATAC-seq was used primarily to validate modules derived from snRNA-seq. Here, snATAC-seq is the central modality and serves a fundamentally different purpose: to define disease-associated chromatin dynamics, identify cis-regulatory programs not detectable at the RNA level, and map how these programs intersect with human genetic variation across multiple primary tauopathies.

Our rationale for an ATAC-centered framework is twofold. **First**, our prior snRNA-seq analyses showed that disease modifiers influence microglial and astrocytic states in more distinct ways than they influence shared programs, indicating that the most disease-relevant regulatory differences arise where cell states diverge. Because genetic modifiers exert their effects through gene

regulation, this observation suggested that chromatin accessibility, rather than RNA abundance, would provide a more proximal and mechanistically informative view of how disease-context cell states are specified. Consistent with this, snATAC-seq resolved subclusters such as mg.C4 and mg.C6 that appeared merged at the transcriptomic level yet displayed markedly different enrichments for FTD GWAS associations. These distinctions and their genetic relevance would not have been detectable with RNA alone, underscoring the unique insights that accessibility-based clustering can provide.

Second, the present study interrogates dynamic regulatory programs, CRE modules that are remodeled in disease, to determine whether genetic risk preferentially impacts the TF-driven circuits that change under pathological conditions rather than those stable at baseline. This analysis, which was not possible in Rexach *et al., Cell*, 2024, reveals that disease-responsive chromatin programs are selectively enriched for functional MPRA variants and sn-eQTLs, thereby defining **mechanistic axes of susceptibility and resilience** that are not identifiable from transcriptomic states alone. Together, these advances make the current work conceptually and methodologically distinct from our earlier study, establishing a **chromatin-centered framework** for how human genetic variation interacts with disease-context regulatory remodeling to shape microglial state transitions in neurodegeneration.

Reviewer 1 Point 3. "In this submission the authors largely revisit those data with incremental analyses; the narrative remains descriptive and no specific biological mechanism is functionally validated… Results are reported as lists of subclusters, peaks, and GWAS enrichments, but there is no unifying hypothesis tested or mechanistic model proposed. The reader is left without a clear take-home message.""

Response: We respectfully disagree with the notion that our work is only an incremental revisit of previous data. In this study, we thoroughly reexamined snATAC-seq data from a different angle than our earlier work, which focused on snRNA-seq. Our goal was to uncover the genetic, epigenetic, and transcriptional feedback loops driving cellular changes during disease progression. To achieve this, we conducted a new functional MPRA assay, combined these findings with sn-eQTLs and GWAS data, and carefully analyzed cis-regulatory elements and gene programs at both the cell type and disease levels. Far from being descriptive, the revised manuscript now presents a unified model in which disease-context chromatin remodeling selectively engages TF-driven regulatory programs that influence vulnerability and resilience across primary tauopathies. This framework is supported by three independent lines of evidence: (1) identification of previously unrecognized, disease-specific glial states; (2) functional validation through in situ and in vitro assays; and (3) regulatory dissection of CRE-TF-gene circuits altered across disorders.

First, our chromatin-focused approach uncovered two new glial states linked to tauopathies: PSP-specific ast.C1 and PiD-specific mg.C4. Both states are enriched in pathways related to myelin interaction, lysosomal activity, and metabolic stress responses. These states are not detectable through transcriptomic analyses alone and signify separate pathways of microglial and astrocyte adaptation. Their regulatory profiles indicate that disease-related changes in chromatin may trigger myelin-lysosomal activities that help manage metabolic or proteostatic stress, potentially acting as resilience mechanisms.

Second, to validate these interpretations, we performed immunohistochemistry (IHC) and RNA scope on human insula sections and confirmed an increased prevalence of myelin-related astrocytes and microglia in PSP and PiD compared to controls, supporting the presence of the myelin-interacting glial populations in vivo. In parallel, we conducted CRISPR activation of *SOX10* in iPSC-derived microglia, followed by RNA-seq, to validate lysosomal reactivity and a microglial beneficial response after *SOX10* induction. This experiment showed coordinated upregulation of lysosomal, phagocytic, and myelin-processing pathways, consistent with the programs enriched in mg.C4. Together, these functional assays corroborate the biological roles inferred from chromatin accessibility and demonstrate that these regulatory programs can be induced by disease-relevant stimuli.

Third, to examine how these glial states emerge in disease, we performed Slingshot trajectory analysis using gene activity data from snATAC-seq. This analysis revealed that PiD-associated mg.C4 diverges from the disease-associated microglia (DAM) trajectory and instead progresses toward a lineage characterized by increasing activation of genes governing autophagy-lysosome function (*PARK7*, *TFEB*, *NFE2L1*), lipid homeostasis (*ADAM10*), and myelination (*CNP*, *OLIG2*, *MOG*). In contrast, the DAM lineage upregulates inflammatory regulators such as *NRROS* and *PRDM1*. These findings indicate that mg.C4 represents a distinct, non-inflammatory trajectory, suggesting that microglia may adopt myelin-lysosome programs as an adaptive response to tau pathology rather than progressing exclusively toward DAM.

Finally, we strengthened the mechanistic interpretation by highlighting cell state-specific CRE-TF-gene circuits uncovered by snATAC-seq. For example, a PiD-specific enhancer of *GRN* in mg.C14 contains *FOS* and *CEBPB* motifs, TFs known to activate stress and immune response pathways, indicating a regulatory mechanism by which PiD microglia increase *GRN* expression. In contrast, in PSP, a *TREM2* enhancer accessible only in *TREM2*$^{+}$ mg.C13 shows reduced activity and contains motifs for *BCL11A* and the microglial TF *SPI1*. The decreased accessibility of this enhancer offers a plausible explanation for lower *TREM2* expression and compromised microglial resilience in PSP.

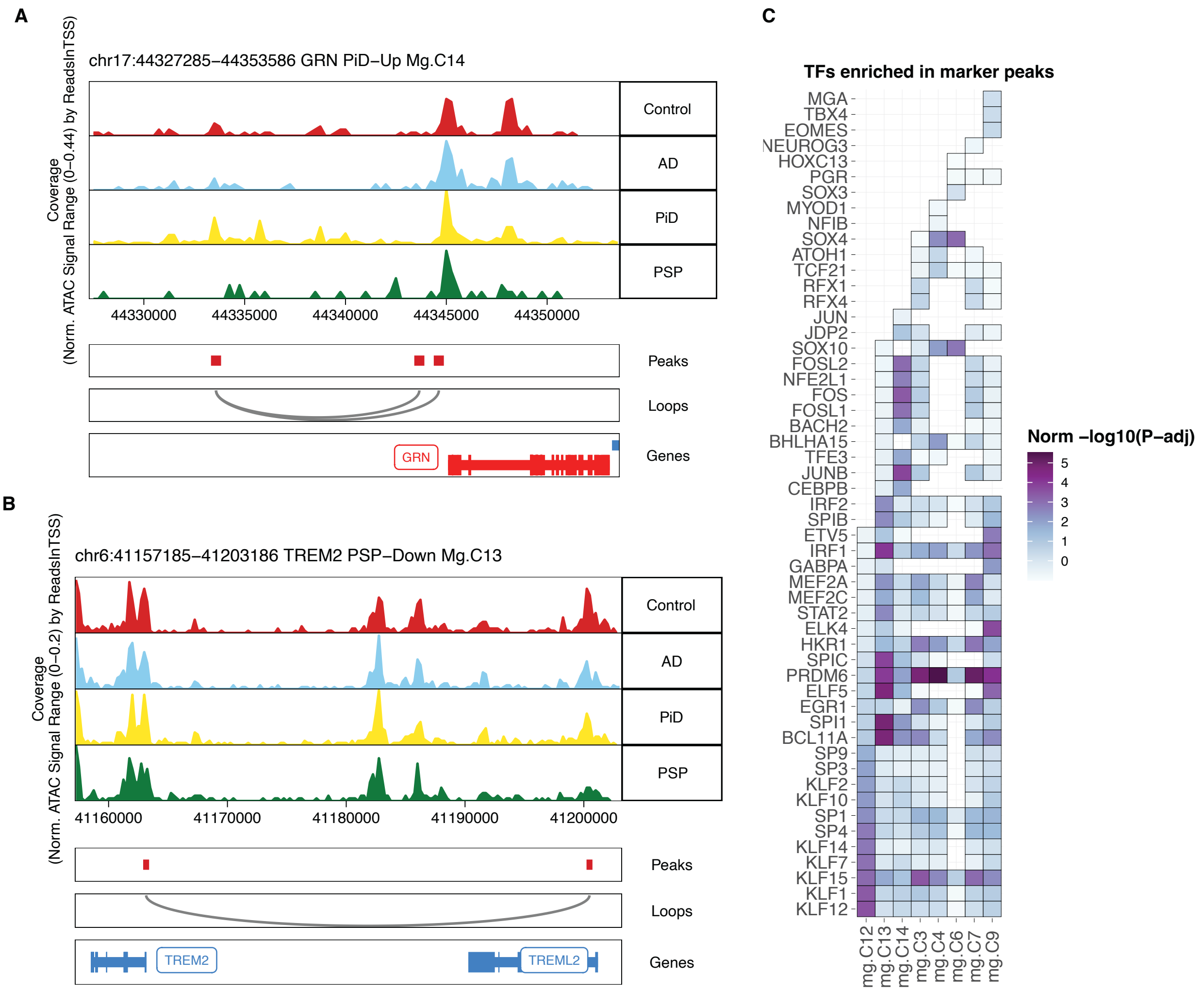

Supplementary Fig. 21. Disease-specific enhancer accessibility and TF motif enrichment in microglial subtypes.

(A) Genome browser tracks illustrating differential enhancer accessibility across disorders, showing PiD-specific activation of the *GRN* enhancer in mg.C14.

(B) Genome browser tracks showing reduced accessibility of the *TREM2* enhancer in PSP within mg.C13.

(C) Motif enrichment analysis of subtype-defining peaks performed using MEME. Normalized false discovery rate (FDR) values are shown for enriched transcription factors (TFs) in each microglial subtype.

Together, these results demonstrate that our study does not simply revisit prior data but instead defines a coherent mechanistic model**: disease-context chromatin dynamics selectively activate TF-driven regulatory programs, which integrate genetic risk and pathological cues to produce distinct microglial and astrocytic response trajectories.** These trajectories, validated by IHC, stimulation assays, and trajectory analysis, define biologically coherent mechanisms of susceptibility and resilience and establish the unified regulatory framework linking chromatin remodeling to disease progression across tauopathies.

We incorporated these new components into the manuscript on Page 19, lines 583-596:

"To illustrate how disease-context chromatin remodeling engages specific glial populations, we identified cell type-specific CREs that were differentially activated in disease contexts. For instance, in mg.C14, we found a CRE of GRN that was uniquely open in PiD (Supplementary Fig. 21A). This CRE included binding motifs for *FOS* and *CEBPB*, key drivers of mg.C14. *FOS* motifs, known to be enriched in stress and immune responses, especially under Aβ stimulation, while *CEBPD* is a significant regulator of inflammatory signaling. Activation of this enhancer likely reflects a stress-induced regulatory circuit through which PiD microglia elevate *GRN* expression. Conversely, in PSP we identified a *TREM2* enhancer selectively downregulated in *TREM2*$^{+}$ mg.C13 (Supplementary Fig. 21B). This CRE contained motifs for *SPI1* and *BCL11A*, both enriched in mg.C13. *SPI1*, an established AD risk factor, controls microglial identity and survival, whereas *BCL11A* acts as a chromatin regulator. The downregulation of this *TREM2* CRE in PSP suggests a regulatory mechanism where decreased *SPI1*/*BCL11A* activity may reduce *TREM2* expression, weakening microglial resilience in PSP. These findings illustrate how distinct disorders engage disease-specific CRE-TF-gene circuits in specific glial populations."

Updated in the manuscript page 22 lines 657-668:

"To investigate the protective role of mg.C4 and its divergence from the disease-associated pathway, we employed Slingshot[64] to infer pseudotime trajectories for microglia in the insula (Methods). This analysis included cells associated with PiD-linked mg.C4, DAM-linked mg.C12, and homeostatic mg.C16. Beginning from homeostatic mg.C16, we identified three main lineages: one progressing toward mg.C4, one toward mg.C12, and the third representing a mixture of all three subtypes (Supplementary Fig. 26). The mg.C4-directed trajectory showed gradual activation of autophagy-lysosome regulators (*PARK7*, *TFEB*, *NFE2L1*), lipid homeostasis (*ADAM10*), and myelination (*CNP*, *OLIG2*, *MOG*). In contrast, the DAM-directed lineage upregulated inflammatory mediators including *NRROS*, *PRDM1*, and classical DAM markers such as *GPNMB* (Supplementary Fig. 27). Together, these trajectories show that mg.C4 is a distinct, non-inflammatory microglial state that engages coordinated myelin-lysosome programs as an adaptive response to metabolic and disease stress."

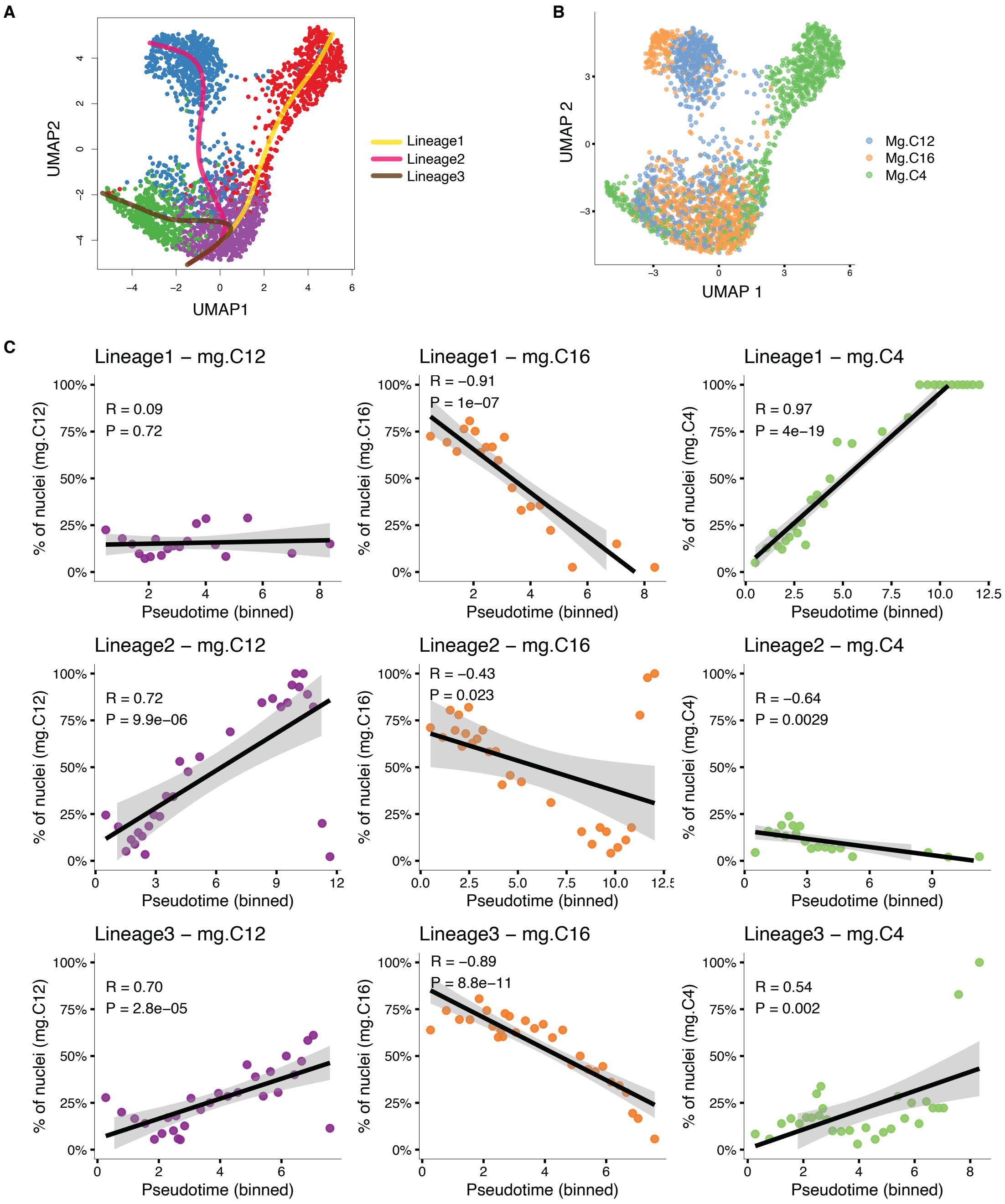

Supplementary Fig. 26. Slingshot pseudotime trajectories of microglial subtypes in the insula.
(A) UMAP embedding showing Slingshot-inferred pseudotime trajectories for homeostatic mg.C16, PiD-associated mg.C4, and DAM-associated mg.C12, colored by lineage assignment.
(B) The same UMAP colored by microglial subtype identity.
(C) Dot plot showing Pearson correlations between pseudotime values and microglial subtype abundance across the three inferred lineages.

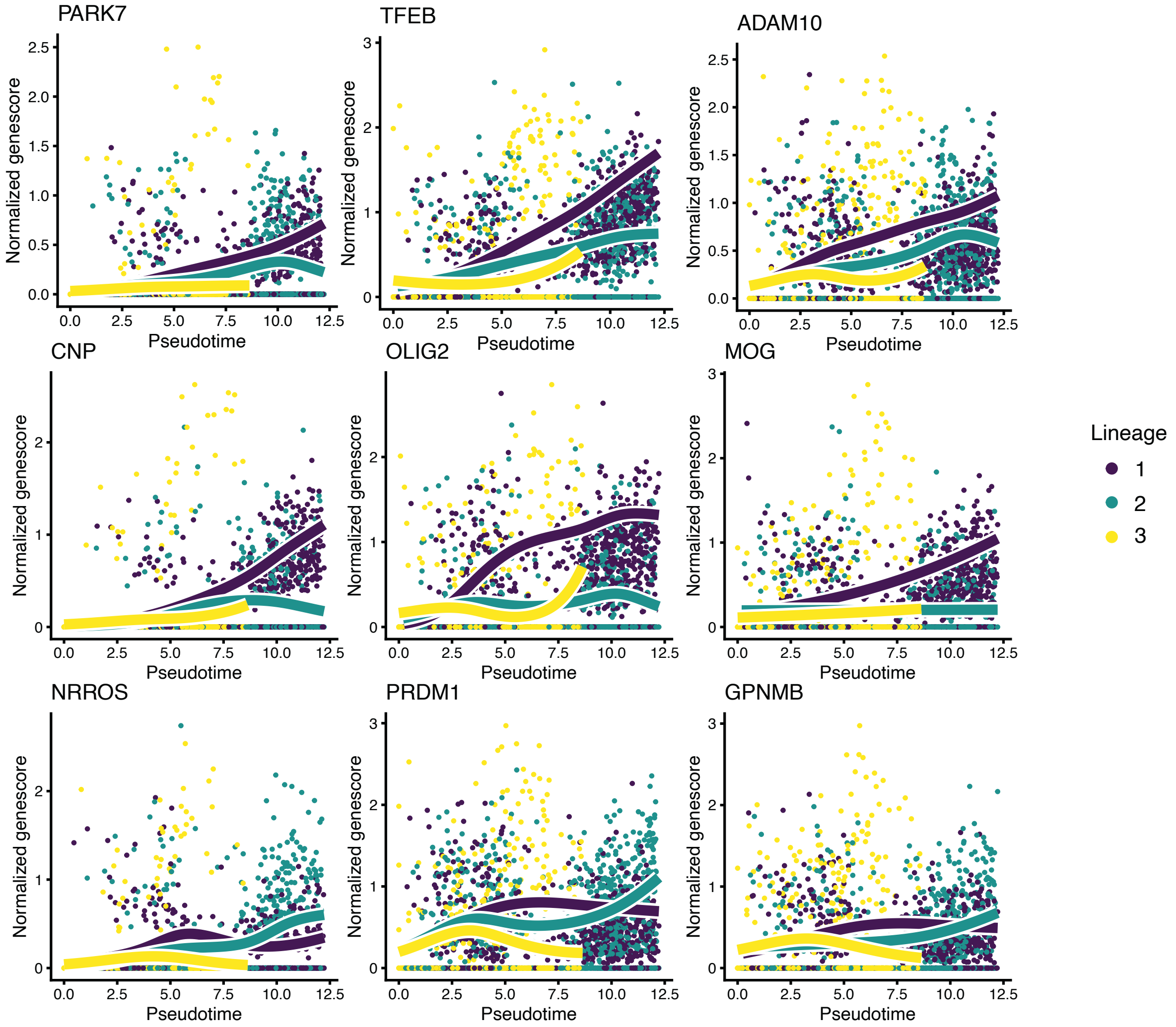

Supplementary Fig. 27. Gene activity along pseudotime trajectories of microglial subtypes in the insula. Slingshot trajectories were inferred for homeostatic mg.C16, PiD-associated mg.C4, and DAM-associated mg.C12. Points represent individual cells, colored by pseudotime lineage assignment. Normalized gene activity scores across along pseudotime were modeled using generalized additive models (GAM; y ~ s(pseudotime)) for each gene-lineage combination.

Reviewer 1 Point 4. "Novelty is minimal, and there is redundancy with recent AD and PSP community resources describing altered microglial and astrocytic CRE environments."

Response: We respectfully disagree with the assessment that our study's novelty is minimal or repetitive compared to existing AD and PSP resources. Although several recent studies have explored chromatin or transcriptomic changes in PSP and AD, our work differs in scope, methodology, and biological insights.

Prior PSP studies were limited by small sample sizes, restricted brain regions, or a lack of regulatory resolution. Whitney *et al.*, *Acta Neuropathol,* 2024 conducted snRNA-seq on the

diencephalon of six PSP samples and identified EIF2 signaling in vulnerable cell types, though could not resolve cis-regulatory mechanisms because chromatin accessibility was not profiled. Briel *et al., Acta Neuropathol,* 2022 performed snATAC-seq from the frontal cortex in PSP and CBD but with only four samples per group, observing widespread astrocytic chromatin changes associated with TFEB and NFE2 while limiting power to dissect glial subtype heterogeneity or define cell state-specific regulatory circuits. Updated PSP GWAS (Farrell *et al., Nat Commun*, 2024) has identified genetic risk factors but does not map how these variants act within specific cellular populations. Thus, existing works and resources do not establish how disease-context chromatin remodeling engages genetic risk at the level of distinct glial states across tau disorders. A recent publication comparing the prefrontal cortex of PiD compared to AD datasets (Shi *et al., Science Advances*, 2025) contrasted PiD and AD disorders at the analysis stage rather than with combined randomized libraries prepared together across diagnoses. The results supported cellular and molecular differences in PiD vs AD as a phenomenon, and further showed that brain regional differences limit comparisons of PiD with our published snRNA-seq datasets of distinct regions, as they were compared and differed, undercoring the distinct value of these works. The specific findings presented in our work, including context-associated dynamic chromatin and disorder-specific glial states, were not similarly explored in that study.

In AD, larger-scale multi-regional studies combining transcriptomic and epigenomic data have provided valuable insights. Several high-quality studies reveal that specific microglial and astrocytic subtypes display epigenomic changes with TF motif alterations linked to processes like lipid metabolism, inflammation, synapse support, calcium signaling, and cell identity loss. These changes are observed across multiple vulnerable brain regions, including the entorhinal cortex, prefrontal cortex, and hippocampus (Sun *et al., Cell*, 2023; Morabito *et al., Nat Genet*, 2021, Kosoy *et al., Nat Genet*, 2022; Xiong *et al., Cell*, 2023; Anderson *et al., Cell Genom*, 2023; Nott *et al., Science,* 2019; Liu *et al., Cell*, 2025). However, these findings cannot be generalized to PSP or PiD, as tauopathies differ markedly in regional vulnerability, cell state engagement, and underlying regulatory mechanisms.

Our work addresses these limitations by systematically integrating snATAC-seq, snRNA-seq, sn-eQTLs, MPRA, and sn-Cut&Tag and GWAS across multiple brain regions that exhibit varying patterns of vulnerability in AD, PiD, and PSP. These areas show less pathology in AD but are significantly impacted in PiD and PSP, making AD a useful comparison and highlighting tauopathy-specific biology. This cross-disorder framework allows us to distinguish conserved glial programs from disorder-specific regulatory remodeling, overcoming normalization and batch issues that arise when datasets are analyzed separately.

Unlike previous studies focused solely on AD, our research systematically combines snATAC-seq with snRNA-seq, eQTLs, MPRA, and GWAS across three brain regions that vary in vulnerability. By examining multiple disorders within a single framework, we identify both shared regulatory mechanisms and disease-specific changes that are hard to detect across separate datasets due to normalization and study design issues. Using this approach, we identify two previously unrecognized myelin-lysosome glial states, PiD-associated mg.C4 and PSP-associated ast.C1, that are not present in AD datasets. These states highlight distinct adaptive regulatory programs that would not emerge from AD-focused resources alone.

Reviewer #1 (Remarks on code availability):
The code is organized well, documented sufficiently and easy to read - this is a strength of the paper. Upon random interrogation of the R script files, I could not find any mistakes.

Response: Thank you for your acknowledgment. I value your thorough review and feedback.

Reviewer #2 (Remarks to the Author):

Reviewer 2 Summary Comment:

This study employs integrated single-nucleus ATAC-seq and RNA-seq analyses of brain tissue from Alzheimer's disease (AD), Pick's disease (PiD), progressive supranuclear palsy (PSP), and controls across three brain regions. The authors focus on disease- and cell-type-specific chromatin accessibility dynamics, identify cis-regulatory elements (CREs), and analyze their functional relevance through integration with GWAS summary statistics, eQTLs, and massively parallel reporter assays (MPRAs). They highlight disorder-specific glial subtypes, particularly ast.C1 and mg.C4, as states associated with chromatin accessibility changes linked to disease heritability and potential resilience mechanisms. The manuscript reads well, however I would like to raise a few major and minor concerns before it is published.

Summary Response: We appreciate the reviewer's thorough assessment of our manuscript and their acknowledgment of our work's contributions. We have thoughtfully addressed all major and minor concerns, making revisions to the text, figures, and supplementary materials as needed. Below, we provide detailed responses to each point.

Major Points
Reviewer 2 Point 1. Use of Outdated PSP GWAS Summary Statistics. The authors rely on a 2011 PSP GWAS (Höglinger et al.), despite the existence of more recent studies with larger cohorts and better-powered variant discovery (e.g., Wang et al., Mol Neurodegener 2024, Farrell et al 2024 Nature communications). This is particularly important since newer studies report variants not captured in earlier datasets, potentially altering interpretations of heritability enrichment and regulatory targeting in PSP. Analyses should be updated with recent summary statistics or at minimum benchmarked against them.

Response: Thank you for this important suggestion. We agree that updated PSP GWAS data provide a more complete variant catalog, and we have incorporated the recent GWAS (Farrell *et al., Nat Commun*, 2024) into our analysis. Using these summary statistics, we repeated LDSC on subtype-defining astrocyte peaks ($log_2FC > 0.5$, $FDR < 0.1$) and consistently observed significant PSP heritability enrichment in the ast.C1 subtype (Supplementary Fig. 25F). This enrichment was robust across both the original and updated PSP GWASs, confirming that ast.C1 captures the major PSP-associated regulatory signal.

Analysis with the updated GWAS additionally identified ast.C3, an antigen-presenting astrocyte state, as another top contributor to PSP heritability. However, ast.C3 does not exhibit significant differential chromatin accessibility in PSP tissue, indicating that although genetic risk maps to this subtype, it does not engage a PSP-disease dynamic or enriched epigenomic program of disease reactivity. Incorporating the expanded variant set further highlighted PSP-specific SNP-CRE circuits selectively upregulated in ast.C1, including regulatory elements linked to SNARE interactions, endocytosis, and cell migration (Fig. 5C; Supplementary Tables 1-2). These CRE were further enriched for protein metabolic pathways, suggesting metabolic stress responses analogous to those observed in PiD-associated mg.C4.

Together, these updates demonstrate that the newer GWAS data reinforce the conclusions of our study. A major PSP-associated regulatory program remains centered on ast.C1, reproduced across GWAS studies, with an expanded variant catalog revealing additional, disease-relevant non-coding circuits that further reinforce our mechanistic interpretation.

This component has been updated in the manuscript (Page 26, lines 779-789):

"To validate our results of PSP-related ast.C1 with a more recent PSP GWAS[69], we reran LDSC analysis on astrocyte subtype marker peaks (log2fc > 0.5 and FDR < 0.1). We observed PSP heritability was only consistently enriched in ast.C1, confirming this subtype specifically captures genetic signals linked to PSP (Supplementary Fig. 25F). Although LDSC with the updated GWAS also detected ast.C3, an antigen-presenting astrocyte state, this subtype did not show significant differential accessibility in PSP across any region, suggesting PSP does not trigger an epigenomic response in these cells. Using the expanded variant set from the updated PSP GWAS, we reliably identified SNP-CRE circuits upregulated in PSP ast.C1 involved in SNARE interactions, endocytosis, and cell migration (Fig.5C, in Supplementary Tables 1-2). These non-coding regulatory circuits were additionally enriched for protein metabolic activation (Fig.5C), indicating metabolic stress responses similar to those seen in PiD-associated mg.C4.

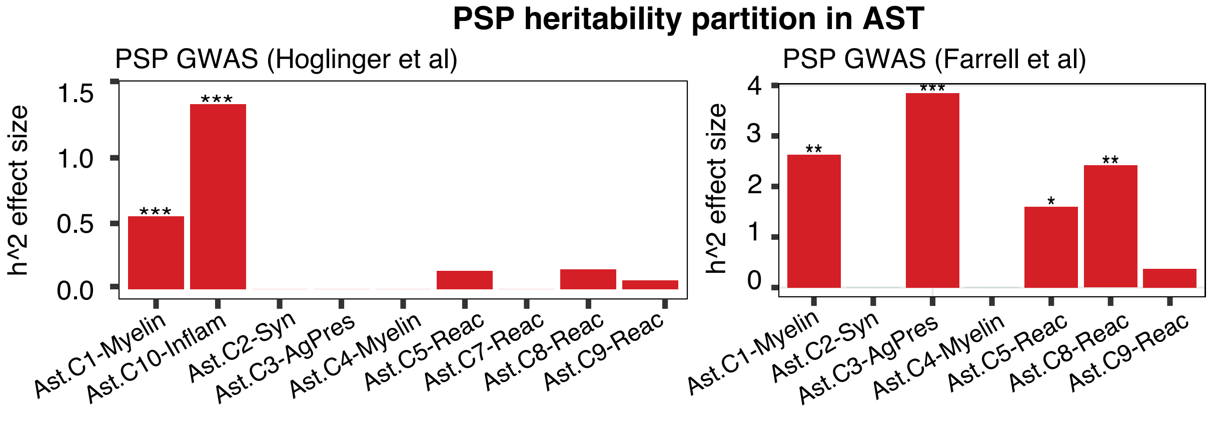

Supplementary Fig. 25. Disease heritability partition by LDSC in subcluster-specific peaks.

(F) GWAS heritability partition of PSP in neuron and astrocyte subcluster-specific peaks, using both LDSC standardized effect size and enrichment metrics. (significant for τ_c^*, FDR *< 0.05; ** <0.005; *** < 0.001)."

Reviewer 2 Point 2. Justification for Excluding Calcarine Cortex. The exclusion of calcarine cortex samples is stated to be due to "limited and variable" data, but this lacks sufficient justification. A more quantitative rationale is needed—e.g., cell yield, TSS enrichment, or clustering inconsistencies—and ideally visualized in supplementary figures to demonstrate that this region was unusable or inconsistent across samples.

Response: We excluded the calcarine cortex because data for this region were limited and inconsistent, preventing reliable cell-type or differential analyses. Only two samples per condition were collected, and although both cell yield and TSS enrichment passed quality control, the cell-type composition varied greatly between replicates. For example, the oligodendrocyte fraction ranged from 9.14% to 68.82% across matched calcarine samples-much more than in any other region. Due to this instability and with an effective sample size of n=2, robust modeling of cell-type shifts or chromatin changes related to disease was not possible. As shown in the Supplementary Fig. 22 (calcarine samples highlighted in yellow), this variability was specific to the calcarine cortex and not seen in PreCG or insula. Therefore, to maintain analytical rigor, we excluded the calcarine cortex from further analyses.

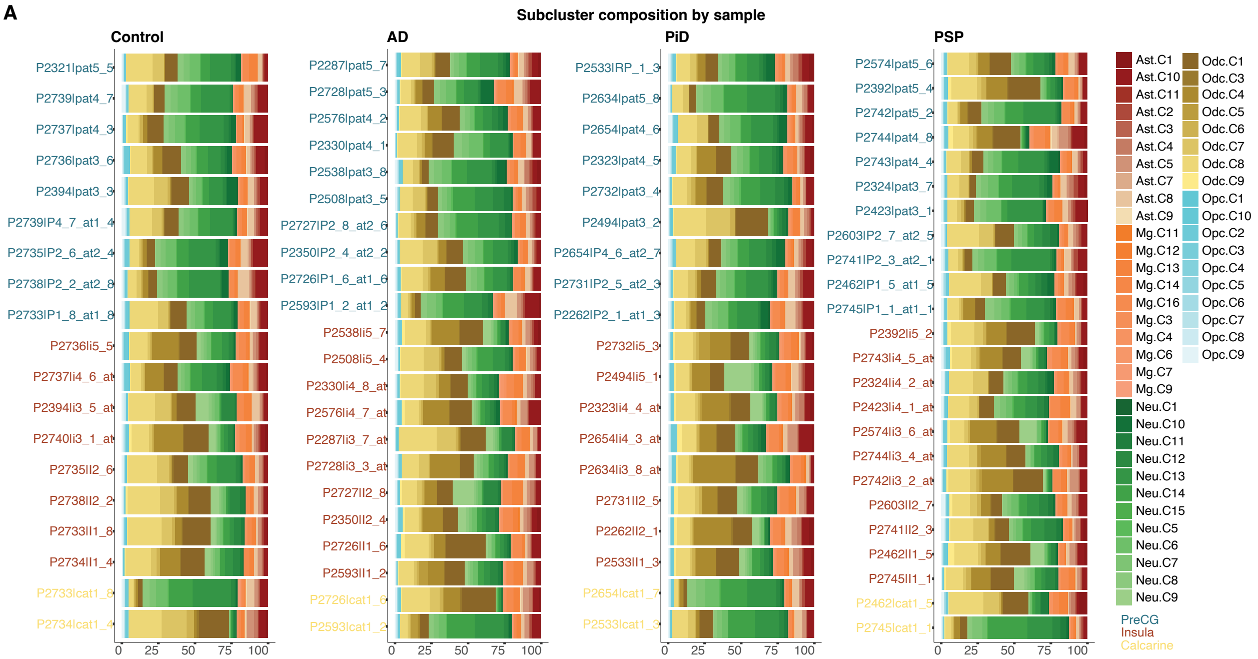

Supplementary Fig. 22. Cell distribution across subclusters in samples and changes in cell type composition. (A) Distribution of subclusters across samples, split by disease, with sample IDs colored by brain regions

We have added the following justification to the Methods section:

"Although calcarine samples passed basic QC metrics such as cell yield and TSS enrichment, clustering analysis revealed substantial inconsistencies in cell-type composition (Supplementary Fig. 22A). Across the two calcarine samples per condition, the oligodendrocyte fraction ranged from 9.14% to 68.82%, a level of variability incompatible with reliable downstream modeling. Given this instability and the limited sample size (n=2), calcarine cortex was excluded from all subsequent analyses."

Reviewer 2 Point 3. No Reported Quantification of Pathology in Analyzed Hemispheres. The manuscript lacks explicit data on pathology burden in the sampled hemispheres. While the contralateral hemisphere may be used for fixed tissue pathology, this should be clearly described. Quantitative pathology scoring (e.g., tau, amyloid, TDP-43, alpha-synuclein) for each brain region and sample should be provided, ideally in a supplementary table or figure, to confirm diagnostic classification and rule out comorbid pathology.

Response: Thank you. We have included the pathology burden details, including the quantified pathology scores and semi-quantitative scoring of neurodegeneration and tau pathology features for the samples, in the manuscript (Page 35, lines 1043-1056):

"Patients autopsied in this study underwent semi-quantitative scoring of neurodegeneration and tau pathology features as previously described[19]. The pathology data for each sample can be found in (Supplementary Table 1). The insula, PreCG, and calcarine cortex exhibit differential vulnerability across diseases, with the insular cortex showing a higher disease burden in PiD and PSP compared with AD (Supplementary Table 1). Neurodegeneration was scored based on hematoxylin and eosin stain and included microvacuolation, astrogliosis, and neuronal loss, each graded on a scale of 0 to 3 (absent, mild, moderate, severe). Tau aggregates were visualized using a monoclonal anti-phospho-tau (pS202) antibody CP13. Pathomorphologies were graded using the same 0-3 scale and included neurofibrillary tangles, Pick's bodies, neuronal cytoplasmic inclusions, globose tangles, astrocytic plaques, tuft-shaped astrocytes, thorn-shaped astrocytes, tau-positive threads and grains in both gray and white matter, and glial cytoplasmic inclusions. In addition to these scores, we have now added standardized neuropathological data from our samples to this manuscript (Supplementary Table 1)."

Reviewer 2 Point 4.No Discussion or Adjustment for Clinical Heterogeneity. The study does not mention antemortem clinical features, disease duration, symptom severity, or cognitive assessments, which are highly relevant for stratifying molecular data. Including these clinical parameters and adjusting for them where possible (or explaining why they were not considered) would strengthen the interpretation of disease-state-specific findings.

Response: Thank you for raising this point. Our study was intentionally designed to compare chromatin and regulatory programs **across distinct primary tauopathies**, each of which is defined by specific genetic, pathological, and regional vulnerabilities. As such, the goal of this work was **not** to model subtle clinical variation *within* a diagnosis, which would require substantially larger

cohorts and uniform antemortem assessment, but to identify disease-specific regulatory programs that distinguish PiD, PSP, and AD. Because these disorders differ markedly in etiology and pathology, cross-diagnosis contrasts provide a strong and biologically grounded axis of variation for interrogating chromatin dynamics and genetic convergence.

For this reason, we selected cases to **minimize intradiagnostic heterogeneity**: all samples had comparable, late-stage disease, were screened to reduce copathology, and exhibited **relatively tight distributions of semi-quantitative neuropathology scores** (Supplementary Table 2). These composite scores, which reflect neurodegeneration and tau burden, were available for most samples and demonstrate that cases within each diagnostic group were deliberately matched to reduce variability. This design improves the interpretability of cross-disease comparisons and avoids the confounding that would arise if within-diagnosis severity varied widely.

Although detailed antemortem clinical metrics such as symptom severity, cognitive assessments, or disease duration were not consistently available, this does not limit our central conclusions. These clinical variables typically modulate **within-diagnosis** variation, whereas our study defines **diagnosis-specific chromatin programs** that consistently appear across individuals within each disease. Indeed, even with limited clinical metadata, we observe an interpretable relationship: the PSP-associated astrocyte state ast.C1 shows a trend towards a negative correlation with composite pathology scores, suggesting that this myelin-interacting astrocytic program might have a protective or resilience-related function in PSP.

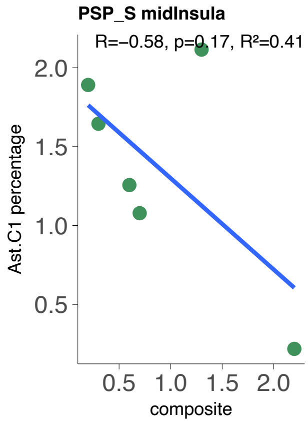

Overall, the absence of granular clinical stratification reflects the scope and design of the study rather than an analytic limitation. Our cross-disease framework, combined with pathology-matched case selection and minimized copathology, is well-suited for identifying **robust disease-specific regulatory programs** across major tauopathies.

Reviewer 2 Point 5.Lack of Validation for Identified Glial Subpopulations. While ast.C1 and mg.C4 are emphasized as disease-associated states, external validation is minimal. A more robust demonstration—such as immunostaining for PLP1 or SOX10 in multiple samples, co-labeling with disease markers, or external dataset replication—would strengthen the claim that these are conserved and disease-relevant glial subtypes.

Response: We appreciate the reviewer's suggestion to strengthen external validation for the disease-associated glial subtypes ast.C1 and mg.C4. To address this, we performed complementary histological and imaging assays that independently validate the presence of these myelin-related glial states in human brain tissue.

First, RNAscope validation.

We performed RNAscope for *SOX10*, *GFAP*, and *C3* on human insula tissue from both control and PSP cases (Supplementary Fig. A-E). Across six randomly sampled regions per slide (> 60 astrocytes and > 100 microglial cells per slide), we quantified astrocytes (*GFAP* labeled) and microglia (*C3* positive) with detectable *SOX10* signals. Although sample number precludes formal statistical testing, we observed clear *SOX10* mRNA in a subset of astrocytes and microglia and a consistent increase of *SOX10*$^{+}$ astrocytes and *SOX10*$^{+}$ microglia in PSP relative to control, supporting the presence of myelin-glia-associated transcriptional programs in disease tissue.

Second, immunohistochemistry (IHC) validation with 3D reconstruction.

We performed IHC for PLP1 and GFAP on control (n=8) and PiD human brain samples (n=7) and analyzed confocal images using Imaris (Supplementary Fig. F-G). PLP1^{+} structures (green) and GFAP^{+} astrocytes (red) showed clear 3D colocalization, quantified using spot-distance analysis (cutoff -3 to -1 μm). Notably, PiD samples exhibited an increased number of PLP1^{+} astrocytes compared to controls, providing independent support for the existence of a myelin-interacting astrocyte population analogous to ast.C1.

Together, these RNAscope and IHC results confirm that the myelin-related glial phenotypes identified computationally, *SOX10*$^{+}$*/PLP1*$^{+}$ astrocytes and microglia, are present in human disease tissue and enriched in tauopathy contexts (PiD and PSP). These orthogonal validations support ast.C1 and mg.C4 as conserved, disease-relevant glial subpopulations rather than artifacts of the snATAC-seq dataset.

We updated this component in the manuscript (Page 23, lines 688-705):

"***In vitro* validation of disease-associated glia states**

SOX10 expression is typically repressed in microglia and astrocytes relatively to oligodendrocytes, yet we observed low levels of ectopic expression of *SOX10* and downstream programs in multiple tauopathy associated glial states, including mg.C6, mg.C4 and ast.C1. To strengthen external validation of the observed expression of *SOX10* in subpopulations of microglia and astrocytes in disease, especially PiD and PSP, we performed complementary histological and imaging assays in independent human brain samples (Methods). RNAscope for *SOX10*, *GFAP* (astrocyte marker),

and *C3* (microglia marker) in control and PSP insula tissue revealed a consistent increase in *SOX10*⁺ astrocytes and *SOX10*⁺ microglia across multiple sampled regions in PSP (Supplementary Fig. 29 A-E), supporting the presence of low levels of ectropic glial expression of *SOX10* in disease tissue. In parallel, immunohistochemistry for PLP1 (SOX10 target) and GFAP (astrocyte marker) in control and PiD brains, combined with 3D confocal reconstruction and spot-distance analysis (Methods), demonstrated *PLP1*⁺ astrocytes trend to increase in PiD, showing close spatial association between *PLP1*⁺ structures within *GFAP*⁺ astrocytes in tauopathy samples (Supplementary Fig. 29 F-G). Together, these orthogonal RNAscope and IHC validations confirm that *SOX10*⁺/*PLP1*⁺ myelin-related astrocyte and microglial states are present and enriched in human tauopathies, and a shared features of PiD and PSP."

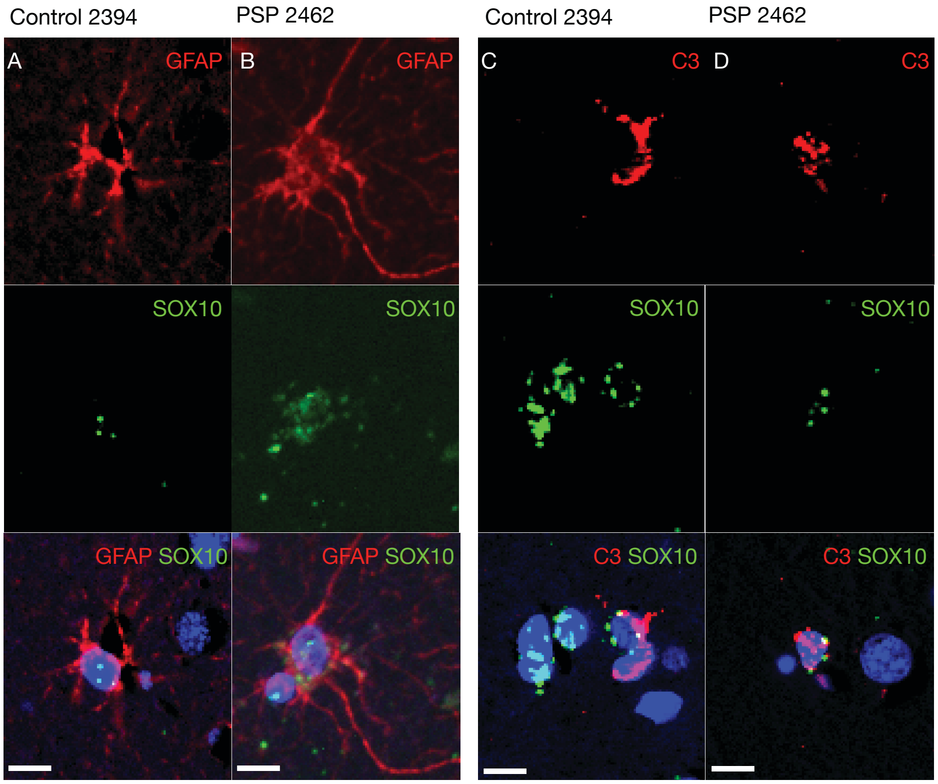

E

Percentage of SOX10+ microglia and astrocyte

	SOX10,C3/C3	SOX10, GFAP/GFAP
Control	19.0	29.2
PSP	26.9	42.6

F

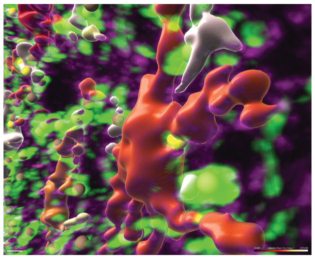

G

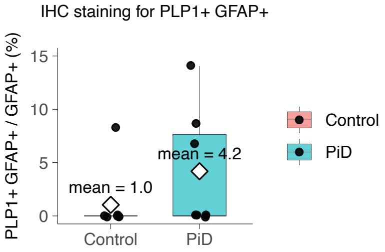

Supplementary Fig. 29. RNAscope and IHC validation of myelin-associated glia in tauopathies.
(A-B) RNAscope imaging of *SOX10* and IHC of GFAP in control and PSP insula tissue. (C-D) RNAscope imaging of *C3* and *SOX10* in control and PSP. GFAP and *C3* signals are shown in red; *SOX10* is shown in green. Scale bar: 10 μm.
(E) Quantification table summarizing the average percentage of *SOX10*$^+$ astrocytes and *SOX10*$^+$ microglia per slide, based on six randomly selected regions (> 60 astrocytes and > 100 microglial cells).
(F) High-magnification 3D confocal IHC reconstruction demonstrating close spatial association between PLP1 (green) and GFAP (red and grey). Red GFAP represents a closer proximity to PLP1, as compared to grey GFAP. Spot-distance analysis in Imaris was used to quantify PLP1-GFAP proximity, with colocalized yellow puncta representing Imaris standards of colocalization defined by a distance threshold of -3 to -1 μm.
(G) Quantification of PLP1$^+$ astrocyte percentages in control (n=8) and PiD (n=7) samples."

Meanwhile, we performed CRISPR activation of *SOX10* in iPSC-derived microglia cell lines to test the functional roles of *SOX10* we proposed from sequencing data and included the result below (Page 23, lines 707-732):

"To functionally test the role of *SOX10* in regulating microglial stress responses, we activated *SOX10* using CRISPRa in human iPSC-derived microglia and exposed these cells to PSP patient-derived synaptosomes, thereby modeling a primary tauopathy-related stress environment, followed by bulk RNA-seq profiling (Methods). In control microglia lacking CRISPRa induction, exposure to PSP synaptosomes induced robust upregulation of lysosome-associated genes linked to lysosomal stress and burden (Supplementary Fig. 30A). Exposure to synaptosomes also increases lysosome activity as measured by fluorescence imaging of active lysosomes in treated microglia. (Supplementary Fig. 30B, Methods). These findings are consistent with engagement and processing of diseased synaptic material.

Notably, CRISPRa drove limited *SOX10* expression in microglia cultured without synaptosomes. In synaptosome-treated microglia, CRISPRa induced stronger activation of *SOX10* expression and a markedly stronger transcriptional response, indicating that *SOX10* activity is selectively potentiated under stress conditions. This response closely mirrors the low-level *SOX10*-associated transcriptional programs observed in disease-enriched microglia and astrocytes in vivo, in contrast to the high *SOX10* expression characteristic of oligodendrocytes. Consistent with this stress-dependent effect, CRISPRa-*SOX10* microglia recapitulated transcriptional programs observed in human mg.C4 cells (Supplementary Fig. 30C), including coordinated induction of proliferation, cytokine signaling, and lysosomal-phagocytic pathways. Pathways enriched upon *SOX10* activation, such as myeloid leukocyte activation, Toll-like receptor signaling, and cell population proliferation (Supplementary Fig. 30D and Supplementary Table 15) closely mirrored the in vivo mg.C4 signature. Together, we validated that lysosomal stress induced by exposure to disease brain-derived synaptosomes increases the capacity of microglia to activate ectopic *SOX10* expression and its downstream gene programs. These findings support a model in which *SOX10*

acts as a stress-responsive regulator that amplifies lysosomal-phagocytic and proliferative programs in microglia upon exposure to tau-associated synaptic material."

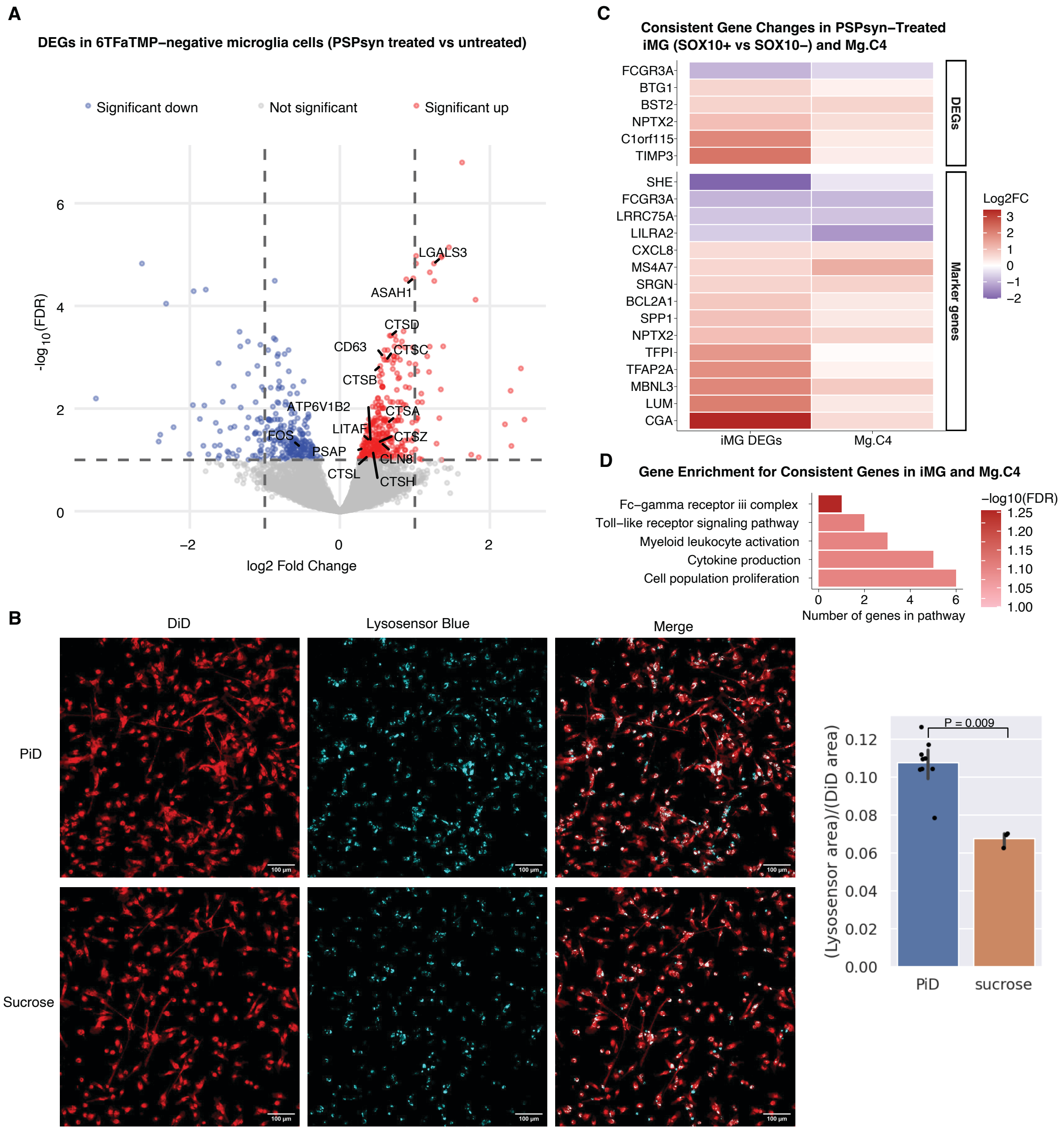

"Supplementary Fig. 30. CRISPR activation of *SOX10* in iPSC-derived microglia (iMG)under stress induces differential gene expression (DGE) resembling gene programs of myelin-related microglia in the human brain.

(A) Differential expression analysis comparing 6TFa TMP negative iPSC-derived microglia treated with PSP synaptosomes to untreated controls.

(B) Live-cell confocal microscope images of 6TFa iPSC-derived microglia treated with either 20µg/mL of PiD synaptosomes or 0.32M sucrose with protease inhibitors (vehicle) and stained

with the lipophilic dialkylcarbocyanine dye DiD (red), which dyes all lipid membranes, and LysoSensor Blue DND-167 (cyan), which fluoresces brightly in acidic organelles, especially lysosomes. Quantification of the fluorescence was calculated by taking the area of fluorescence of LysoSensor Blue within the area of fluorescence of DiD and dividing by the area of fluorescence of DiD. This represents the proportion of cellular area occupied by active lysosomes, which can be interpreted as degree of lysosome activcation. 9 images of PiD synaptosome-treated cells and 3 images of 0.32M sucrose-treated cells were compared by Wilcoxon rank sum test.

(C) The heatmap shows consistent changes in SOX10-induced DGE in iMG under stress and in myelin-related microglial markers.

(D) Functional enrichment of genes show consistent profiles in (C)."

Reviewer 2 Point 6.Potential Circularity in Cell-State Definition and Enrichment Testing. The same dataset is used to define subclusters, assign chromatin accessibility, and test GWAS heritability enrichment. While the authors attempt replication using SEA-AD data, more detail is needed about how independent the replication is. For example, are subclusters defined de novo in SEA-AD, or mapped from the discovery dataset? Explicit steps and metrics for validation across datasets should be described.

Response: We agree that avoiding circularity between defining cell states and performing heritability enrichment is essential. For this reason, all analyses, including cell-state identification, peak calling, differential accessibility, and LDSC, were conducted **independently** in the discovery and replication datasets.

In the discovery dataset, subclusters and marker peaks were defined de novo using only the chromatin accessibility profiles within that cohort. For replication in SEA-AD, we did **not** transfer or map subclusters from our dataset. Instead, we used the published SEA-AD subclass annotations and the independently curated consensus peak set generated by the SEA-AD consortium. Within this framework, we reassigned cells into six major cell types based solely on the SEA-AD metadata and recomputed disease-dynamic and stable peaks de novo across the four SEA-AD pathology stages (no AD, low, moderate, high).

LDSC was then performed **exclusively** on the SEA-AD peak sets, with no dependence on the discovery dataset. Thus, both the definition of chromatin states and the identification of heritability-enriched regulatory elements were fully independent between datasets. The concordance of AD heritability enrichment in disease-dynamic microglial peaks across both datasets therefore reflects true biological replication rather than analytical circularity. Detailed workflow diagrams and QC metrics have been added to the Methods to clarify this separation.

We updated in the manuscript (Page 11, lines 320-329):

"In the SEA-AD replication analysis, we utilized published cell subclass annotations and the consortium-defined consensus peak set to independently classify cells into six major types. For each cell type, we identified peaks that either changed across the four SEA-AD pathology stages (no AD, low, moderate, high) or remained stable. LDSC analysis was then conducted using only

the SEA-AD peak sets. We found significant enrichment of AD heritability in microglial peaks showing disease-dynamic accessibility (Supplementary Fig. 8B), with marker peaks for each pathology stage consistently enriched for AD-associated variants. These findings replicate our original results with an entirely independent dataset and analytical pipeline, confirming that disease-dynamic chromatin accessibility plays a disproportionate role in GWAS heritability."

Reviewer 2 Point 7 (1). Methodological Clarity Around Tool Usage and Parameter Justification. (1) ArchR: The use of ArchR for clustering, co-accessibility, and differential testing is appropriate, but more information is needed on parameter choices, including the justification for thresholds used (e.g., log2FC > 1.25, p < 0.001 for DARs).

Response: We have updated the descriptions in Methods (Page 40, lines 1192-1210):

"Using ArchR's iterative peak calling strategy, we generated pseudobulk replicates for each identified subcluster by grouping cells by sample using the "addGroupCoverages" function, followed by peak calling with MACS2 via the "addReproduciblePeakSet" function. The resulting peaks from all subclusters were then merged to create a consensus peak set for the entire dataset. To identify differentially accessible regions between disease and control within each cell type and brain region, we performed differential accessibility testing using the Wilcoxon test in the discovery dataset. For exploratory assessment of chromatin accessibility trends, we applied a more permissive significance threshold (P-value < 0.001) combined with a more straightforward fold-change (absolute log2 fold change >= 1.2). To ensure the robustness of DAR calling and reduce potential biases from unequal cell numbers across groups, we performed downsampling by randomly selecting 30 nuclei per sample per cell type, repeating this process 10 times, and re-running the Wilcoxon test for each iteration. To further mitigate donor-specific biases and validate our findings, we recalculated DARs using a linear mixed-effects model implemented in lmerTest::lmer with the formula: ~ Diagnosis + Number_of_fragments + TSS_Enrichment + (1 | Donor). Only peaks accessible in at least 5% of cells within a given cell type in a brain region were retained for the model analysis. This mixed-model analysis, applied to both the discovery and downsampled datasets, yielded DARs consistent with the pseudobulk analysis (P < 0.005), confirming that donor effects did not drive the observed patterns."

(2) S-LDSC (Stratified LD Score Regression): The manuscript lacks details about the annotation models used, whether the baseline model was included, and how multiple testing correction was applied across peak sets.

Response: We have updated the descriptions in Methods (Page 41, lines 1222-1236):

"We applied stratified linkage disequilibrium score regression (S-LDSC) to partition trait heritability using the baseline model v1.1, which includes 53 functional categories.

The statistical significance of τ_c^* for each annotation was assessed by computing the P-value assuming the tst statistic $\tau_c^*/s.e.(\tau_c^*)$ follows a standard normal distribution under the null hypothesis [83-85]. To correct for multiple testing, Benjamini & Hochberg's false discovery rate method was applied to adjust the P-values."

(3) ChromVAR: Variability analyses using ChromVAR are central to interpreting TF dynamics but are presented briefly. Were the motif databases manually curated for cell type-specific relevance? Clarify whether motif enrichment was adjusted for GC content or background accessibility.

Response: Thank you. The motif annotations were obtained from the curated CIS-BP human v2 database (chromVARmotifs::human_pwms_v2, 870 motifs) without additional manual selection for cell-type specificity. ChromVAR motif enrichment analyses were adjusted for GC content and background accessibility using chromVAR::getBackgroundPeaks(), which matches peaks for GC content and fragment count.

We have clarified these details in Methods (Page 45, lines 1344-1350):

"Motif annotations were obtained from the curated CISBP human v2 database TFs (chromVARmotifs::human_pwms_v2, 870 motifs) via the "addMotifAnnotations" function. Background peaks were selected with the "chromVAR::getBackgroundPeaks" function, which matches peaks for GC content and fragment count across all samples using Mahalanobis distance, thereby adjusting for GC bias and background accessibility. Per-cell deviation z-scores were computed with the "addDeviationsMatrix" function, and candidate motifs were retained if their quantile z-scores exceeded 0.5."

(4) MPRA: The study integrates MPRA results from human microglial cell lines, but it is unclear how well these lines replicate the chromatin state of in vivo microglia, especially disease-associated subtypes like mg.C4. Were chromatin marks (e.g., H3K27ac) used to validate MPRA constructs? Were control (non-regulatory) SNPs tested for specificity?

Response: Thank you for this important suggestion. We have expanded the text to clarify how the MPRA constructs were validated and how well the HMC3 cell line captures microglial regulatory features relevant to in vivo chromatin states, including disease-associated subtypes such as mg.C4.

We updated this component in the manuscript (Page 12, lines 348-381):

"**To assess transcription factor (TF) relevance**, we compared TF expression in HMC3 bulk RNA-seq (GSE219208) with snATAC-derived human microglia. We observed that 77.7% of HMC3 TFs overlapped with ~63.8% of TFs expressed in human microglia, including canonical lineage and regulatory TFs such as *ERG1, SP1*, and *MYC* (Supplementary Fig. 10A). The strong positive correlation in scaled expression values across mg.C4 and HMC3 shared TFs indicates that HMC3 cells retain core microglial transcriptional features, including identity regulators (*NFIB, KLF6, FOXP1*), chromatin accessibility TFs (*CTCF, MAX, MYC, ZBTB18*), and immune signaling TFs (*ERG1, STAT2, RBPJ*). The conserved TF architecture supports the use of HMC3 as a relevant model for testing microglia-enriched regulatory mechanisms in MPRA.

To validate MPRA constructs using chromatin histone modification marks, we first re-annotated our identified enhancers by incorporating human prefrontal cortex (PFC) H3K27ac ChIP-seq data along with the consensus human cCRE reference from ENCODE (Supplementary Fig. 5B). Using this consensus CRE reference, we found that 42.8% of frVars overlapped with active CREs, predominantly distal enhancers (dELS; 28.8%), whereas less than 1% of non-frVars overlapped (Supplementary Fig. 10C), demonstrating strong specificity and indicating that the functional variants tested in MPRA reside in bona fide enhancer contexts.

To further evaluate cell-type-specific enhancer activity, we incorporated a new single-cell Cut&Tag dataset capturing H3K27ac and H3K4me1 from AD and control brains (syn53191971). Approximately 68% of microglial CREs, including mg.C4 CREs, were supported by at least one histone modification mark (Supplementary Fig. 11A), confirming that the MPRA-tested elements overlap active enhancer states in vivo. In mg.C4, we identified CREs with consistent disease changes in chromatin accessibility and histone modifications (Supplementary Fig. 11B). The most upregulated genes are linked to ion homeostasis (*SLC24A3, LAT2*), lysosomal and protease activity (*CST7, CTSC*), and stress-related signaling *(DUSP5, BRF2*), consistent with the lysosome-enriched, stress-responsive profile of mg.C4.

Together, these analyses demonstrate that our MPRA constructs overlap validated enhancer states, exhibit disease-relevant activity in vivo, and are supported by TF expression profiles that recapitulate core microglial regulatory programs. These findings support the use of HMC3-based MPRA as an appropriate system for evaluating functional microglial regulatory variants, including those active in disease-associated states such as mg.C4."

Updated component in Methods (Page 43, lines 1280-1285):
"**Regarding specificity controls**, our MPRA design followed established best practices[89], using a scrambled sequence as the negative control rather than non-regulatory SNPs. Allele-specific effects were quantified with MPRAnalyze[90], which uses nested generalized linear models to estimate transcription rates of allele pairs and establishes the null expectation through likelihood ratio testing[48]. This design provides an internal, statistically defined baseline, making additional non-regulatory SNP controls unnecessary."

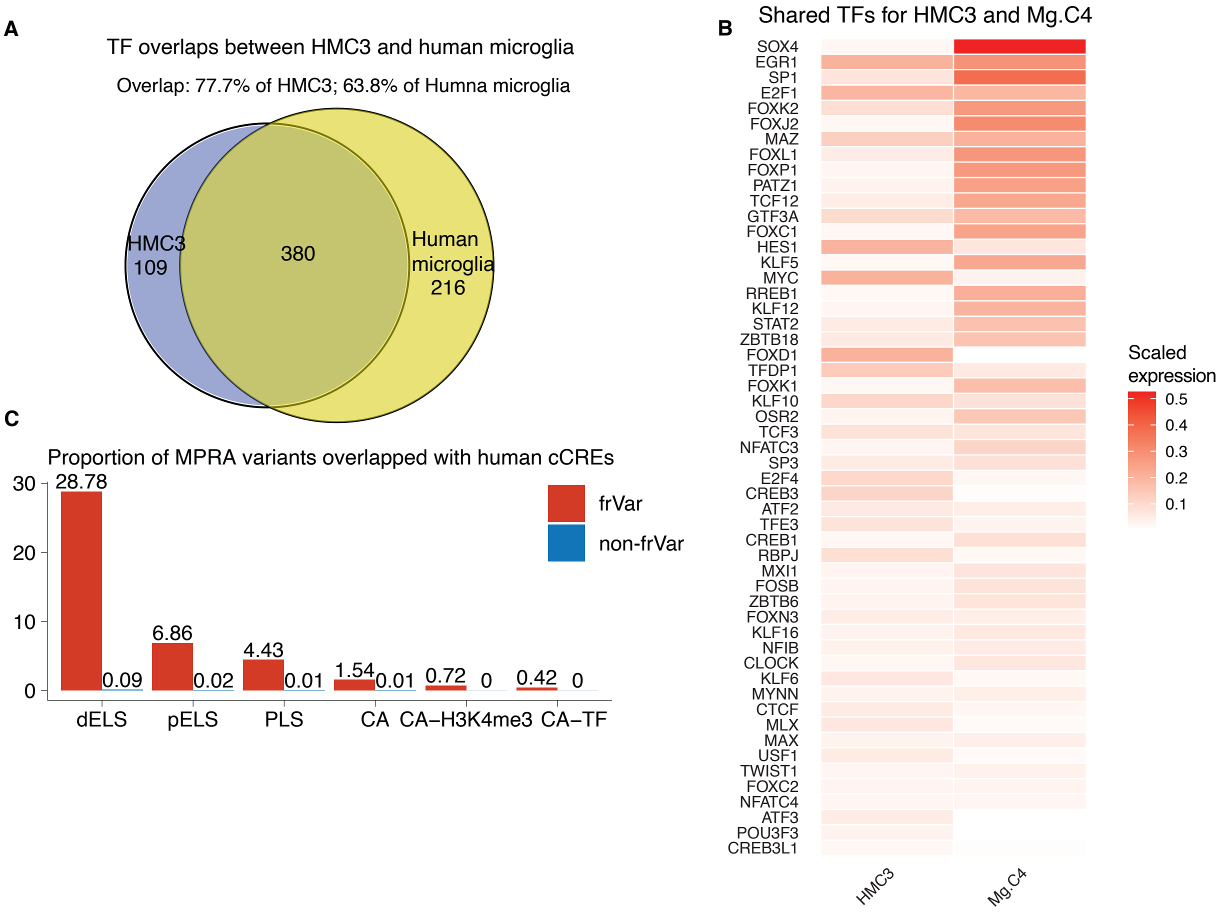

Supplementary Fig. 10. Validation of MPRA construct design.

(A) Overlap between TFs expressed in HMC3 bulk RNA-seq and human microglia. HMC3 TFs were defined as the top 50% of TFs ranked by expression level, whereas mg.C4 TFs were defined as those active in more than 10% of cells.

(B) Scaled expression and enrichment values for TFs shared between HMC3 and mg.C4, using normalized TPM values for HMC3 and enrichment false discovery rates (FDRs) for mg.C4. Values were scaled to the [0-1] range within each dataset.

(C) Overlap of MPRA-tested variants with human consensus candidate cis-regulatory elements (cCREs) from ENCODE, categorized by CRE class. CRE classification is based on biochemical signatures across biosamples and proximity to the nearest GENCODE transcription start site (TSS): distal enhancer-like signature (dELS); proximal enhancer-like signature (pELS); promoter-like signature (PLS); chromatin-accessible elements with low H3K4me3, H3K27ac, and CTCF signals (CA); chromatin-accessible elements with high H3K4me3 but low H3K27ac, not within 200 bp of a TSS (CA-H3K4me3); and chromatin-accessible elements with low H3K4me3/H3K27ac/CTCF signals that overlap transcription factor binding clusters (CA-TF).

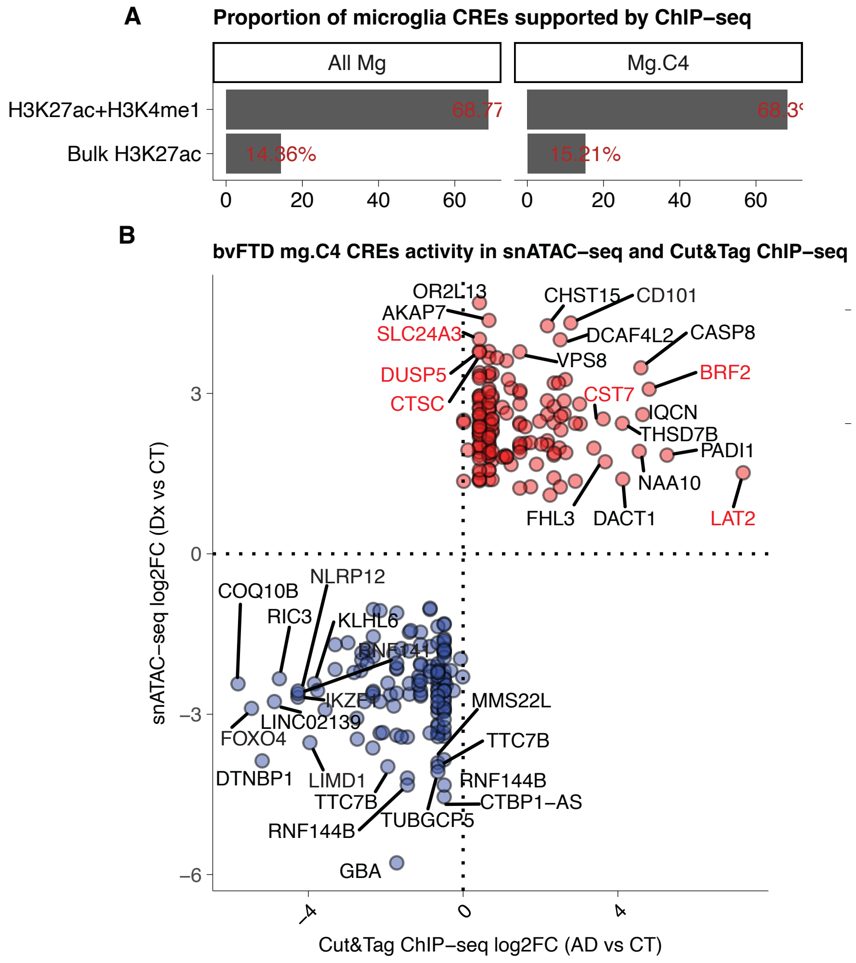

Supplementary Fig. 11. Microglia CREs refined by single-cell histone modifications.
(A) Overlap of microglial cis-regulatory elements (CREs) with histone modification marks. Bulk H3K27ac ChIP-seq data from human prefrontal cortex (PFC) (top) and a single-nucleus CUT&Tag dataset profiling H3K27ac and H3K4me1 (bottom) were used to refine enhancer annotations in all microglia and in the mg.C4 subtype.
(B) Disease-associated CRE activity in mg.C4 showing concordant changes between single-nucleus CUT&Tag data (Alzheimer's disease vs. control) and chromatin accessibility differences in this dataset (Pick's disease vs. control). Features with FDR < 0.1 are shown.

Reviewer 2 Point 8. Unclear Extent of Batch Correction and Donor Contribution Control. While the authors mention using Harmony for batch correction, additional information is needed on how donor-level effects (e.g., sample imbalance, individual heterogeneity) were handled in clustering, differential testing, and enrichment analyses. Were mixed-effect models used? How many donors contributed to each subcluster?

Response: Thank you for this helpful comment. We have clarified in the manuscript how batch correction, donor-level heterogeneity, and differential testing were addressed.

First, we used Harmony to correct technical batch effects prior to clustering while preserving true biological variability across donors. We intentionally retained donor-level structure because inter-individual variation is a meaningful biological axis in human single-nucleus data. To evaluate whether any subclusters were disproportionately driven by individual donors, we visualized UMAP embeddings colored by donor identity and quantified donor contributions for each subcluster (Supplementary Fig. 17). Across diagnostic groups, >80% of donors contributed at least 1% of cells to every subcluster, demonstrating balanced donor representation and the absence of donor-dominated clusters.

Second, for differential accessibility analysis, we used a pseudobulk Wilcoxon approach consistent with recent snATAC-seq studies. To explicitly evaluate the impact of donor variation, we repeated the analyses using a **linear mixed-effects model** with donor as a random intercept (lmerTest::lmer). This model was applied to both the full discovery dataset and the downsampled dataset to ensure robustness. The mixed-effects analysis reproduced all major results, including the gradient of gene deactivation across regions and disease states (Fig. 2L), confirming that donor effects did not drive the observed differential accessibility patterns.

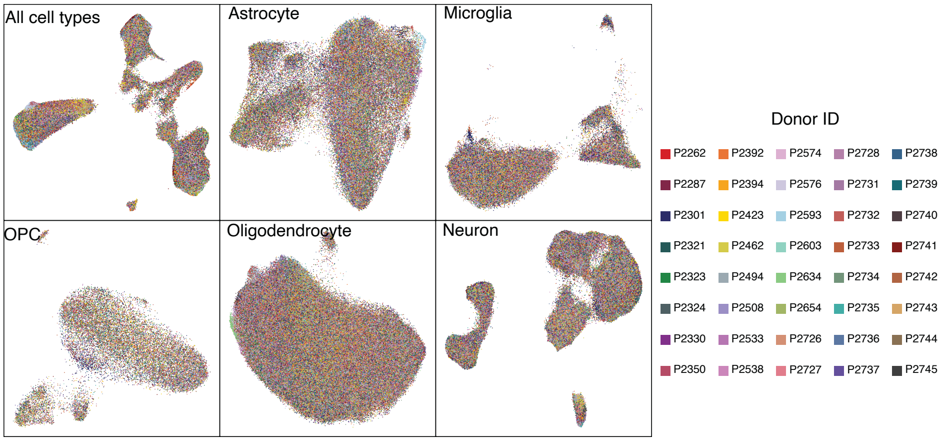

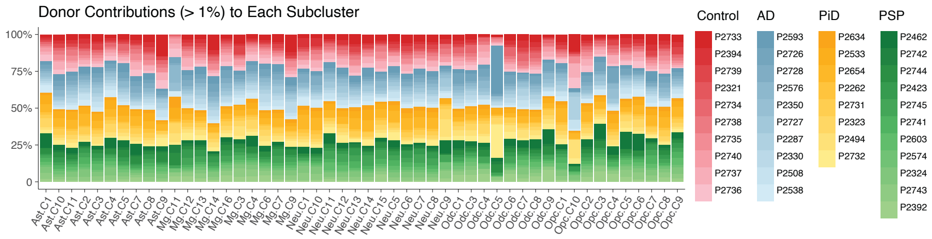

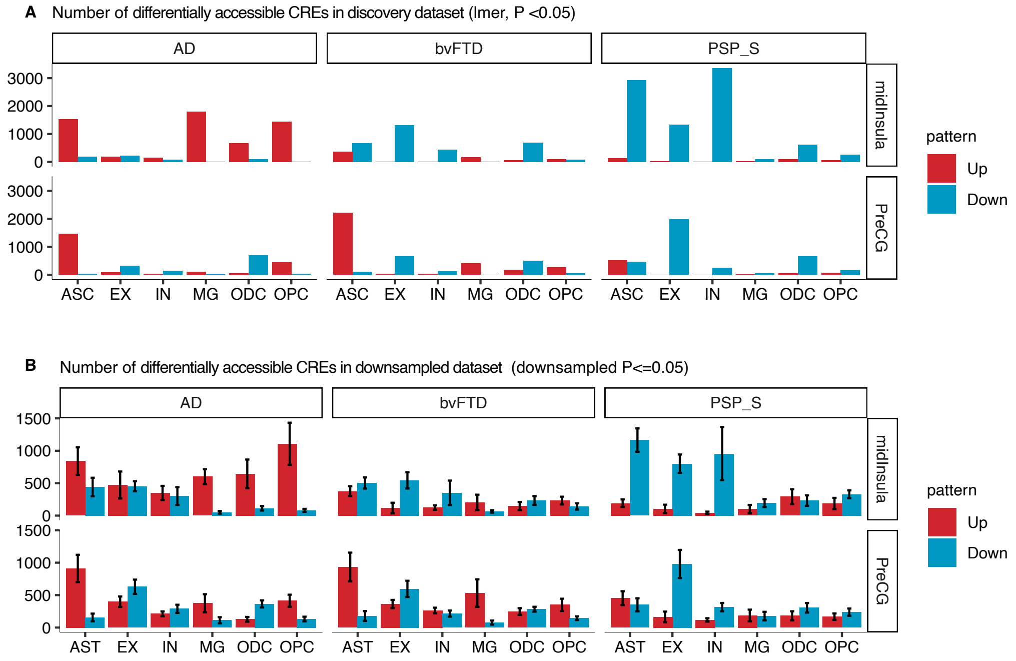

We clarified these points in manuscript (Page 18, lines 527-532):
“We used the 50 total subclusters that were achieved by combining all cells from a given major cell type across brain regions and disorders for high-resolution cellular subtyping, which we accomplished with negligible batch effect and donor effect due to our cross-disorder mixed library preparations (Supplementary Figs. 16, 17, and Methods). On average, over 80% of donors within each diagnostic group contributed at least 1% of cells to each subcluster, indicating balanced donor representation.”

We clarified these points in Methods (Page 40, lines 1203-1210):

“To account for potential donor-specific biases in differential accessibility and validate our findings, we recalculated DARs using a linear mixed-effects model (lmerTest::lmer) with the formula: ~ Diagnosis + Number_of_fragments + TSS_Enrichment + (1 | Donor). Only peaks accessible in at least 5% of cells within a certain cell type in a brain region were included in the analysis. This mixed-model analysis, applied to both discovery and downsampled datasets, yielded DARs consistent with the pseudobulk analysis ($P < 0.005$), confirming that donor effects did not drive the observed patterns.”

We clarified these points in manuscript (Page 9, lines 267-277):

“Regional gradients of gene deactivation in PSP and PiD (Fig. 2L and Supplementary Fig. 7C) were reproduced using the mixed-effects framework (Supplementary Fig. 7A). We then examined the functional effects of genes linked to shared DA-CREs between two regions, which were upregulated in the PreCG and downregulated in the insula. In PiD, deactivated genes in astrocytes were mainly associated with cellular survival and inflammation response; in oligodendrocytes, they related to immune regulation, cell migration, and apoptosis (Fig. 2M). In PSP astrocytes, deactivated genes involved blood-brain barrier functions (Supplementary Fig. 7D). Significantly,

CRE deactivation in PiD-inhibitory neurons was enriched for genes involved in synaptic plasticity (Figs. 2M-N). Similarly, in PSP inhibitory neurons, deactivated genes related to long-term synaptic depression (Supplementary Fig. 7D), indicating potential disruption in neuronal synapse response linked to disease progression in pure tauopathies."

Reviewer 2 Point 9. Conclusion Section Lacks Focus and Needs Structural Improvement. The discussion, particularly the final paragraphs, is lengthy and repetitive, which dilutes the impact of the key findings. A more structured conclusion that clearly outlines (a) major biological discoveries, (b) translational implications, and (c) limitations/future directions would significantly improve readability.

Response: We agree with the reviewer and have substantially revised the concluding section of the Discussion (Page 27, lines 830-904) to improve clarity and focus. Specifically, we restructured the conclusion into three clearly delineated components: **(a) major biological discoveries**, **(b) translational implications**, and **(c) limitations and future directions**. This revision reduces redundancy, highlights the central findings of the study, and provides a clearer synthesis of how our experimental and genetic analyses converge on disease-relevant glial regulatory programs. The revised conclusion now explicitly emphasizes the experimental validation of disease-associated glial states, the role of SOX10 in regulating phagocytic and proliferative responses, and the importance of context-specific chromatin accessibility in refining functional genetic networks relevant to tauopathies.

Minor Points and Suggestions

Reviewer 2 Point 10. Include Figure of Pathology Burden. Add a supplemental table with pathology scores by region and subject (e.g., Braak, Thal, tau burden) to reinforce disease classification and interpretation.

Response: Thank you. We have now included a supplementary figure illustrating pathology burden across brain regions (Supplementary Table 1) and a supplementary table listing pathology scores by region and subject, including neurodegeneration score and tau burden. In addition to these score, we have also now added additional available standardized neuropathological data from our samples to this manuscript (Supplementary table 1).

Reviewer 2 Point 11. Improve Legends for Cluster Names (e.g., mg.C4, ast.C1). Include descriptive names alongside cluster IDs in all figures (e.g., "myelin-related astrocyte") to avoid forcing the reader to cross-reference multiple parts of the manuscript.

Response: We agree and have corrected the legends for the cluster names in all figures and text.

Reviewer 2 Point 12. Clarify "Dynamic" Peak Definitions. Provide a simple schematic or glossary to distinguish between stable, dynamic, and DAR peaks, and explain how these were used in downstream analyses (e.g., GWAS enrichment vs. TF identification).

Response: Thank you for the suggestion. We have revised the image in Fig.1, illustrating different peak sets and their roles in downstream analysis. A glossary is added to the manuscript (Page 30, lines 915-916):

Glossary

Term	Definition
Consensus peak set	A unified collection of reproducible peaks created through an iterative overlap merging process in ArchR. Peaks are first identified in pseudo-bulk replicates per subcluster, then merged across replicates and subclusters using fixed-width (501 bp) peaks and iterative overlap removal to prevent daisy-chaining. This final set provides a stable reference for all subsequent analyses.
DAR	Differentially accessible regions where peaks show significant differences in accessibility between disease and control groups.
Marker peaks	Peaks uniquely enriched in a specific cluster compared to others.
Dynamic peaks	Peaks whose accessibility significantly varies in one condition (Control, AD, PiD, PSP) relative to the other three.
Stable peaks	Peaks with no significant changes across all conditions (control and disease groups).
CRE	Cis regulatory elements, mainly enhancers, identified via peak-peak co-accessibility and peak-gene linkage using integrated snATAC-seq and snRNA-seq.
DA-CRE	Cis-regulatory elements that differ in accessibility between disease and control groups.

Reviewer 2 Point 13. Consider Additional External Validation.
Test whether disease-specific CREs overlap epigenomic marks (e.g., H3K27ac, H3K4me1) from public datasets like Roadmap/ENCODE for relevant brain regions. Consider cross-checking TF target predictions (e.g., SOX10, MEF2C) using orthogonal datasets such as ChIP-seq.

Response: Thank you for this helpful suggestion. We expanded our analyses to include external epigenomic datasets and orthogonal TF ChIP-seq resources to further validate the activity and specificity of the disease-associated CREs.

First, we compared our disease-dynamic CREs with active chromatin states from the ENCODE/Roadmap reference (including EnhA/EnhG/EnhWk/EnhBiv and TSS-associated states). Across cell types, an average of >30% of disease-related CREs overlapped active chromatin states, supporting their regulatory potential. The moderate overlap is expected because ENCODE annotations derive from bulk brain tissue, which dilutes cell-type-specific and disease-

contextualized signals; many of our CREs only become active in tauopathy and are thus absent from healthy-tissue references.

Second, to validate enhancer activity under disease conditions, we incorporated a new single-nucleus Cut&Tag ChIP-seq dataset profiling H3K27ac and H3K4me1 in AD and control brains. In microglia, ~68% of our CREs (including both global microglia and mg.C4 CREs) were supported by at least one of these enhancer marks, and many exhibited consistent changes between AD and control, mirroring accessibility differences in PiD. This confirms that the CREs tested in MPRA correspond to bona fide enhancer elements that are dynamically regulated in neurodegenerative disease.

Third, we assessed TF-target predictions by cross-referencing TOBIAS-inferred TF binding with available human ChIP-seq datasets from Gene Transcription Regulation Database (GTRD). Although these datasets come from non-brain human cell lines (e.g., *SOX10* from melanoma; *FOXP2* and *NFIX* from generic human cell lines), we still observed ~20% overlap between predicted targets and orthogonal ChIP-seq peaks. The validated targets were enriched for developmental and motility-related processes, which were consistent with the biological context of the originating cell lines. While the overlap is moderate, this degree of concordance is expected due to the lack of matched region- and cell-type-specific ChIP-seq data for microglia or myelin-associated glia in human brain. Nonetheless, these results provide independent evidence supporting TF targets identified in mg.C4 and other disease-associated states.

Collectively, these orthogonal validations support the functional relevance of the disease-dynamic CREs and the TF regulatory programs inferred from chromatin accessibility.

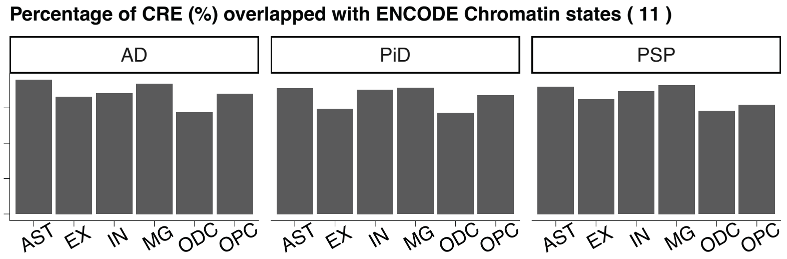

Supplementary Fig. 6. Overlap of disease-specific CREs with ENCODE chromatin states. Disease-specific cis-regulatory elements (CREs) were intersected with active chromatin states defined in the human brain by ENCODE, including active enhancers (EnhA), genic enhancers (EnhG), weak enhancers (EnhWk), bivalent enhancers (EnhBiv), active transcription start sites (TssA), flanking TSS regions (TssFlnk), upstream flanking TSS regions (TssFlnkU), downstream flanking TSS regions (TssFlnkD), and bivalent/poised TSS regions (TssBiv).

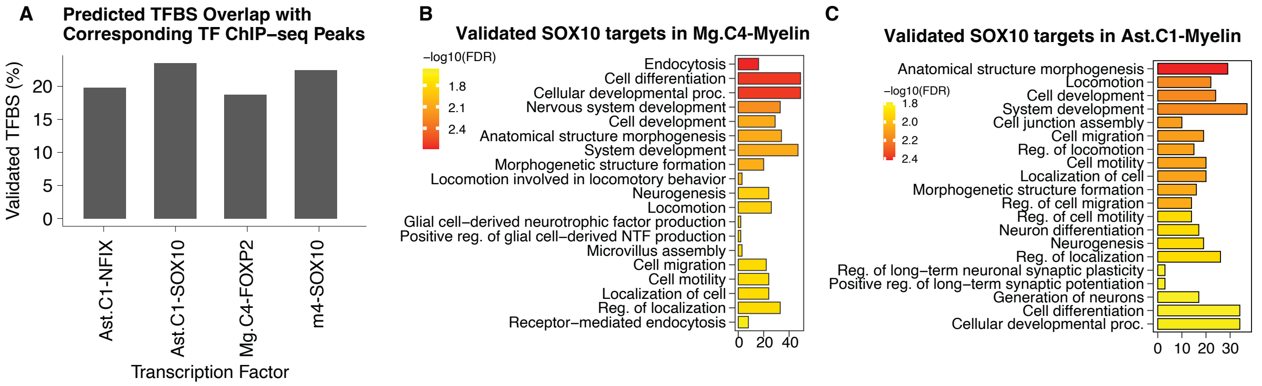

Supplementary Fig. 24. TF binding sites verified using TF ChIP-Seq data.
(A) Comparison of predicted transcription factor binding sites (TFBSs) in ast.C1 and mg.C4 with TF ChIP-seq data.
(B-C) Functional enrichment of validated TF target genes in mg.C4 (B) and ast.C1 (C). TF ChIP-seq datasets for *SOX10*, *NFIX*, and *FOXP2* were obtained from the Gene Transcription Regulation Database (GTRD) (experiment IDs: EXP034107, EXP038549, and EXP010606).

We have clarified these updates in the manuscript (Page 5, lines 146-158):
"Relying solely on H3K27ac captured 42.41% of CREs (Supplementary Fig. 5B), whereas the ENCODE cCRE reference, constructed from over 1000 cells and tissues, supported 81.6% of our identified CREs. In total, 91.2% of our enhancers were supported with the reference enhancers.

To evaluate the functional relevance of our disease-specific CREs, we compared them with active chromatin states in the human brain from the ENCODE. These states include EnhA, EnhG, EnhWk, EnhBiv, TssA, TssFlnk, TssFlnkU, TssFlnkD, and TssBiv, which were identified by integrating multiple epigenomic datasets to categorize genomic regions with similar chromatin signatures. Across cell types, >30% of CREs overlapped these active states (Supplementary Fig. 6), consistent with their regulatory potential. Moderate overlap is expected because ENCODE annotations derive from bulk brain tissue rather than cell-type- or disease-specific profiles, limiting the detection of CREs that are activated only under pathological conditions."

Updated component on Page 12, lines 367-375:
"To further evaluate cell-type-specific enhancer activity, we incorporated a new single-nucleus Cut&Tag dataset capturing H3K27ac and H3K4me1 from AD and control brains (syn53191971). Approximately 68% of microglial CREs, including mg.C4 CREs, were supported by at least one histone modification mark (Supplementary Fig. 11A), confirming that the MPRA-tested elements overlap active enhancer states in vivo. In mg.C4, we identified CREs with consistent disease changes in chromatin accessibility and histone modifications (Supplementary Fig. 11B). The most upregulated genes are linked to ion homeostasis (*SLC24A3, LAT2*), lysosomal and protease activity (*CST7, CTSC*), and stress-related signaling (*DUSP5, BRF2*), consistent with the lysosome-enriched, stress-responsive profile of mg.C4."

Updated component on Page 22, lines 649-655:
"To confirm TOBIAS's predicted transcription factor (TF) targets within subtype marker peaks, we compared these predicted binding sites with TF ChIP-Seq data from the Gene Transcription

Regulation Database (GTRD), focusing on datasets for *SOX10, NFIX*, and *FOXP2* (Experiment IDs: EXP034107, EXP038549, EXP010606). Although these data come from non-brain cell lines, approximately 20% of the predicted TF targets overlapped with orthogonal ChIP-seq peaks in our myelin-related glia subtypes (Supplementary Fig. 24), offering partial external validation of these regulatory interactions."

Reviewer #2 (Remarks on code availability):
The code is well documented and although I did not download and run it myself I did not notice any issue in making that possible.

Response: Thank you for the acknowledgement. I appreciate your careful review and feedback.

Reviewer #3 (Remarks to the Author):

Reviewer #3 Summary Comment:
Noteworthy Results
The results provide new insights into the distinct and shared epigenomic alterations in tauopathies, highlighting specific glial subtypes that may differentially contribute to disease pathology. The identification of disease-specific transcriptional regulators and the enrichment of genetic variants in certain glial subclusters are particularly noteworthy. Interestingly the authors describe a subcluster of microglia (mg.C4) being associated PiD with significant enrichment in myelin-related gene expression and increased cellular interaction, indicating its crucial role in PiD pathology. In addition, they highlight ast.C1 as a PSP-associated astrocyte subtype showing distinct chromatin accessibility with potential protective roles against tau pathology. This subcluster's unique features offers new insights into PSP-specific mechanisms and potential therapeutic targets.
Significance to the Field
This work is highly significant as it expands the understanding of the epigenomic landscape of tauopathies and identifies potential mechanistic pathways that differ across diseases. By uncovering how genetic variants may drive these changes, the study adds a new dimension to the established literature on the etiology and progression of neurodegenerative diseases.
Support for Conclusions and Claims
The authors generally support their conclusions with robust epigenomic and transcriptomic data. However, there are sections where the evidence could be better highlighted or made more comprehensible.
Flaws and Methodological Concerns. The methodology seems thorough and well-executed, leveraging state-of-the-art single-nucleus sequencing and integrating it with various genomic data.
1. Interpretation of -log10(FDR) values should be clarified, as some figures suggest non-significant results that could lead to misinterpretations.
2. Reproducibility: The methodological details are comprehensive, ensuring enough detail for reproducibility.
3. Figures and data presentation could be clearer and more consistent (e.g., ensuring appropriate coding for significance and clearer labeling).

Response: We sincerely thank the reviewer for their thoughtful and constructive evaluation. We are grateful that the novel identification of disease-specific glial subtypes, particularly the PiD-associated mg.C4 microglia and PSP-associated ast.C1 astrocytes, and their mechanistic regulatory signatures were viewed as noteworthy strengths of the study. We also appreciate the recognition that integrating chromatin accessibility, transcriptional changes, and genetic variation provides important new insight into the epigenomic architecture of tauopathies and how distinct regulatory programs may influence disease progression.

We have carefully revised the manuscript to further clarify and highlight key evidence supporting these conclusions. Specifically, we improved the interpretation and display of -log10(FDR) values, standardized figure color schemes, and explicitly stated significance thresholds in both the main text and figure legends. We also clarified when values fall below statistical significance to avoid potential misinterpretation. These updates provide clearer presentation and ensure consistency across all figures.

We appreciate the reviewer's acknowledgment of the methodological rigor and reproducibility of our workflow, and we now provide additional figure annotations and labeling improvements to further enhance clarity. Detailed responses to each of the reviewer's specific comments are provided in the point-by-point sections below.

General Comments:

• *Correct the typo "FAMTOM5" to "FANTOM5".*

Response: We apologized for the typo and have corrected it to "FANTOM5".

• *Clarify whether the context of "primarily tauopathies" indeed means "primary tauopathies".*

Response: We apologized for the error and have corrected the text to "primary tauopathies".

Results and Figures:

• *Comprehensive significance representation: Ensure p-values and FDR corrections are clearly marked, and figures indicate significant results visually.*

Response: We revised the figures (Figure 2D) to ensure that p-values and FDR thresholds are clearly marked, with significant results visually distinguished. The figure legends were also updated to explicitly state these thresholds.

• *Supplementary Figure 4C needs clarification for better understanding.*

Response: We have revised the figure legend to clarify the presentation and improve interpretability. Bars indicate the number of peaks for each cell-type combination, while dots

denote the presence of peaks in individual cell types, distinguishing cell-type-specific and shared peaks. This panel is now presented as Supplementary Fig. 5C.

• Rework Figure 3 and rename FTD as PiD or PiD/FTD to avoid confusion.

Response: Thank you for pointing this out. In Figure 3A, the label "FTD" refers specifically to the FTD GWAS from Ferrari *et al., Lancet Neurol*, 2014, which is a meta-analysis that aggregates five FTD subtypes: behavioral-variant FTD (including PiD), semantic dementia, progressive non-fluent aphasia, and FTD with motor neuron disease. Because the original GWAS reports these data under the unified label "FTD," it would be incorrect to relabel this dataset as "PiD," and we have therefore retained the term "FTD" in the figure.

However, throughout our manuscript, "PiD" is used exclusively to refer to the PiD brain samples included in our own dataset. To avoid ambiguity, we updated the Figure 3 legend to make this distinction explicit:

"'FTD' refers to the GWAS from Ferrari et al., labeled as 'FTD' in the original study and representing a meta-analysis of five FTD subtypes (including PiD, semantic dementia, progressive non-fluent aphasia, and FTD with motor neuron disease). Throughout the manuscript, 'PiD' refers specifically to the PiD samples analyzed in this dataset."

• Make modules in Figure 3I more distinguishing by using numbers as well as colors.

Response: We have revised Figure 3I as suggested.

• The discussion of MPRA and QTL enrichments needs more organization and clarity.

Response: Thank you for this helpful suggestion. We have reorganized the Results section to clearly separate MPRA- and QTL-based independent enrichment analyses and added an expanded MPRA-QTL integration analysis to more directly link functional variants with microglial regulatory programs and disease-relevant pathways (Page 16, lines 471-520). The revised manuscript now presents a stepwise analytical framework that integrates MPRA-derived functional variants (frVar), microglial eQTLs, and snATAC-seq CREs through a unified cisTopic-based approach. Meanwhile, we revised the entire Discussion section and restructured it into three clearly delineated components: major biological discoveries, translational implications, and limitations and future directions, to improve clarity and focus (Page 30, lines 830-904).

• Align text descriptions of findings more closely with visual representations.

1. Transcription Factor Variability:
• "TF variability in PSP glia deviates from other diseases, with glial types clustering naturally within PSP rather than grouping by cell types across diseases." It appears that AD also exhibits a similar but weaker clustering of transcription factor patterns in glial cells. Could this similarity

be due to the fact that PSP is characterized by 4R tau and AD by a mix of 3R/4R tau, suggesting a gradient based on the tau forms related to the disease?

Response: We agree that the gradient observed in TF variability likely reflects both the molecular form of tau pathology and its regional severity. PSP and PiD are both characterized by 4R tau and by pathology in more severely affected brain regions, and show stronger and more distinct clustering of TF activity across glial types. In contrast, AD presents milder and more heterogeneous chromatin changes across these same regions, resulting in a weaker but directionally similar gradient. This pattern suggests that TF activity tracks with a disease-specific and severity-related regulatory gradient, rather than merely a diagnostic category.

We clarified this in the manuscript (Page 4, lines 122-127):

"This pattern appears to follow a gradient linked to both tau form and pathological severity. The more pronounced clustering in PSP and PiD relative to AD may reflect their predominant 4R tau forms and the greater severity of affected regions. This supports the interpretation that TF activity aligns with a regulatory gradient coupled to disease type and extent. Conversely, excitatory neurons showed similar TF variability across all diseases, indicating that this regulatory gradient is largely glial-specific."

2. Functional Enrichment Significance:

• "Functional enrichment of cell type-specific CRE-linked genes revealed the general functions associated with the corresponding cell type identity (Fig. 2D)." The significance of certain findings in the figure, such as the enrichment of astrocyte-specific peaks in regulators of lamellipodia, is unclear. It appears that the color coding does not indicate significance clearly, making it difficult to interpret the findings for astrocytes and oligodendrocytes (ODC).

Response: Thank you for pointing out this clarity issue. We revised Fig. 2D with an improved color scale, clearer thresholding, and explicit labeling. The figure legend now explains that astrocytes and oligodendrocytes did not meet the $FDR < 0.1$ threshold. To still convey biological context without implying significance, we present their top three enriched terms by p-value ($p < 0.005$) and indicate this explicitly in both the plot and legend. Additionally, we added a supplementary figure using p-value-based color scales for all enrichments to improve interpretability.

Updated Fig. 2D legend:
"Functional GO terms for genes linked to cell type-specific CREs were analyzed using enrichR. Significant terms ($FDR < 0.1$) are shown for each cell type. Astrocytes and oligodendrocytes did not meet this threshold; therefore, their top three enriched terms based on p-value ($p < 0.005$) are shown to illustrate functional tendencies. A p-value-scaled version of the plot is provided in Supplementary Fig. 18D for reference."

3. Down-Regulated DAR Patterns:

• "With PiD showing the apparent pattern of down-regulated DARs increasing in astrocytes, inhibitory neurons, and oligodendrocytes." This pattern is not unique to PiD but is also observed in PSP while being absent in AD. Is this pattern specific to pure tauopathies, as it seems common

between PiD and PSP? If so, the functional effects of the genes linked to DA-CREs should be investigated in both diseases, not only in PiD.

Response: We thank the reviewer for highlighting this important point. We agree that the increased number of down-regulated DARs is shared between PiD and PSP and is largely absent in AD. This is consistent with the fact that PiD and PSP are primary tauopathies, whereas the precentral gyrus and insula show relatively low tau burden in AD.

This pattern remained robust when using mixed-effects models to control for donor variation (Supplementary Fig. 7A-B). Following the reviewer's suggestion, we expanded our PSP analyses to mirror those performed for PiD. We now include functional enrichment of deactivated CRE-linked genes in PSP astrocytes and inhibitory neurons (Supplementary Fig. 7).

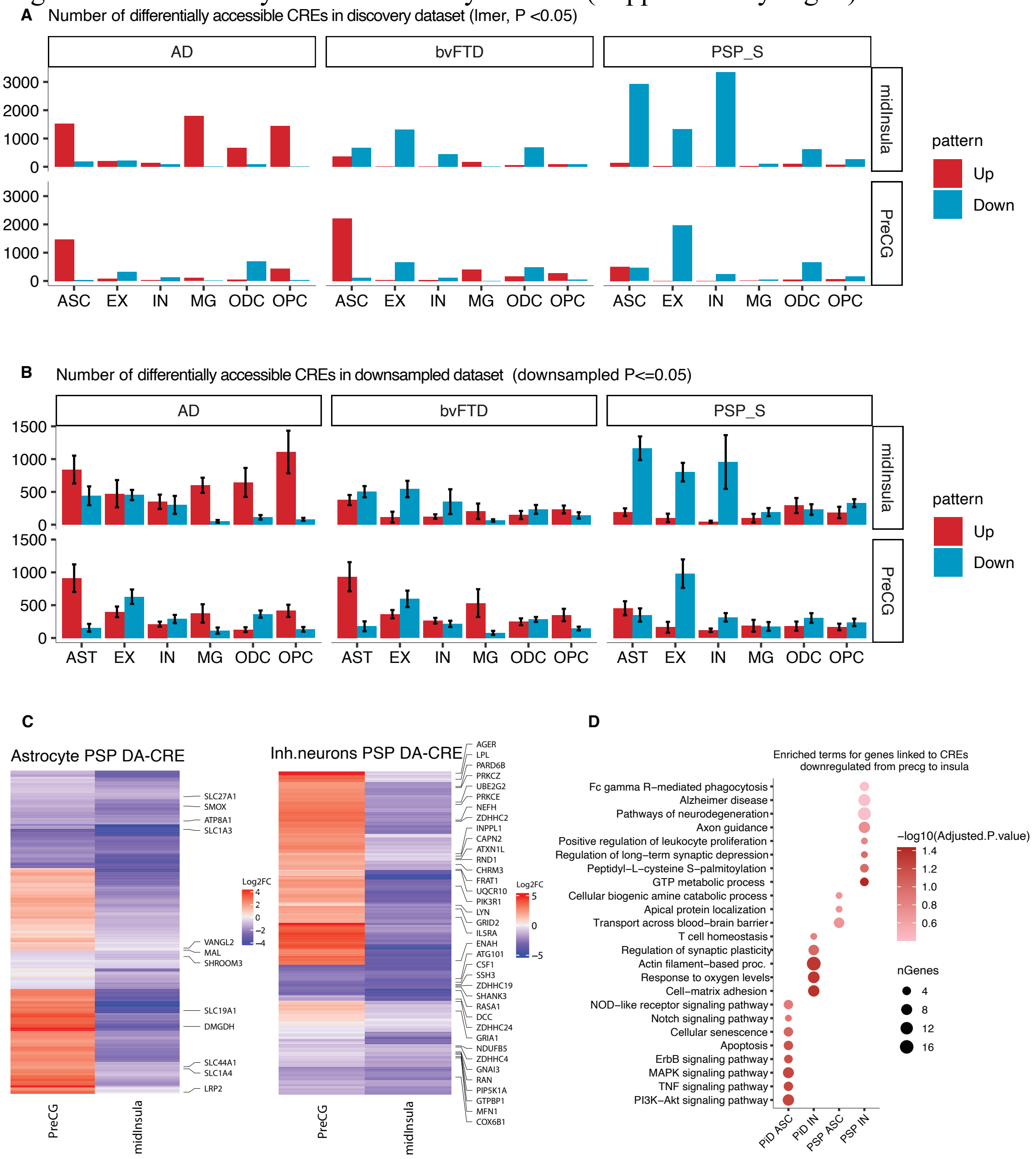

We clarified these updates in the manuscript (Page 9, lines 253-266):

"PSP also exhibited prominent gene deactivation in astrocytes and inhibitory neurons (Fig. 2L; Supplementary Fig. 5H). These patterns were robust to donor variation and were reproduced using a mixed-effects differential testing framework that included donor as a random effect (Supplementary Fig. 7). We next examined the functional effects of the genes linked to DA-CREs shared between two regions, which were up-regulated in the PreCG but down-regulated in the insula. In PiD, the deactivated genes in astrocytes were primarily associated with cellular survival and inflammation response, while in oligodendrocytes, they were linked to immune regulation, cell migration, and apoptosis (Fig. 2M). In PSP, deactivated genes in astrocytes were involved in blood-brain barrier functions (Supplementary Fig. 7). Notably, CRE deactivation in PiD inhibitory neurons was enriched for genes that control synaptic plasticity (Figs. 2M-N). A similar trend appeared in PSP, where deactivated genes in inhibitory neurons were associated with long-term synaptic depression (Supplementary Fig. 7), suggesting a common disruption of synaptic regulation across pure tauopathies."

4. Cluster-Specific Upregulation:

• *It is challenging to determine if the upregulation observed in distinct clusters is specific to the subclusters listed by the authors (Figure 4J and G). While it is clear for HLA-DMB, it is not as evident for the other markers listed. Could the authors further elucidate these findings to clarify the upregulation patterns within these subclusters?*

Response: Thank you for pointing this. To improve clarity regarding which subclusters exhibit specific upregulation, we revised the figure in several ways. We included arrows pointing directly to the subclusters with increased marker expression and updated the heatmap of marker genes across all subclusters to make the expression patterns more visually explicit. These improvements clearly show that the upregulation patterns are specific to the subclusters discussed in the text.

REVIEWER COMMENTS

Reviewer #1 (Remarks to the Author):

I appreciate the authors having gone to great lengths to strengthen the paper. My initial point of the MPRA results being underexplored is not valid anymore - the integration of frVars with sn-eQTLs was a good idea. Revisions suggested by the other reviewers also add value to this paper, and the discussion is a lot easier to read now.

The authors correctly identified several studies having previously examined open chromatin architecture in AD and PSP, but they are neither cited, nor discussed - this should still be corrected.

Response: We thank the reviewer for the positive assessment and helpful suggestion. We have now incorporated the relevant studies and expanded the discussion in the Introduction with appropriate citations (PMID: 37774678, 34239132, 37774680, 35976433, 39648200, 35931864, 36950385, 31727856, 40752494) on Page 3, lines 71-80:

"To understand the underlying regulatory mechanisms, large-scale single-cell transcriptomic and epigenomic profiling studies have begun to characterize widespread remodeling of cis-regulatory elements and transcription factor programs in vulnerable brain cell types in AD and related tauopathies[10,17,18,28–33]. We recently demonstrated diverse shared and distinct cellular responses to three tau-associated disorders, including an Alzheimer's-enriched microglia state with high expression of genes associated with AD polygenic risk together with genes known to protect against AD-specific pathology [19,34]. It remains unclear whether glial subpopulations have distinct mechanisms underlying their diverse roles in disease pathogenesis across different tau dementia disorders, and how disease-associated chromatin remodeling integrates genetic risk and regulatory variation across different tauopathies and brain regions."

Reviewer #2 (Remarks to the Author):

The author clearly addressed all of my concerns and the manuscript in my opinion is suitable for publication.

Reviewer #2 (Remarks on code availability):

The code is well documented

Reviewer #3 (Remarks to the Author):

Authors answered all my questions appropriately. No further comments.

Response: We thank Reviewers #2 and #3 for their positive evaluations and for confirming that all concerns have been addressed.
